# Patient preferences for inflammatory bowel disease treatments: protocol development of a global preference survey using a discrete choice experiment

**DOI:** 10.3389/fmed.2024.1418874

**Published:** 2024-08-14

**Authors:** Elise Schoefs, Séverine Vermeire, Marc Ferrante, João Sabino, Bram Verstockt, Luisa Avedano, Maria Stella De Rocchis, Magdalena Sajak-Szczerba, Roberto Saldaña, Noortje Straetemans, Martina Vandebroek, Rosanne Janssens, Isabelle Huys

**Affiliations:** ^1^Department of Pharmaceutical and Pharmacological Sciences, KU Leuven, Leuven, Belgium; ^2^Department of Gastroenterology and Hepatology, University Hospitals Leuven, KU Leuven, Leuven, Belgium; ^3^Department of Chronic Diseases and Metabolism, KU Leuven, Leuven, Belgium; ^4^European Federation of Crohn's & Ulcerative Colitis Associations (EFCCA), Brussels, Belgium; ^5^Department of Gastroenterology, AZ Vesalius, Tongeren, Belgium; ^6^Belgian IBD Nurses and Study Coordinators Association (BINAStoria), Brussels, Belgium; ^7^Faculty of Economics and Business, KU Leuven, Leuven, Belgium

**Keywords:** inflammatory bowel disease, patient preferences, focus group discussions, attributes, levels, discrete choice experiment, healthcare decision-making, drug development

## Abstract

**Background:**

As the therapeutic landscape for inflammatory bowel disease (IBD) continues to expand, a need exists to understand how patients perceive and value different attributes associated with their disease as well as with current and emerging treatments. These insights can inform the development and regulation of effective interventions for IBD, benefiting various stakeholders including healthcare professionals, drug developers, regulators, Health Technology Assessment bodies, payers, and ultimately patients suffering from IBD. In response to this, the present patient preference study was developed with the aim to (1) determine the relative preference weights for IBD treatment and disease related attributes, and (2) explain how preferences may differ across patients with different characteristics (preference heterogeneity).

**Methods:**

The patient preference study (PPS) was developed through an 8-step process, with each step being informed by an advisory board. This process included: (1) stated preference method selection, (2) attribute and level development (including a scoping literature review, focus group discussions, and advisory board meetings), (3) choice task construction, (4) sample size estimation, (5) survey implementation, (6) piloting, (7) translation, and (8) pre-testing. The resulting discrete choice experiment (DCE) survey comprises 14 attributes with between two and five varying levels. Participants will answer 15 DCE questions with a partial profile design, where each of the choice questions encompasses two hypothetical treatment profiles showing four attributes. Additionally, questions about patients' socio-demographic and clinical characteristics, as well as contextual factors are implemented. The survey is available in 15 different languages and aims to minimally recruit 700 patients globally.

**Discussion:**

This protocol gives valuable insights toward preference researchers and decision-makers on how PPS design can be transparently reported, demonstrating solutions to remaining gaps in preference research. Results of the PPS will provide evidence regarding the disease and treatment related characteristics that are most important for IBD patients, and how these may differ across patients with different characteristics. These findings will yield valuable insights applicable to preference research, drug development, regulatory approval, and reimbursement processes, enabling decision making across the medicinal product life cycle that is aligned with the true needs of IBD patients.

## Introduction

Inflammatory bowel disease (IBD) is a chronic condition of the gastrointestinal tract that is characterized by recurring inflammation (1, 2). The most common symptoms include abdominal pain, (bloody) diarrhea, urgency, weight loss, and fatigue (1). As currently IBD cannot be cured and patients switch between intermittent episodes of remission and flares, life-long treatment is usually required and quality of life of patients is significantly impacted (3, 4).

In recent decades, the therapeutic landscape of IBD has seen a sharp increase in the number of available treatments for patients, each characterized by their own side-effects, mode of administration, mechanism and speed of action, treatment schedule, and uncertainties (5). However, despite considerable therapeutic advancements, only one-third of patients attain short- to medium-term remission (6). Therefore, multiple new treatments are in the pipeline for IBD with treatment characteristics that differ from those on the market. This raises uncertainties and questions about how patients perceive and value these novel treatment characteristics (7).

Patient preferences hold an important role in determining the relative value of different therapeutic options and their characteristics (8). Patient preferences refer to how desirable or acceptable to patients a given alternative or choice is among all the outcomes of a medicine and can be elicited via patient preference studies (PPS) (9). To date, limited PPS have been conducted regarding the preferences of IBD patients concerning disease and treatment characteristics in development and use (10). Further, most existing studies are product agnostic, conducted in a single country, and/or were performed before newer treatments such as Janus Kinase (JAK) inhibitors or Sphingosine 1-phosphate (S1P) Receptor Modulators became available (10–12). Consequently, it remains unclear how IBD patients worldwide value different treatment and disease related characteristics. However, as research around new treatments for IBD evolves, understanding patient preferences around these characteristics becomes important to inform the development and evaluation of effective IBD interventions. This information can offer valuable insights for stakeholders involved in decision-making including healthcare professionals, drug developers, regulators, Health Technology Assessment (HTA) bodies, and payers.

Performing a PPS in IBD can inform decision making by (1) quantifying the value that patients attach to treatment characteristics, (2) giving additional insights into the acceptability of uncertainties and side-effects to patients, and (3) providing patients a role to weigh in on decision making (8, 13–16). As a result, treatments can be developed that are accepted by patients, which has been shown to improve patients' treatment adherence and potentially enhance their quality of life (17, 18). To be able to inform different decision-making processes, PPS must be conducted in a scientifically robust manner. To this end, The Patient Preferences in Benefit-Risk Assessments during the Drug Life Cycle (PREFER) project created evidence-based recommendations and a PPS framework to guide stakeholders in the organization, design, conduct, and analysis of PPS and how these results can be used to inform decision-making (19). This PREFER PPS framework received a positive European Medicines Agency (EMA) Qualification opinion, reflecting regulatory acceptance on the suitability of the framework (20). Nevertheless, implementation of this PPS framework across disease domains is needed to increase confidence and practical experience. Future research should also address remaining (methodological) uncertainties, including how to reduce the cognitive burden of PPS surveys, how to enhance stakeholder and patient involvement in PPS design and conduct, and how to transparently select and describe attributes and their levels (21–23).

To increase evidence in the field of preference research and to investigate IBD patients' preferences, a global PPS following the PREFER EMA qualified PPS framework has been initiated; the protocol of which is reported in this manuscript. The survey established through this protocol serves to meet the needs of diverse decision makers across the medicinal product life cycle and aims to: (1) determine the relative preference weights for IBD treatment and disease related attributes, and (2) explain preference heterogeneity by investigating how preferences may be influenced by socio-demographic characteristics, clinical characteristics, and contextual factors. In the current protocol, special attention was given to the description of the involvement of stakeholders in the design and testing of the PPS and the process undertaken for the selection of attributes and levels for inclusion in the preference questions, serving to increase transparency regarding PPS research choices with practice examples.

## Methods and analysis

**Figure 1:**
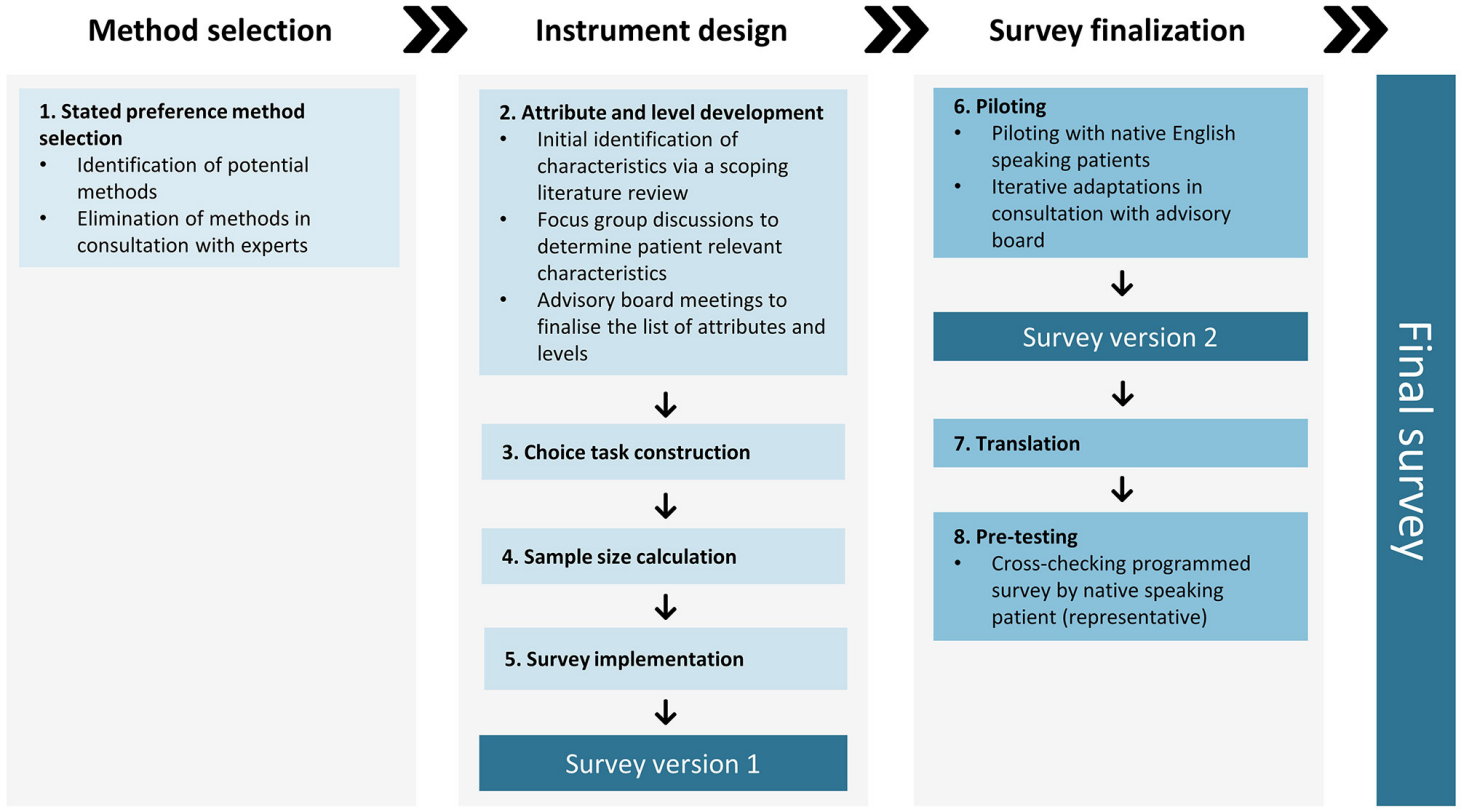
Eight steps of the patient preference survey development according to the PPS EMA qualified framework.

The protocol of the patient preference survey was developed in eight sequential steps following the PREFER EMA qualified framework (Figure 1) (19). Throughout the different steps of the protocol development, an advisory board was consulted to discuss the aims and methodology of the PPS and to ensure that the questions were relevant, understandable, and plausible for IBD patients. The advisory board consisted of patients (*n* = 2), patient representatives from the European Federation of Crohn's & Ulcerative Colitis Associations (EFCCA, *n* = 2), gastroenterologists specialized in IBD (*n* = 4), an IBD nurse (*n* = 1), and a statistician (*n* = 1) from different European countries (Belgium, Italy, Poland, and Spain). Details on how the advisory board was specifically involved are provided in each of the different steps described below.

### Step 1: stated preference method selection

Method selection started from the five elicitation methods that were identified by the PREFER EMA qualified framework as being the ones most likely to meet most decision-makers' needs during all stages of the medicinal product life cycle: discrete choice experiment (DCE), Best-Worst Scaling Case 1 and 2, (Probalistic) Threshold Technique, and Swing Weighting (20). Together with preference research experts, the criteria of these five elicitation methods were compared to our research question, patient population (chronic condition with high prevalence), and decision-making context (multiple treatment options available, each with their own characteristics). We wanted the method to: (1) quantify the relative importance of different attributes, (2) elicit the trade-offs patients make among multiple benefits and risks simultaneously, (3) explain preference heterogeneity; to investigate how preferences are influenced by socio-demographic characteristics, clinical characteristics, and contextual factors, (4) investigate patients preferences for treatment (characteristics) that are not yet available, and (5) allow incorporation in an unsupervised online survey that can be disseminated across the world.

When comparing the criteria of the five different methods to our requirements using the PREFER recommendations (19), best-Worst Scaling Case 1 and 2 were eliminated as possible methods as they do not allow to estimate trade-offs between attributes and levels. Moreover, as described in step 2, the qualitative research and advisory board meetings yielded 14 attributes. For each attribute, two to five levels needed to be included in our survey. As the (Probalistic) Threshold Technique only allows eight attributes and cannot calculate the relative importance of attributes, this method also did not meet our study needs. From the two remaining promising methods, swing-weighting was excluded in consultation with preference research experts as it is often implemented with qualitative workshop formats which was deemed difficult in the context of a global online preference survey and due to the complexity of the choice task with 14 attributes. In comparison, DCE allows to apply a partial profile design which increases the feasibility of the choice task with 14 attributes. As a result, DCE was chosen as the preference elicitation method for this study. As described in Box 1, the characteristics of the DCE method fit our study requirements.

Box 1 **DCE characteristics based on the PREFER recommendations and PREFER EMA qualification (19, 20).**

1. Estimates weights (relative importance) for attributes and changes in their levels
2. Estimates trade-offs between attributes
3. Quantifies heterogeneity in preferences
4. Can assess maximum acceptable risk (MAR) and minimum required benefit (MRB)
5. Can incorporate internal validation methods
6. Can assess interactions between attributes (preference dependence)
7. Resembles trade-offs made in clinical practice
8. Allows incorporation (and computing) of current and future potential treatment characteristics
9. Public acknowledgment as acceptable method to study preferences
10. No interaction between participants needed to complete

DCE is a stated preference method based on random utility theory (RUT) (24). In a DCE survey, respondents are asked to complete a set of stated preference choices or “choice tasks” (25). In each choice task, respondents are presented with hypothetical treatment profiles defined by attributes and their levels and asked to select their preferred alternative (20, 26). RUT expects that respondents use complex and rational decision-making processes when completing these choice tasks (24). Based on the choices across a series of questions, statistical inferences can be made to determine the trade-offs respondents are willing to make for each attribute and the relative importance (weights) for each attribute level can be estimated (20).

### Step 2: attribute and level development

Bridges et al. (27) recommend that attribute identification should be supported by evidence on the potential range of preferences and values patients hold and should be guided by a literature review. Further, Coast et al. (28) and Hollin et al. (29) recommend systematic and rigorous qualitative research to develop attributes for inclusion in discrete choice experiments. Therefore, to determine the attributes and levels for inclusion in the patient preference survey, three sequential phases were executed, were each phase informed the subsequent phase: (1) a scoping literature review to identify initial characteristics, (2) focus group discussions (FGDs) with IBD patients to determine patient relevant characteristics, and (3) advisory board meetings to finalize the attributes, levels and their descriptions for inclusion in the preference survey. The full methodology and results of the literature review and FGDs are described in a separate paper (30).

#### Initial identification of characteristics via a scoping literature review

A scoping literature review was performed to identify previous published PPS, available IBD treatments, and IBD clinical trials in order to reveal treatment and disease characteristics for discussion during the subsequent FGDs with patients (see Section 2.2.2) and to inform subsequent decisions on attribute and level selection (see Section 2.2.3). First, attributes used in previous published PPS conducted among IBD patients were identified by searching PubMed and Embase. Search terms consisted out of derivations of following Mesh terms: “inflammatory bowel disease” AND “patient preferences” AND “preference elicitation/exploration method.” Publications reporting results of PPS were identified and included if published after 2011 and if a preference method was applied. Second, favorable and unfavorable aspects of current treatments for IBD were extracted from the European Public Assessment Reports (EPARs) or the products' leaflet (31). Lastly, to identify the primary and secondary endpoints and adverse events of therapies which are currently in development, phase 3 IBD clinical trials (both completed, ongoing, and restarted) in the European Union from 2011 till onwards were screened using the Clinicaltrialsregister (32). In this manner, also potential “future” treatment outcomes and adverse events could be integrated in the discussion. The results of the literature review, together with a comprehensive description of the applied search queries, and in- and exclusion criteria can be found in Supplementary material 1.

In total, 22 PPS, 58 phase 3 IBD clinical trials, and 45 IBD treatments met our inclusion criteria. From these sources, a list of characteristics possibly important to IBD patients was compiled and grouped in three overarching categories: (1) characteristics related to the treatment efficacy, (2) characteristics related to the administration of the treatment, and (3) characteristics related to symptoms of the disease or complications and side effects of treatments. Furthermore, an explanation for each characteristic was added. Subsequently, this list was reviewed by members of the advisory board to ensure comprehensibility, completeness, and clinical plausibility. Based on their feedback, different alterations in wordings were made, the characteristic “mucosal healing” got split into “macroscopic healing of the intestinal mucosa” and “microscopic healing of the intestinal mucosa” and following 8 characteristics were added: (1) transmural healing of the gut, (2) improved quality of life, (3) improved work rate, (4) occurrence of lethargy, (5) occurrence of abnormal liver function, (6) occurrence of abnormal renal function, (7) development or worsening of diabetes, and (8) development of associated inflammatory diseases. In the end, a list was composed of 55 treatment and disease related characteristics ready to be graded and discussed by the participants in the FGDs (Table 1—the full list with characteristics and associated definitions as given to the participants in the FGDs can be found in Supplementary material 2).

**Table 1:** Overview of the 55 initial characteristics.

**Characteristics**	**Source**		
	**Previous IBD PPS**	**Available IBD treatments**	**Phase 3 IBD clinical trials**
**Characteristics related to the treatment efficacy**			
Short-term clinical response	✓	✓	✓
Long-term clinical remission	✓	✓	✓
Prevention of flare-ups	✓		✓
Macroscopic healing of the intestinal mucosa^*^	✓	✓	✓
Microscopic healing of the intestinal mucosa^*^	✓	✓	✓
Transmural healing of the gut^*^			
Use of cortisone preparations	✓		✓
Use of painkilling medication			✓
Avoiding hospitalization	✓		✓
Avoiding surgery	✓		✓
Improved quality of life^*^	✓		✓
Improved work rate^*^	✓		
**Characteristics related to the administration of the treatment**			
Mode of administration	✓		
Frequency of treatment	✓		
Frequency of follow-up	✓		
Location of treatment	✓		
Indication of IBD on the package leaflet	✓		
**Characteristics related to symptoms of the disease or complications and side effects of treatments**			
Occurrence of abdominal pain and cramps	✓	✓	✓
Occurrence of blood in the stool	✓	✓	✓
Occurrence of sudden need to relieve oneself	✓	✓	✓
Occurrence of incontinence	✓	✓	✓
Occurrence of indigestion	✓	✓	✓
Occurrence of diarrhea	✓	✓	✓
Occurrence of vomiting	✓	✓	✓
Occurrence of nausea	✓	✓	✓
Occurrence of abnormal liver function^*^		✓	✓
Occurrence of abnormal renal function^*^		✓	✓
Development or worsening of diabetes^*^		✓	✓
Changes in body weight	✓	✓	✓
Loss of appetite		✓	✓
Occurrence of fatigue	✓	✓	✓
Occurrence of lethargy^*^			
Occurrence of fever		✓	✓
Occurrence of headache		✓	✓
Occurrence of dizziness		✓	✓
Occurrence of neuropathy		✓	✓
Appearance of skin rash	✓	✓	✓
Occurrence of hypersensitivity to UV rays		✓	✓
Occurrence of hair loss	✓	✓	
Occurrence of bone or back pain		✓	✓
Occurrence of joint pain	✓	✓	✓
Occurrence of muscle pain		✓	✓
Development of osteoporosis		✓	✓
Reduction of eyesight		✓	✓
Occurrence of hypertension	✓	✓	✓
Occurrence of anemia	✓	✓	✓
Occurrence of insomnia	✓	✓	✓
Occurrence of depressive mood		✓	✓
Occurrence of anxiety		✓	✓
Occurrence of serious infections	✓	✓	✓
Occurrence of infusion reactions	✓	✓	✓
Development of cancer	✓	✓	✓
Development of associated inflammatory diseases^*^			
Construction of an ostomy	✓		
Construction of a pouch	✓		

PPS, patient preference study; IBD, inflammatory bowel disease. ^*^Characteristics were included after feedback from the advisory board.

#### Focus group discussions to determine patient relevant characteristics

To determine patient relevant characteristics for inclusion in the patient preference survey, two FGDs with IBD patients (*n* = 11) using the nominal group technique (NGT) were held (30). Ethical approval was granted from the Ethics Committee Research UZ/KU Leuven in Belgium (S65034). A discussion guide following the four stages of the NGT was designed, reviewed by members of the advisory board resulting in minor text edits, and subsequently used during both FGDs. Each discussion was executed by the same moderator (ES) and took place in the participants' native language (Flemish). Evidence based guidelines and best practices for conducting FGDs and the NGT following Krueger et al. (33) and McMillan et al. (34) were applied.

During the FGDs, a top-down technique consisting of a grading exercise to question participants about the importance of the 55 characteristics (see Section 2.2.1) was combined with a bottom-up technique including open questions to detect new characteristics not identified during the literature review. During the open questions, characteristics were identified by asking patients to freely elaborate on aspects that they found important in their treatment and/or disease. Following characteristics were in depth discussed and found important: gastrointestinal symptoms, reduced energy, changes in physical appearance, skin manifestations, vision problems, avoidance of surgery and the need for an ostomy, long-term effects of medication, fast clinical response, mental and psychological support, and normal social interactions. After the open discussions, the results of the grading exercise which participants were asked to complete prior to the discussion were shown and further elaborated on. The five characteristics with the highest mean score were: prevention of surgery, long-term clinical remission, improved quality of life, occurrence of urgency, and improved labor rate (Table 2—full results can be found in Supplementary material 3).

**Table 2:** Top 10 characteristics identified in the grading exercise.

**Rank**	**Characteristic**	**Mean score**
1	Prevent surgery	9.55
2	Long-term clinical remission	9.45
3	Improved quality of life	9.36
4	Occurrence of urgency	9.27
5	Improved labor rate	9.27
6	Occurrence of diarrhea	9.18
7	Occurrence of severe infections	9.18
8	Prevent hospitalization	9.09
9	Occurrence of joint pain	9.00
10	Prevent flare-ups	9.00

#### Advisory board meetings to finalize the list of attributes, levels and their descriptions

During a meeting with the advisory board composed of stakeholders from various European countries, the findings from the FGDs were presented and extensively discussed. This collaborative meeting provided insights into interpreting the obtained results within a clinical context, adding to our qualitative dataset alongside the FGDs with Belgian patients. Combining their input and critical reflections with the outcomes of the literature review and the FGDs, consensus was reached to include attributes in the survey that were most important to patients, with emphasis on quality of life attributes. Subsequently, an initial list of potential attributes, levels, and their descriptions was created, which formed the basis for subsequent multi-stakeholder discussions and revision rounds.

The multi-stakeholder discussions and iterative revision rounds resulted in the deletion of 2 characteristics that were raised during the FGDs: vision problems and the occurrence of joint pain. These characteristics were deleted because according to the advisory board, they are deemed as rare (vision problems) or not related to IBD or the treatment thereof (occurrence of joint pain). To ensure the attributes meet the criteria of clarity, distinctiveness, and unambiguity, we excluded “long-term clinical remission,” “improved quality of life,” “improved labor rate,” “preventing flare-ups,” and “normal social interactions” from the list. This decision was based on several factors: (a) they overlapped with other attributes, serving as overarching themes encompassing more specific elements, and (b) their interpretation could vary among different patients (27). Further, as gastrointestinal problems were deemed key and overarching for particular problems, the researchers decided to break these problems into 3 different attributes namely (1) frequency of having to go to the toilet, (2) urgency and pain of having to go to the toilet, and (3) severity of daily abdominal pain and cramps. Likewise, the decision was made to include the attribute “risk of undergoing surgery,” wherein the possibility that surgery may be accompanied by the construction of a (temporary) stoma was mentioned as patients during the FGDs found the risk of having a stoma an important characteristic. Moreover, “reduced energy,” “changes in physical appearance,” “skin manifestations,” “mental and psychological support,” “occurrence of severe infections,” and “fast clinical response” were rephrased to “severity of fatigue,” “duration of severe physical changes,” “duration of severe skin problems,” “severity of psychological impact,” “risk of serious infection,” and “how fast the treatment will work” respectively as these wordings were found to be more comprehensible to patients while meeting the criteria for attribute development (27).

Although not among the top 10 characteristics identified, sleeping-related issues emerged as a noteworthy concern during the FGDs. Consequently, the decision was made to incorporate “frequency of sleeping problems” as an additional attribute within the survey. Similarly, “method of administration,” ranking 53^rd^ among the 55 characteristics, was initially perceived as less critical during the FGDs. However, given the ongoing diversification of IBD treatments, encompassing subcutaneous, intravenous, oral, rectal, and on-body-injector devices, along with pharmaceutical companies' pursuit of innovative delivery methods, assessing the potential influence of this attribute on patients' decision-making was deemed useful for informing benefit-risk assessments and health technology evaluations. Therefore, the researchers decided to include the attribute “how the treatment is administered” as an attribute. Furthermore, recognizing the pivotal role of endoscopic remission as a primary endpoint in clinical trials and clinical decision-making for therapy continuation or discontinuation, the attribute “presence of visual signs of inflammation in the bowel” was included. Finally, based on advisory board input, “decreased libido” found its place in our survey despite not emerging as a topic during the FGDs, possibly due to its sensitive nature for group discussions.

At the end of the discussions and revision rounds, the advisory board reached consensus to incorporate 14 attributes into the survey, each accompanied by explanatory descriptions. These attributes and their descriptions underwent further validation and refinement by our advisory board, which ensured content validity, patient relevance, and the use of appropriate language to minimize cognitive load. Additionally, for each attribute, a comprehensive range of two to five levels was established. These levels were determined through a rigorous process that considered clinical plausibility, drawing upon insights from clinical experts within our advisory board, evidence gained from the existing literature, and insights derived from ongoing clinical trials investigating novel IBD treatments. The final list of attributes, their respective levels, and accompanying descriptions, can be found in Table 3.

**Table 3:** Final list of attributes, their respective levels, and accompanying descriptions for inclusion in the patient preference survey.

**Attributes**	**Descriptions**	**Levels**
Risk of undergoing surgery	This is the risk that you need to undergo surgery because: •Medical therapy cannot adequately control your intestinal inflammation •You have recurrent flares •There is a puncture in the wall of your bowel (perforation), a narrowing in a part of your bowel (stricture), or a pus-filled area in your bowel (abscess) •There is a high risk of cancer in the bowel •There is cancer in the bowel Surgery may be accompanied by the construction of a (temporary) stoma.	•LOW risk: 2 out of 100 people (2%) who take this treatment will need surgery •HIGH risk: 10 out of 100 people (10%) who take this treatment will need surgery
Frequency of having to go to the toilet	This is the frequency that you have to go the toilet.	•Normal frequency, similar as prior to the diagnosis of inflammatory bowel disease •High frequency, more as prior to the diagnosis of inflammatory bowel disease
Urgency and pain of having to go to the toilet	This is the urgency that you have to go to the toilet and the pain that you experience with it.	•No urgency and no pain •High urgency and high pain
Severity of daily abdominal pain and cramps	This is the severity of abdominal pain and cramps you may experience daily.	•No pain and no cramps •Moderate pain and moderate cramps •Severe pain and severe cramps
Severity of fatigue	This is the severity of an overwhelming sense of tiredness, lack of energy, or feeling of exhaustion that is not relieved after rest or sleep.	•Mild: not limiting your usual activities such as work, study, housework, family, social or leisure activities •Moderate: moderately limiting your usual activities such as work, study, housework, family, social or leisure activities •Severe: severely limiting your usual activities such as work, study, housework, family, social or leisure activities
Frequency of sleeping problems	This is the frequency you may experience sleeping problems such as difficulty falling asleep, difficulty staying asleep, early morning awakening, or awakening during the night.	•Once a month or less •Once a week •Every night
Severity of psychological impact	This is the severity of the following psychological impact that you may experience: •Feeling anxious •Feeling confused and/or disorientated •Feeling more quickly irritated •Feeling not able to focus/concentrate •Having a low mood •Having mood swings	•Mild: not limiting your usual activities such as work, study, housework, family, social or leisure activities •Moderate: moderately limiting your usual activities such as work, study, housework, family, social or leisure activities •Severe: severely limiting your usual activities such as work, study, housework, family, social or leisure activities
Risk of serious infection	This is the risk that you may experience a serious infection. Serious means that the infection: •May have consequences that persist for months after treatment ends such as permanent damage to organs •May require hospitalization or multiple hospital visits •May occur gradually or suddenly	•LOW risk: less than 1 out of 100 people (1%) who take this treatment will experience a serious infection •HIGH risk: 10 out of 100 people (10 %) who take this treatment will experience a serious infection
Duration of severe physical changes	This is the duration of one of the following changes in your physical appearance that you may experience •Severe weight loss or gain •Development of a moon face (swollen round face) •Increased facial hair (e.g., mustache or beard) •Hair loss •Etc.	•Temporary; a maximum of 3 months •Permanent; life-long
Duration of severe skin problems	This is the duration of one of the following severe skin problems that you may experience: •Dry skin •Eczema •The multiplying of skin cells resulting into bumpy red, flaky, crusty patches of skin (psoriasis) •Rash and itchy bumps •Acne •Inflammation of the fat cells under the skin •Areas of swelling under the skin •Degeneration (the process by which tissue deteriorates and loses its functional ability) and thinning of the skin •Development of (red) spots, stretch marks (striae), or ulcers on the skin •Heightened skin sensitivity or unusual reaction when your skin is exposed to UV radiation (photosensitivity)	•Temporary; a maximum of 3 months •Permanent; life-long
Decreased libido	Whether or not you have a decreased libido or sexual desire. This can be caused by: •Your medication •Your symptoms such as urgency or abdominal pain •The psychological impact of inflammatory bowel disease	•No: normal libido as before you had inflammatory bowel disease •Yes: lower libido than you had before inflammatory bowel disease
How the treatment is administered	This is the way that the treatment is administered to you on a regular basis.	•Oral •Intravenous (via a needle - you need to go to a clinic) •Subcutaneous (via a needle - you can do this at home) •Rectal; this can be a suppository, a rectal foam, or an enema •On-body injector device attached with a patch to your skin that injects the medical treatment into your body
Presence of visual signs of inflammation in the bowel^*^	Whether or not you have visual signs of inflammation in your bowels during an endoscopic examination. It can be that you have no visual signs of inflammation in your bowel but still have clinical symptoms.	•No •Yes
How fast the treatment will work	This is the time between the administration of the medicine and the improvement of the symptoms of the disease. An improvement means that your treatment was able to lessen your symptoms to the point where they are mostly absent or gone and reduce the signs of inflammation in: •Your blood or stool •Your digestive tract •Your biopsy (the removal of cells or tissues for examination)	•Fast reduction of symptoms (within first 2 weeks after starting the treatment) •Slow reduction of symptoms (3 months after starting the treatment)

In the survey, each attribute and level are accompanied by a graphic. For example, percentages are visually presented using 100 human figures, with specific figures highlighted in color to represent the given percentage. ^*^Attribute was renamed from “long-term endoscopic remission” to “presence of visual signs of inflammation in the bowel” after the piloting phase (see Step 6).

### Step 3: choice task construction

In each DCE choice task, respondents are presented with two hypothetical treatment profiles and asked to select their preferred alternative. During the attribute development phase (see Section 2.2), a total of 14 patient-relevant attributes were identified. Considering that health-related DCE studies typically employ 4–6 attributes, we opted for a partial profile DCE design to reduce the complexity of the choice tasks (35). In this design, each choice task encompasses 4 of the 14 attributes, with each attribute having 2 to 5 varying levels. Furthermore, to minimize the difficulty of the survey and to ensure a manageable task for participants, we included a maximum of two unlabelled treatment alternatives displayed as “Treatment A” and “Treatment B.” No opt-out option was included to preserve preference information in each DCE choice task and to prevent loss of information when participants opt-out.

The combination of attribute levels and treatment options presented in each DCE choice question were generated by Sawtooth Software's randomized balanced overlap design. This random design includes some degree of attribute level overlap, which has shown to reduce dropout rates, to increase the level of choice consistency, and to avoid learning effects (36). Further, the use of attribute level overlap is a recommendable strategy to reduce attribute non-attendance e.g., ignoring one or more of the included attributes, resulting in deeper preference information than minimal overlap (37, 38). Dominant alternatives where the attribute levels of one alternative are clearly better than the attribute levels of the other alternative were manually excluded from the balanced overlap design. In the final survey, each respondent received a set of one of the 295 combinations of choice tasks.

### Step 4: sample size estimation

A priori sample size calculations for DCE experiments remain a significant challenge (35). Typically, DCE questionnaires necessitate larger sample sizes compared to other preference elicitation methods, often exceeding 250 participants (20). The minimal required sample size is contingent upon various factors such as the complexity of the choice tasks, the number of DCE questions, preferred precision of the results, the need for subgroup analysis, and the desirability to measure interactions. Recognizing this, we explored the capabilities of Sawtooth's test design option, which provides sample size calculations including estimated precision of the results based on choice task complexity and the number of DCE questions, revealing an inverse relationship between the latter and the anticipated sample size. This led to a trade-off discussion with our advisory board, weighing the number of expected respondents with the amount of DCE choice questions that we could include while keeping the survey as short and cognitively manageable as possible. Ultimately, the advisory board anticipated to be able to recruit minimally 700 patients globally. This estimation was made based on previous experience with quantitative research in the targeted patient population and the global disease burden of IBD, which is estimated to be between 2.5 and 3 million people in Europe alone (3). Consequently, each participant in our survey will need to complete 15 choice questions, aligning with ISPOR guidelines for conjoint analysis applications in health (27). This strategy was implemented to secure statistically significant responses and to achieve our study objectives.

### Step 5: survey implementation

The overall survey was implemented in Lighthouse studio with Sawtooth software (39). The survey consisted out of 10 parts: (1) language selection, (2) introduction to the survey, (3) screening questions, (4) information sheet and informed consent form, (5) explanation of the attributes and levels included in the survey, (6) DCE questions (see Section 2.3), (7) feedback on the DCE questions, (8) validation question, (9) questions on patients socio-demographic characteristics, clinical characteristics, and contextual factors, and (10) survey evaluation questions.

Questions on patients' socio-demographic characteristics (e.g., age, age of diagnosis, sex, and work status), clinical characteristics (e.g., disease status, current treatment, previous treatment, and surgical history), and contextual factors (e.g., distance to from location were followed for treatment, involvement in treatment decision-making, knowledge about disease) will be asked as such that preference heterogeneity can be explained. Further Chews' set of brief screening questions were included to determine the health literacy of participants and to identify patients who may face challenges in comprehending medical information (40). All questions were formulated in collaboration with the advisory board, providing valuable input to improve patient relevance and optimize wordings, ultimately reducing cognitive burden while ensuring clinical relevance. The advisory board also reviewed the information sheet and informed consent form. The validation question and time to complete the survey were built into the survey as validity checks.

### Step 6: piloting

The programmed survey was piloted with four native speaking English patients. Online pilot interviews were organized using the think-aloud method that evaluated (1) patients' comprehensibility of the general survey questions, (2) patients' understanding of the choice questions (including the attributes and their descriptions) and the explanation preceding the choice questions, (3) patients' choice behavior, and (4) the length of the survey. After each interview, feedback was discussed with the advisory board until consensus was reached and the survey was iteratively adapted before the next pilot. After the fourth pilot, no further changes were deemed necessary, and the piloting stopped.

Overall, participants thought that the survey was easy to read, had a good flow, and was user-friendly with graphics supporting the content. Utilizing the suggestions and opinions shared by participants, the first information page was made more concise and emphasize was placed on the option to receive background information by clicking on specific words. Moreover, some repetitions were highlighted, and the questions were adapted accordingly. Lastly, the validation question was adjusted from “Which 3 characteristics are most important to you” to “In the previous questions we asked you to choose between two treatments that differed according to the characteristics that are listed below. Which 3 characteristics do you think are most important and did you consider the most when answering the previous questions?”. Besides textual errors in the demographic questions, which were rectified promptly, participants did not report any other concerns regarding the general questions.

Participants found the DCE questions to be “Very easy” or “Easy” to understand. However, some participants indicated that it was “Difficult” to answer the choice questions, explaining that this was due to the nature of the questions and the complex trade-offs they had to make. Based on the feedback during the pilots, the explanation proceeding the choice questions was adapted to provide more clarification. In addition, the attribute “achievement of long-term endoscopic remission” was renamed to “presence of visual signs of inflammation in the bowel” as the original attribute was deemed not clear. Further, an explanation for the word “striae” in the explanation of the attribute “duration of severe skin problems” was added and some revisions in the wording of the levels and explanations were carried out. Participants found the survey length “manageable” or “too long,” with two participants needing more than 30 min to complete the survey. These participants may have required more time due to the additional task of evaluating the survey.

During the pilots, special attention was given to the choice behavior of participants to detect the possible occurrence of simplifying heuristics e.g., choice processes that do not adhere to the normative rationality assumptions implied by RUT as participants apply simplifying decision rules. During the pilot interviews, participants did not show simplifying heuristics that are common in (health-related) DCEs such as attribute non-attendance (respondents completely ignore certain attributes), choice set formation (elimination of choice alternatives based on certain threshold levels for particular attributes), and lexicographic preferences (selecting alternatives entirely based on their superiority on one most important attribute) (24).

### Step 7: translation

The final English survey was translated by a native speaking researcher of this study or national patient organization member into Arabic, Croatian, Danish, Dutch, Finnish, French, German, Greek, Hungarian, Italian, Polish, Portuguese, Romanian, and Spanish (total languages = 15). A mix between North, East, South, and West European languages was chosen to increase heterogeneity and inclusiveness of participants in terms of different European regions, ensuring representativeness of the patient sample. Furthermore, the survey was translated to Arabic, English, French, Portuguese, and Spanish as such that also patients outside of Europe were able to complete the survey.

### Step 8: pre-testing

After programming the survey into the 15 different languages in Sawtooth, the advisory board and native speaking patients (representatives) that helped with the translations cross-checked the programmed survey during the pre-testing phase to final test the functionality of the survey program and identify textual errors. No significant issues were identified, and only minor textual revisions were implemented. The final survey can be found in Supplementary material 4.

### Recruitment

The survey will be widely disseminated across the world to reach a large and heterogenous sample of IBD patients. Patients will be recruited via the IBD umbrella patient organization EFCCA, national patient organizations, and local clinicians. These recruiting parties will disseminate the survey link via mail, newsletters, social media, and face-to-face hospital visits.

The survey includes initial questions to assess the eligibility of the participants using the following criteria:

- Patients diagnosed with Crohn's disease, ulcerative colitis, or inflammatory bowel disease type unclassified;
- Adult patients (≥18 years old);
- Able to understand the language used in the survey; and
- Approval of the informed consent.

### Data analysis plan

The main goal of the patient preference survey is: (1) to determine the relative preference weights for IBD treatment and disease related attributes, and (2) to explain preference heterogeneity by investigating how preferences may be influenced by socio-demographic characteristics, clinical characteristics, and contextual factors.

To determine the preferences of IBD patients, attribute (level) estimates, and the relative importance of each attribute, a logit-based analysis strategy will be applied and conditional logit (CL), latent class (LC), and mixed logit (ML) models will be fitted. The final decision on modeling will be made once data collection is finished. This decision will be made based on the model's conformity to the data and its clinical interpretation. It is possible that different models will be used to address the various research questions. Preference heterogeneity and the impact of socio-demographic characteristics, clinical characteristics, and conceptual factors will be explained by applying LC analysis and/or subgroup analysis. All results above will be compared between countries, gender, and IBD subtype.

Descriptive analysis using mean and standard deviations will be used to summarize the responses on the closed survey questions. Responses to the open questions will be analyzed qualitatively using the thematic analysis (41).

## Discussion

This protocol paper describes the development of a global patient preference survey in IBD which aims to (1) determine the relative preference weights for IBD treatment and disease related attributes, and (2) explain how preferences may differ across patients with different characteristics. This protocol outlines methodological and practical steps for quantitatively eliciting patient preferences regarding IBD treatments and disease related aspects, adhering systematically and rigorously to design principles in DCE research and following the PREFER EMA qualified framework (19). This survey will be the first global PPS in IBD to follow the PREFER EMA qualified framework. From a methodological viewpoint, this protocol gives valuable insights toward preference researchers, clinicians, and decision-makers on how PPS design and PPS research choices can be transparently reported. Our study can serve as an example for PPS researchers, illustrating how to articulate attribute and level selection alongside the choice for the preference elicitation method. By delineating each PPS research choice and its rationale, we aim to minimize decision-makers' inquiries regarding design choices and bolster regulators' and health technology assessment bodies' confidence in study outcomes, enhancing their utility in decision-making processes.

The five elicitation methods identified by the PREFER EMA qualified framework as being the ones most likely to meet most decision-makers' needs during all stages of the medicinal product life cycle, were used as a starting point to select the preference elicitation method for our PPS (20). However, while the EMA qualification opinion on IMI PREFER emphasizes that the PREFER selection provides valuable guidance, it underscores the importance of not confining method selection exclusively to the options outlined in this framework, stating the non-exhaustive character of this list (42). Therefore, it is imperative to maintain flexibility in method selection to accommodate potentially more suitable alternatives. Considering our research objectives and attribute considerations together with the input from preference experts and our advisory board, we ultimately did opt for a DCE in light of the many possibilities this elicitation method offers including estimating the trade-offs between attributes. Nevertheless, it remains essential to continue exploring additional (simplistic) methods beyond those specified in the PREFER framework to facilitate PPS survey completion, minimize dropout, and to enhance methodological knowledge to refine preference elicitation strategies further.

A patient-centered approach was used to create the preference survey; patients and patient representatives were involved across all the different steps of the survey development. This guaranteed that the survey meets the real needs of the patient population and ensures that the background questions, attributes, levels, and their descriptions were relevant, understandable, and plausible for IBD patients. Patients and patient representatives could namely reflect on their experiences and how they would describe certain characteristics in a way that is understandable for patients. Further, based on the FGDs and advisory board meetings, we decided to not only include treatment related attributes, but also (non-conventional) attributes related to patients' disease such as “frequency of sleeping problems,” “severity of psychological impact,” and “decreased libido” with a focus on quality of life related attributes. The inclusion of these attributes distinguishes our study from previously PPS, which primarily focus on treatment-related characteristics and may overlook quality of life related attributes that truly matter to patients (10). This approach also allows to investigate the trade-offs patients make between treatment-related characteristics and disease- and quality of life-related attributes. Further, by actively involving patients in both the development (writing of lay-language questions, selection of number of questions, etc.) and pilot testing of the survey, the cognitive burden of the survey and response error could be minimized, in line with findings by Mes et al. (43). The advisory board also played a crucial role in the key trade-off that had to be between the number of expected respondents with the amount of DCE choice questions that we could include while keeping the survey as short and cognitively manageable as possible. Here, the patient organization representatives ultimately made the choice and deemed 15 DCE questions feasible for each patient to complete. Furthermore, the decision to incorporate visual elements alongside text in explaining attributes not only enhanced respondent comprehension but also fostered engagement by mitigating potential boredom and reducing cognitive strain.

Findings of this study will yield valuable insights applicable to preference research, drug development, regulatory approval, and reimbursement processes. For example, information on PPS data can be integrated in the clinical overview, the EPARs, and other relevant documents to support regulatory decisions and benefit-risk assessments (42). Consequently, the insights gleaned from this study will enable patient-centered decision-making throughout the medicinal product life cycle, ensuring alignment with the true needs of IBD patients.

## Ethics and dissemination

### Regulatory and protocol compliance

Ethics approval was granted by the Ethics Committee Research UZ/KU Leuven for both the focus group discussions (S65034), as well as the conduct of the patient preference survey (S65998). The privacy and ethical team of KU Leuven (PRET) also approved the data management plan of the study. The study will be conducted in compliance with this protocol and guidelines for Good Clinical Practice. Personal data will be processed in accordance with the European General Data Protection Regulation (AVG/GDPR) 2016/679.

### Ethical considerations

Prior to the focus group discussions, all participants provided their informed consent. To protect participants' privacy, all participants were identified by a number and not by their name. After transcription, all audio recordings were destroyed. Survey participants will be informed that their participation in the patient preference survey is pseudonymous (no names or IP addresses will be asked, however, responses in the open text fields may enable the researchers to recognize participants) and have the possibility to read the information sheet outlining detailed information about the study. They will then be asked to provide electronic informed consent before they can answer any further questions in the survey. It is possible that participants may become distressed thinking about IBD and the associated impact that the disease or its treatment may have or has had on their lives. Therefore, we will provide participants sources for further information and contact details for support.

### Publication and dissemination plan

Results of the study will be presented and thoroughly discussed with the advisory board. Findings will be made publicly available via international peer-reviewed journals and further communicated via clinical and health economical conferences. A lay-language summary of the results will be written for patients and made available to patient organizations and other recruiting parties for distribution on their own channels.

## Ethics statement

The studies involving humans were approved by Ethics Committee Research UZ/KU Leuven (S65034 and S65998). The studies were conducted in accordance with the local legislation and institutional requirements. The participants provided their written informed consent to participate in this study.

## Author contributions

ES: Conceptualization, Data curation, Formal analysis, Funding acquisition, Investigation, Methodology, Project administration, Resources, Software, Supervision, Validation, Visualization, Writing – original draft, Writing – review & editing. SV: Conceptualization, Methodology, Supervision, Writing – review & editing. MF: Conceptualization, Writing – review & editing. JS: Conceptualization, Writing – review & editing. BV: Conceptualization, Writing – review & editing. LA: Conceptualization, Writing – review & editing. MD: Conceptualization, Writing – review & editing. MS-S: Conceptualization, Writing – review & editing. RS: Conceptualization, Writing – review & editing. NS: Conceptualization, Writing – review & editing. MV: Conceptualization, Writing – review & editing, Methodology, Supervision. RJ: Conceptualization, Methodology, Supervision, Writing – review & editing. IH: Conceptualization, Funding acquisition, Methodology, Supervision, Writing – review & editing.
