# Supplementary material for: Patient preferences for inflammatory bowel disease treatments: protocol development of a global preference survey using a discrete choice experiment
Source: Front Med (Lausanne). 2024 Aug 14;11:1418874. doi: 10.3389/fmed.2024.1418874 (PMC11349669; doi:10.3389/fmed.2024.1418874)
Supplement: Supplementary file 1 [file Table_1.DOCX]

***Supplementary Material 1***

**Patient Preferences for Inflammatory Bowel Disease Treatments: Protocol Development of a Global Preference Survey using a Discrete Choice Experiment**

# 1A - Previous published patient preference studies in inflammatory bowel disease

**Database used:** Pubmed and Embase

**Date of search:** 28 March 2021 (Pubmed) and 31 March 2021 (Embase)

Selection based upon title and abstract

All publication dates

**Inclusion criteria:**

- Preferences from IBD patients

**Exclusion criteria:**

- If preferences not from patients (only caregivers, clinicians)
- If no preference method (qualitative/quantitative) was applied^[[1]](#footnote-1)^
- If no preferences reported
- Study protocols
- Review articles
- If preferences elicited *only* for non-medical product-related treatment characteristics (e.g. for participation in decision-making, for remote monitoring, for information, physical activity)

**Search query Pubmed:**

(patient* preference*[Title/Abstract]) AND ((inflammatory bowel disease[Title/Abstract]) OR (Crohn's disease[Title/Abstract]) OR (ulcerative colitis[Title/Abstract])) AND ((interview* [Title/Abstract]) OR (discussion* [Title/Abstract]) OR (survey*[Title/Abstract]) OR (discrete choice experiment*[Title/Abstract]) OR (swing weighting*[Title/Abstract]) OR (preference method*[Title/Abstract]) OR (ranking*[Title/Abstract]) OR (qualitative study*[Title/Abstract])OR (quantitative survey*[Title/Abstract]) OR (rating*[Title/Abstract]) OR (threshold technique*[Title/Abstract]) OR (preference elicitation technique*[Title/Abstract]))

**Results Pubmed:**

40 results, 15 included

**Search query Embase:**

'patient preference' AND ('inflammatory bowel disease' OR 'crohn disease' OR 'ulcerative colitis') AND ('interview' OR 'discussion' OR 'survey' OR 'discrete choice experiment' OR 'swing weighting' OR 'preference method' OR 'ranking' OR 'qualitative study' OR 'quantitative survey' OR 'rating' OR 'threshold technique' OR 'preference elicitation technique')

**Results Embase:**

128 results, 7 included

**Combined results (n=22)**

| First author, year | Research objective | Sam-ple | Preference method(s) | Attributes/items/criteria | Results |
| --- | --- | --- | --- | --- | --- |
| Almario, 2018 | (1) To quantify and rank order the relative importance of biological attributes that drive decision making and (2) to characterize the heterogeneity among patients regarding preferences for biologic therapies, and to evaluate whether any demographic or disease characteristics may predict preferences. | 640 IBD patients | Adaptive choice-based conjoint survey | 9 attributes in 4 categories; (i) biologic mechanism of action; (ii) mode of administration (i.e., route and frequency); (iii) efficacy — long-term remission, short-term improvement; and (iv) side effect profile — tolerability of side effects (i.e., chance of stopping the medication because of side effects), fatigue, rash, risk of serious infection, risk of lymphoma | The algorithm revealed that 41.3% respondents valued treatment efficacy as the most important attribute category in their decision making, followed by 38.3% for side effect profile. In contrast, 20.0% and 0.5% individuals valued mode of administration and mechanism of action, respectively, as the predominant factor.  For UC patients the average attribute importance was; long-term remission (15.6%), route/frequency of administration (15.3%), lymphoma risk (13.7%), short-term improvement (12.8%), serious infection risk (11.1%), tolerability of side effects (10.4%), rash (10.0%), fatigue (7.4%), mechanism of action (3.6%).  For CD patients the average attribute importance was; short-term improvement (15.1%), lymphoma risk (14.7%), and route/frequency of administration (13.7%), long term remission (13.0%), serious infection risk and tolerability of side effects (12.0%), rash (8.4%), fatigue (7.4%), mechanism of action (3.7%). |
| Boeri, 2019 | (1) To identify the UC treatment attributes that patients and physicians consider most important when making treatment decisions and why and (2) to elicit and quantify patient and physician preferences for the attributes of moderate to severe UC treatments that emerged from the  qualitative interviews | (1) 15 UC patients and 16 gastroenterologists and (2) 200 UC patients and 200 gastroenterologists | (1) Individual interviews using a semi-structured interview guide and (2) DCE survey | NA | During the qualitative interviews, 7 attributes were identified; (i) time until symptoms improve, (ii) probability that UC symptoms are under control after 1 year, (iii) annual risk of serious infection, (iv) 5-year risk of malignancy, (v) mode of administration, (vi) dosing schedule, and (vii) need for occasional use of steroids.  The DCE survey showed that patients prioritize symptom control over time to symptom improvement. For patients, all pill-dosing schedules were more preferred to both subcutaneous injections and intravenous infusions; however, less frequent dosing schedules were not always preferred to more frequent dosing schedules. For physicians, symptom control was five times as important as the risk of malignancy, whereas the relative importance between symptom control and risk of malignancy was about the same for patients. |
| Hazlewood, 2020 | (1) To quantify patient preferences for maintenance therapy in Crohn’s disease and (2) explore preference heterogeneity. | 155 CD patients | DCE survey | 6 attributes; (i) chance of remission, (ii) chance of having a side-effect that requires you to stop the medication, (iii) dosing of the medication (use of a daily oral pill, weekly or biweekly injection, intravenous infusion or a combination of infusion and pill), (iv) possible low blood counts or liver reaction, (v) need for a course of prednisone, (vi) small risk of serious infection and possible increased risk of certain cancers | In the DCE, maintenance of remission was the most important attribute. Across the range of levels considered, maintaining remission was 2.5 times more important than withdrawal due to an adverse event, the next most important attribute. Other attributes were less important. On average, patients were willing to accept a risk of infection/cancer, a course of prednisone, and possible low blood counts/liver reaction for a treatment that had an absolute increase in the chance of remission. |
| MacKenzie-Smith, 2018 | To investigate patient preferences for different drug formulations | 380 UC patients and 159 gastroenterologists | DCE survey | Not clear | The most important attributes for patients were appearance (44%), units per administration (39%) and administration per day (17%) |
| Byrne, 2014 | (1) To determine patient preferences for surgical intervention in UC prospectively and (2) to quantify the worth of living with a permanent stoma as opposed to a pouch, and (3) to compare patient preferences with those of surgeons and gastroenterologists | 55 UC patients, 91 surgeons and 78 gastroenterologists | Telephone interviews including the prospective measure of preference | NA | The preferences of the patients and gastroenterologists were not too dissimilar and they would rather choose to have escalating medical therapy than any operation in an acute setting. If surgery was unavoidable they were more willing to choose J-pouch surgery to avoid a permanent stoma. Their preferences were consistently different from those of colorectal surgeons in all the acute scenarios and the differences were statistically significant. |
| Gregor, 2018 | (1) To determine IBD patients’ willingness to pay for various treatment features, as well as (2) explore patients’ demographic and disease characteristics that may be associated with their willingness to pay. | 586 IBD patients | DCE survey (e.g., willingness to pay) | 12 attributes in 3 categories: 1) “administration characteristics” such as (i) pain during administration, (ii) dosing schedule, and (iii) mode of administration; 2) “efficacy outcomes” comprising (iv) symptom relief, (v) mucosal healing, (vi) speed of onset, and (vii) need for steroids; and 3) “safety risks” including (viii) injection reactions, (iv) time on market/number of patients exposed, (v) chance of surgery or (vi) hospitalization in the following year, (vii) and infusion reactions. | Reducing pain during administration, mucosal healing, reducing costs and symptom relief were the highest-ranking attributes. Conversely, infusion reactions and risk of hospitalization or surgery were the lowest-ranking attributes. |
| Hagelund, 2020 | (1) To explore HRQoL in a Danish population with UC and (2) to elicit patients’ preferences and expectations for advanced treatments, such as biologic treatments | 169 UC patients | DCE survey | 4 attributes; mode of administration, efficacy (significant improvement of symptoms), time to certainty about effect, and steroid use | The most important medical treatment attribute was efficacy within eight weeks. Additionally, respondents stated a preference for avoiding taking steroids, for fast onset of effect and for oral formulations. We saw that there was no difference in respondents’ preferences between taking medication as IV infusions every eight weeks and SC injections every four weeks. |
| van Deen, 2020 | To quantify the importance of different IBD-related symptoms and symptom-levels for IBD patients. | 8 IBD patients for the FGD and 129 IBD patients for the survey | 3xFGD and  Choice-based conjoint analysis (CBCA) survey | NA | 4 attributes; stool frequency, abdominal pain, blood in stools, and urgency  CBCA revealed that urgency was the most important symptom to patients, followed by abdominal pain and blood in stools. Urgency associated with incontinence received particularly high scores and was perceived to be more than 3x as important as urgency without incontinence, 1.4x as important as severe abdominal pain, 2.3x as important as severe rectal bleeding, and 4.6x as important as having 5 or more stools more than normal. |
| Byrne, 2007 | To investigate patients preferences for surgical or medical interventions and to compare these with the preferences of colorectal surgeons and gastroenterologists. | 41 CD patients, 92 colorectal surgeons and 74 gastroenterologists | Interviews including the prospective measure of preference | NA | Willingness to gamble (WTG) and willingness to trade (WTT) among patients were highest for total proctocolectomy with permanent stoma or restorative proctocolectomy. Patients also gambled and traded a greater amount of their remaining life expectancy than for any other scenarios to avoid these surgical options. Significantly more patients were prepared to gamble to avoid proctocolectomy, resulting in a permanent stoma compared with subtotal colectomy without a stoma. |
| Gray, 2009 | To examine the preferences of UC patients to understand better what they look for in a therapy when managing their disease and their satisfaction with current therapy | 100 UC patients | Allocation of points survey | Provides consistent relief, relieves rectal bleeding, prevents a flare of UC, reduces abdominal pain, provides fast relief, reduces the number bowel movements during the day, trust that it will work as promised, prevents diarrhea, reduces the urge to have a bowel movement, few side effects, convenient to take, easy to take, reduces the number of bowel movements at night, flexible dosing (modify when necessary), cost, number of times medication taken daily, number of pills per dose, available in different forms, taken once a day, on formulary | Patients rated the most important medication attributes as those related to efficacy (e.g. provides consistent relief, relieves rectal bleeding, prevents a flare, provides fast relief, etc.) and safety (e.g. few side effects), while other considerations (e.g. once-daily dosing, number of pills per dose, cost, etc.) were far less important. In pair-wise comparisons, speed of symptom relief was rated the most important medication attribute (preferred 84% of the time), followed by few side effects (preferred 74% of the time). |
| Bewtra, 2014 | (1) To quantify the tolerance of patients with UC for life-threatening serious adverse events (SAEs) in exchange for specific treatment benefits and (2) to elicit patients’ willingness to accept tradeoffs among therapeutic options regarding medical and surgical interventions for UC. | 293 UC patients | DCE survey | 5 attributes; disease activity, surgical outcomes, increased chance of dying from colorectal cancer within 10 yr, increased chance of dying from lymphoma within 10 yr, increased chance of dying from serious infection within 10 yr. | A desire to avoid surgery and the surgery type (ostomy versus J-pouch) influenced patients’ choices more than a specified range of 10-year mortality risks from lymphoma or infection, or disease activity (mild versus remission). To avoid an ostomy, patients were willing to accept a >5% 10-year risk of dying from lymphoma or infection from medical therapy, regardless of medication efficacy. However, data on patients’ stated choice indicated perceived equivalence between J-pouch surgery and incompletely effective medical therapy  Patients with UC are willing to accept relatively high risks of fatal complications from medical therapy to avoid a permanent ostomy and to achieve durable clinical remission. However, patients view J-pouch surgery, but not permanent ileostomy, as an acceptable therapy for refractory UC in which medical therapy is unable to induce a durable remission. |
| Hodgkins, 2012 | To understand differences in patient preferences for oral 5-ASA therapies in mild-to-moderate UC based on self-reported adherence. | (1) 14 UC patients and (2) 400 UC patients | (1) semi-structured interviews and (2)  DCE survey | NA | Six attributes were identified during the interviews for inclusion in the study: ease of swallowing, time of day when the medication must be taken/time of day, quantity needed per administration, extent of flare resolution, likelihood of flare occurrence within 12 months, and cost of therapy.  Patients have a stronger preference for clinical benefits (risk of symptom flares and treating your symptom flares) over other treatment attributes. |
| Arseneau, 2006 | To investigate how individual patient preferences for the possible outcomes of treatment with infliximab, cyclosporine, and total colectomy influence the optimal individual treatment choice for steroid- refractory ulcerative colitis. | 48 UC patients | Interviews: Utility weights were measured using the time trade-off method. A visual rating scale was also used to collect preferences data. | Remission, active UC, infusion reaction, hypertension, pneumonia, ileostomy, surgical complications, J pouch, misdiagnosed CD, obstruction, pouchitis, chronic pouchitis, stage III colorectal cancer, stage IV colorectal cancer | Utility weights were highest for remission, hypertension, J Pouch, Ileostomy, surgical complications and infusion reaction. |
| Kim, 2016 | To determine the preferences for anti-TNF agents (ADA and IFX) and identify the contributing factors for this preference | 189 CD patients | Survey | Easy to use, self-care, dislike of needles, frequency of administration, time of administration, place of administration, mode of administration, doctor’s presence, interference with everyday life, cost. | Patients attributed “doctor’s presence” as the most common reason to choose one option over the other followed by “place of administration”, “easy to use” and “interference with everyday life”. |
| Vavricka, 2012 | To evaluate which anti-TNF drug a patient would select given an appropriate level of medical information as well as the factors contributing to the specific preferences. | 100 CD patients | Survey | Application mode, scientific evidence for efficacy, time interval between application, time required for therapy, ease of use, side-effects, other patients recommendation, physician’s recommendations | The most important attributes were side effect profile (34%), physician’s recommendation (22%), the amount of available literature evidence supporting the efficacy of the specific medication (21%), route of administration (13%), recommendations by other IBD patients (5%), time required to get the specific treatment (4%), and interaction with other medications (1%). |
| Bewtra, 2020 | To quantify patients’ preferences for different treatment outcomes and adverse events. | 812 CD patients | DCE Survey | Severity and duration of symptoms, steroid duration, infection risk, cancer risk, surgery risk | Latent class analysis demonstrated 3 distinct groups of survey responders whose choices were strongly influenced by avoidance of active symptoms (61%), avoidance of corticosteroid use (25%), or avoidance of risks of cancer, infection or surgery (14%) when choosing a therapy. |
| Louis, 2020 | To create a descriptive framework that includes the most relevant attributes that influence patients’ decision making regarding IBD treatment. | 8 patient representatives and 9 gastroenterologists | FGD | NA | The descriptive framework included 10 attributes within three different domains (i) efficacy consisting out of abdominal pain, other disease-related pain (anal pain, joint pain/stiffness or eye pain), bowel urgency and fatigue, (ii) complications/risk consisting out of risk of cancer and serious infections within the next 10 years (excluding non-melanoma skin cancer), risk of mild to moderate complications (mild/moderate complications means nausea, vomiting, headache, non- serious infections, lab abnormalities, skin reactions, and infusion reactions) and aesthetic complications related to treatment (hair loss, unintentional weight change, facial hair, acne, puffy face), (iii) HRQoL/well-being consisting out of emotional status, sexual life, and social life and relationships (interpersonal reactions) |
| Casellas, 2016 | To identify factors associated with patient's satisfaction of UC management by a better understanding how patients and physicians perceive relevant aspects related to treatment of the disease | 22 UC patients and 20 clinicians | Deplhi study | NA | Important factors were: control bowel movements, eliminate rectal bleeding, promptly relief pain, achieve episode remission, prevent flare-ups, avoid the need of a colectomy, improve quality of life, reduce excess gas, reduce bloating, few short-term adverse effects, few long-term adverse effects, minimize number of medications, minimize number of doses, minimize number of pills, reduce pill size, be administered orally, avoid rectal route, make it compatible with patient’s daily life |
| Poulos, 2015 | To quantify patient preferences for multiple attributes of recovery following abdominal surgery | 387 IBD patients | DCE survey | Bowel related symptoms, number of additional hospital days due to delayed bowel recovery, indigestion, copayment for hospital stay | The respondents indicated a preference to avoid symptoms of delayed bowel recovery and indigestion, and to avoid additional hospital days and higher copayments for hospital stays. The preference weights for moderate severity were statistically different than the weights for mild and severe (P < 0.05), regardless of the number of additional hospital days, suggesting that respondents perceived differences between severity levels. The relative importance of improving symptoms associated with bowel recovery from moderate to mild or from severe to moderate increased with additional hospital days. An improvement in symptoms from moderate to mild symptoms with 2, 4, and 7 additional hospital days was 1.4, 1.8, and 2.4 times as important as improving symptoms from moderate to mild with no additional hospital days. The relative importance of improving symptoms from severe to moderate was 1.5 times as important as improving symptoms from moderate to mild, regardless of the number of additional hospital days. Mean WTP to avoid worsening symptoms from mild to moderate ranged from $93 (95% CI: $33-153) with no additional hospital days to $224 (95% CI: $80-367) with 7 additional hospital days. |
| Mahadev, 2011 | To determine which factors are considered by patients to be the most important and most unfavorable to their quality of life, and to gauge the relative impact of perianal and nonperianal symptoms. | 69 IBD patients | Willingness to pay and time trade-off survey | 16 QOL factors classified into 4 domains: *physical* (anal incontinence, anal pain, anal discharge, anal itch), *functional (activity restriction)* (physical, sexual, social, and work activity restriction; sleep interference; medical costs), *emotional* (emotional distress, loss of independence, confidence to go out, feeling unclean), and *cosmetic* (self-image, cosmetic appearance) | Anal pain or discomfort was the aspect most often rated as highly important (41% of participants). The importance of anal discharge, physical activity restriction, sleep interference, and feeling unclean were also rated to be high by a similar proportion (39%). Approximately one-third of patients rated the functional and emotional aspects of perianal disease to be of high importance. Least likely to be highly important were loss of independence, cosmetic appearance, and anal itch. Additional aspects nominated individually by patients included fatigue, bloating and indigestion, fecal soiling, and reliance on medication. |
| Hodgkins, 2010 | To determine if adherence behavior could be explained by differences in patient preferences for 5-ASA therapies | 400 UC patients | DCE survey | 6 attributes; ease of swallowing, number of administrations per day, number of pills per administration, symptom flare resolution, likelihood of flare occurrence, cost | Clinical effectiveness and a return to normal bowel functioning with mucosal healing was most highly valued by participants |
| Lichtenstein, 2010 | To quantify treatment preferences and priorities of patients with Crohn’s disease | 252 CD patients | DCE and SW survey | Proportion of patients with CD who respond to treatment within 2 weeks (rapid respons), proportion of patients with CD who are able to discontinue steroids within 1 y of treatment (discontinue steroids), proportion of patients with CD who achieve a lasting reduction in disease activity (lasting remission), how the medication would be taken (subcutaneous injection or intravenous injection, so the mode of administration), location where the medication would be taken, frequency that the medication must be taken, length of time the medication has been used to treat CD, number of patients worldwide who have been treated with the medication, number of different conditions for which the product has been approved by the US FDA | The three attributes of greatest importance in accounting for patients’ biologic therapy preference were (greater) proportion of patients achieving lasting remission, (decreased) frequency of medication administration, and (higher) proportion of patients responding in 2 weeks. In the direct ranking exercise, the proportion of CD patients achieving lasting remission was selected as the most important attribute by the most respondents (27%), followed by the proportion of CD patients responding in 2 weeks (23%). |

# 1B) Overview of treatment characteristics assessed in previous patient preference studies for inflammatory bowel disease

| Attribute (group) name | Number of patient preference studies of 22 studies investigating attributes (group) |
| --- | --- |
| Mechanism of action | 1 |
| Mode/route of administration | 10 |
| Frequency of administration/dosing schedule | 12 |
| Units per administration/number of pills per dose | 5 |
| Place of administration/where the medication would be taken | 2 |
| Ease of use/swallowing | 5 |
| Trust that it will work as promised | 2 |
| Pain during administration | 1 |
| Time on market/number of patients exposed | 2 |
| On formulary | 2 |
| Scientific evidence for efficacy | 1 |
| Interference with everyday life | 2 |
| Time required for therapy | 1 |
| Medical costs | 7 |
| Long-term remission | 4 |
| Chance of remission/efficacy | 2 |
| Short-term improvement | 4 |
| Time until symptoms improve/speed of onset | 4 |
| Symptom relief | 2 |
| Severity and duration of symptoms | 1 |
| Prevents a flare-up or likelihood of occurrence | 4 |
| Few short-term adverse effects | 1 |
| Few long-term adverse effects | 1 |
| Disease activity | 1 |
| Need for steroids | 6 |
| Mucosal healing | 1 |
| Tolerability of side effects (chance of stopping the medication because of side-effects) | 2 |
| Side-effects | 2 |
| Fatigue | 2 |
| Rash | 1 |
| Acne | 1 |
| Skin reactions | 1 |
| Hair loss | 1 |
| Facial hair | 1 |
| Weight change | 1 |
| Puffy face | 1 |
| Stool frequency | 1 |
| Diarrhea | 1 |
| Abdominal pain | 3 |
| Anal pain or discomfort | 3 |
| Joint pain | 1 |
| Eye pain | 1 |
| Blood in stools | 1 |
| Rectal bleeding | 2 |
| Obstruction | 1 |
| Urgency with/without continence | 3 |
| Indigestion | 1 |
| Pouchitis | 1 |
| Bowel movements or bowel related symptoms | 3 |
| Excessive gas | 1 |
| Bloating | 1 |
| Nausea | 1 |
| Vomiting | 1 |
| Hypertension | 1 |
| Pneumonia | 1 |
| Possible low blood counts or liver reaction | 1 |
| Lab abnormalities | 1 |
| Risk of serious infection | 6 |
| Risk of lymphoma | 2 |
| Risk of colorectal cancer | 2 |
| Risk of malignancy/certain cancers | 4 |
| Injection reactions | 1 |
| Infusion reactions | 3 |
| Chance of hospitalization | 2 |
| Risk of surgery | 6 |
| Surgical complications | 1 |
| Avoid a permanent stoma | 3 |
| Self-care or independence | 2 |
| Emotional status or distress | 2 |
| Sexual life/activity restriction | 2 |
| Social life and relationships/activity restriction | 2 |
| Physical activity restriction | 1 |
| Work activity restriction | 1 |
| Sleep interference | 1 |
| Improve quality of life | 2 |

# 2A - Overview of inflammatory bowel disease medical products assessed by European Medicines Agency or Federal Agency for Medicines and Health Products

Data extracted in March 2021 from European Assessment Reports of products approved for treatment of Inflammatory Bowel Disease from <https://www.ema.europa.eu/en/medicines>. When the products were not found on the EMA site, but prescribed by clinicians in Belgium for Inflammatory Bowel Disease and/or indicated on <https://www.bcfi.be/nl/chapters/4?frag=2485>, data was extracted from the products’ leaflet (<https://www.fagg.be/nl>).

|  | **Product name** | **Active substance** | **CD and/or UC** | **Benefit/favorable effects (effect measure and size)** | **Risks/unfavourable effects** | **Comments** |
| --- | --- | --- | --- | --- | --- | --- |
| 1 | Entyvio | Vedolizumab | CD and UC | UC; symptom improvement: 47% of patients vs 26% of patients receiving placebo + maintained the effect up to 52 weeks more effectively than placebo  CD; symptom improvement: 15% of patients vs 7% of patients receiving placebo + maintained the effect up to 52 weeks more effectively than placebo | Nasopharyngitis (inflammation of the nose and throat such as a cold), headache, arthralgia (joint pain) | EMA |
| 2 | Stelara | Ustekinumab | CD and UC | UC; symptoms (almost) gone: 16% of patients vs 5% of patients receiving placebo  CD; symptom score improved: STUDY 1; 34% of patients vs 21% of patients receiving placebo  STUDY 2; 56% of patients vs 29% of patients receiving placebo | Nasopharyngitis (inflammation of the nose and throat such as a cold), headache, hypersensitivity (allergic reaction) | EMA |
| 3 | Xeljanz | Tofacitinib | UC | Reducing symptoms: more effective in 3 studies vs placebo | Headache, infection and inflammation of the nose and throat, diarrhea, nausea, joint pain, hypertension, pneumonia, cellulitis, herpes zoster, urinary tract infection, diverticulitis, appendicitis, opportunistic infections | EMA |
| 4 | Remicade | Infliximab | CD and UC | UC; reducing symptoms; more effective vs placebo  CD; greater improvement of symptoms + fistulae healing + increased time that patients continued to respond to treatment vs placebo | Viral infections, headache, upper respiratory tract infection (colds), sinusitis, nausea, abdominal pain, infusion-related reactions, pain | EMA |
| 5 | Flixabi | Infliximab | CD and UC | Generic medicine, see Remicade | Generic medicine, see Remicade | EMA |
| 6 | Remsima | Infliximab | CD and UC | Generic medicine, see Remicade | Generic medicine, see Remicade | EMA |
| 7 | Inflectra | Infliximab | CD and UC | Generic medicine, see Remicade | Generic medicine, see Remicade | EMA |
| 8 | Zessly | Infliximab | CD and UC | Generic medicine, see Remicade | Generic medicine, see Remicade | EMA |
| 9 | Simponi | Golimumab | UC | Response to treatment; 51% of patients vs 30% of patients receiving placebo | Upper respiratory tract infection (nose, throat or voice box), sepsis, pneumonia, tuberculosis and infections due to fungi or yeasts, demyelinating disorders, re-activation of hepatitis B, congestive heart failure, lupus-like syndrome, blood reactions, severe allergic reactions, vasculitis, lymphoma, leukaemia | EMA |
| 10 | Humira | Adalimumab | CD and UC | UC; improvement in symptoms + healing of the mucosa vs placebo  CD; improvement in symptoms; more effective vs placebo | Infections (including in the nose, throat and sinuses), injection site reactions (redness, itching, bleeding, pain or swelling), headache, muscle and bone pain, serious infections, blood cancers, failure of bone marrow to produce blood cells, nerve damage, lupus and lupus-like conditions, Stevens-Johnson syndrome | EMA |
| 11 | Idacio | Adalimumab | CD and UC | Generic medicine, see Humira | Generic medicine, see Humira | EMA |
| 12 | Amsparity | Adalimumab | CD and UC | Generic medicine, see Humira | Generic medicine, see Humira | EMA |
| 13 | Imraldi | Adalimumab | CD and UC | Generic medicine, see Humira | Generic medicine, see Humira | EMA |
| 14 | Hefiya | Adalimumab | CD and UC | Generic medicine, see Humira | Generic medicine, see Humira | EMA |
| 15 | Amgevita | Adalimumab | CD and UC | Generic medicine, see Humira | Generic medicine, see Humira | EMA |
| 16 | Yuflyma | Adalimumab | CD and UC | Generic medicine, see Humira | Generic medicine, see Humira | EMA |
| 17 | Hyrimoz | Adalimumab | CD and UC | Generic medicine, see Humira | Generic medicine, see Humira | EMA |
| 18 | Hulio | Adalimumab | CD and UC | Generic medicine, see Humira | Generic medicine, see Humira | EMA |
| 19 | Cimzia | Certolizumab pegol | CD and UC | Nine main studies found Cimzia effective for reducing symptoms or response to therapy or sustained remission after 52 weeks of inflammatory conditions. Trials included adults with active rheumatoid arthritis, axial spondyloarthritis, psoriatic arthritis and moderate to severe plaque psoriasis | Bacterial infections including abscesses, viral infections, eosinophilic disorders, leucopenia, nausea, headaches, sensory abnormalities, high blood pressure, hepatitis including increased levels of liver enzymes, rash, fever, pain, weakness, itching and reactions at the injection site | Prescribed by clinicians. Not authorized by EMA for IBD + not mentioned on BCFI as treatment for IBD.  🡪 Source: EMA for other indication |
| 20 | Rinvoq | Upadacitinib | CU | Low disease activity or reduction of symptoms in 5 studies with rheumatoid arthritis vs placebo | Upper respiratory tract infections (nose and throat infections), serious infections | Not authorized by EMA for CU. BCFI: can be used for patients with UC  🡪 Source: EMA for other indication |
| 21 | Salazopyrine | Sulfasalazine | CD and CU | Not available | Leukopenia, thrombocytopenia, loss of appetite, depression, dizziness, headache, taste abnormalities, tinnitus, cough, dyspnea, nausea, stomach problems, abdominal pain, vomiting, diarrhea, jaundice, purpura, pruritus, alopecia, urticaria, toxic epidermal necrolysis (Lyell’s syndrome), Stevens-Johnson syndrome, arthralgia, proteinuria, fever, elevated liver enzymes, facial edema | Not found on the EMA site. BCFI: can be used for both UC and CD. Side-effects obtained from the leaflet |
| 22 | Claversal | Mesalazine | CD and CU | Not available | Bloated belly, anal discomfort, irritation at the administration site, painful rectal tenesmus, headache, nausea, neuropathy, abdominal pain, diarrhea, flatulence, vomiting, loss of appetite, increased amylase, dizziness, acute pancreatitis, skin rash (urticaria and erythema), increased skin sensitivity (photosensitivity), alopecia, myocardial and pericardial inflammation, hypersensitivity reactions such as allergic exanthema, drug fever, lupus erythematosus syndrome, pancolitis, changes in liver function parameters, bilirubin, cirrhosis, liver failure, hepatitis, cholestatic hepatitis, impairment of kidney function (interstitial nephritis, renal insufficiency), allergic and fibrotic lung reactions including shortness of breath, coughing, tracheal spasms, inflammation of the alveoli, pulmonary eosinophilia and lung infiltration, pneumonitis, muscle pain, joint pain, blood count abnormalities including (aplastic) anemia, leukopenia, thrombocytopenia, neutropenia, agranulocytosis, pancytopenia, oligospermia | Not found on the EMA site. BCFI: can be used for both UC and CD. Side-effects obtained from the leaflet |
| 23 | Colitofalk | Mesalazine | CD and CU | Not available | Blood count abnormalities including aplastic anemia, leukopenia, thrombocytopenia, neutropenia, agranulocytosis, pancytopenia, hypersensitivity reactions such as allergic exanthema, drug fever, lupus erythematosus syndrome, pancolitis, headache, dizziness, neuropathy, myocarditis, pericarditis, allergic and fibrotic lung reactions including shortness of breath, coughing, tracheal spasms, inflammation of the alveoli, pulmonary eosinophilia and lung infiltration, pneumonitis, abdominal pain, diarrhea, dyspepsia, flatulence, nausea, vomiting, acute pancreatitis, cholestatic hepatitis, hepatitis, increased skin sensitivity (photosensitivity), alopecia, arthralgia, myalgia, impairment of kidney function (interstitial nephritis, renal insufficiency), oligospermia, asthenia, fatigue, changes in liver function parameters, abnormalities in pancreatic enzymes | Not found on the EMA site. BCFI: can be used for both UC and CD. Side-effects obtained from the leaflet |
| 24 | Mesalazine Teva | Mesalazine | CD and CU | Not available | Blood count abnormalities including aplastic anemia, leukopenia, thrombocytopenia, neutropenia, agranulocytosis, pancytopenia, headache, dizziness, neuropathy, myocarditis, pericarditis, allergic and fibrotic lung reactions including shortness of breath, coughing, tracheal spasms/bronchospasms, inflammation of the alveoli, pulmonary eosinophilia and lung infiltration, abdominal pain, diarrhea, dyspepsia, flatulence, nausea, acute pancreatitis, impairment of kidney function (interstitial nephritis, renal insufficiency), increased skin sensitivity (photosensitivity), alopecia, myalgia, arthralgia | Not found on the EMA site. BCFI: can be used for both UC and CD. Side-effects obtained from the leaflet |
| 25 | Mezavant | Mesalazine | CD and CU | Not available | Thrombocytopenia, agranulocytosis, facial edema, headache, dizziness, somnolence, tremor, earache, tachycardia, hypertension, hypotension, pharyngolaryngeal pain, abdominal distension, abdominal pain, colitis, diarrhea, dyspepsia, vomiting, flatulence, nausea, pancreatitis, rectal polyp, abnormal liver function test, pruritus, rash, acne, alopecia, urticaria, increased skin sensitivity (photosensitivity), arthralgia, back pain, myalgia, kidney failure, asthenia, fatigue, pyrexia | Not found on the EMA site. BCFI: can be used for both UC and CD. Side-effects obtained from the leaflet |
| 26 | Pentasa | Mesalazine | CD and CU | Not available | Blood disorders such as (aplastic) anemia, agranulocytosis, neutropenia, leukopenia, pancytopenia, thrombocytopenia, and eosinophilia, hypersensitivity reaction including allergic exanthema, anaphylactic reaction, and drug fever, headache, dizziness, neuropathy, myocarditis, pericarditis, allergic alveolitis, allergic and fibrotic lung reactions including shortness of breath, coughing, tracheal spasms/bronchospasms, pulmonary eosinophilia, interstitial lung disease, pulmonal infiltration, pneumonitis, pleuropericarditis, pancolitis, diarrhea, abdominal pain, nausea, vomiting, flatulence, increased amylase, acute pancreatitis, elevated liver enzymes, cholestase parameters and bilirubin, hepatoxicity (hepatitis, cirrhose, liver failure), skin rash, increased skin sensitivity (photosensitivity), alopecia, myalgia, arthralgia, abnormal liver function (acute and chronic interstitial nephritis, nephrotic syndrome, renal insuffiency), discoloration of the urine, oligospermia | Not found on the EMA site. BCFI: can be used for both UC and CD. Side-effects obtained from the leaflet |
| 27 | Azathioprine Sandoz | Azathioprine | CD and CU | Not available | Viral, bacterial and fungal infections, infections associated with neutropenia, progressive multifocal leuko-encephalopathy (associated with JC-virus), neoplasms including lymphoproliferative disorders, skin cancers (melanomas and non-melanomas), sarcomas (Kaposi and non-Kaposi sarcomas) and "in situ" cervical cancer, acute myeloid leukemia and myelodysplastic syndrome, bone marrow depression, leukopenia, thrombocytopenia, anemia, agranulocytosis, pancytopenia, aplastic anemia, megaloblastic anemia, erythroid hypoplasia, hypersensitivity reactions, Stevens-Johnson syndrome and toxic epidermal necrolysis, pneumonitis, nausea, pancreatitis, diarrhea, cholestasis, life-threating liver damage, abnormal liver function test, alopecia | Not found on the EMA site. BCFI: can be used for both UC and CD. Side-effects obtained from the leaflet |
| 28 | Imuran | Azathioprine | CD and CU | Not available | Viral, bacterial and fungal infections, progressive multifocal leuko-encephalopathy (associated with JC-virus), neoplasms including lymphoproliferative disorders, skin cancers (melanomas and non-melanomas), sarcomas (Kaposi and non-Kaposi sarcomas) and "in situ" cervical cancer, acute myeloid leukemia and myelodysplastic syndrome, bone marrow depression, leukopenia, thrombocytopenia, anemia, agranulocytosis, pancytopenia, aplastic anemia, megaloblastic anemia, erythroid hypoplasia, hypersensitivity reactions, Stevens-Johnson syndrome and toxic epidermal necrolysis, pneumonitis, nausea, pancreatitis, diarrhea, cholestasis, life-threating liver damage, abnormal liver function test, alopecia | Not found on the EMA site. BCFI: can be used for both UC and CD. Side-effects obtained from the leaflet |
| 29 | Puri-Nethol | Mercaptopurine | CD and CU | Not available | Viral and bacterial infections, infections associated with neutropenia, neoplasms including lymphoproliferative disorders, skin cancers (melanomas and non-melanomas), sarcomas (Kaposi and non-Kaposi sarcomas) and "in situ" cervical cancer, bone marrow suppression, leukopenia, thrombocytopenia, anemia, hypersensitivity reactions, anorexia, nausea, vomiting, pancreatitis, intestinal ulcerations, cholestasis, liver toxicity, liver necrosis, alopecia, oligospermia | Not found on the EMA site. BCFI: can be used for both UC and CD. Side-effects obtained from the leaflet |
| 30 | Emthexate | Methotrexaat | CD | Not available | Sepsis, lymphoma, suppression of hematopoiesis (anemia and thrombocytopenia), aplastic anemia, lymphoproliferative disorders, anaphylactic and anaphylactoid reactions, opportunistic infections, hypogammaglobulinemia, diabetes, mood swings, temporary cognitive dysfunction, paresthesia, hemiparesis, (leuko-) encephalopathy, convulsions, headache, paresis, dysarthria and aphysia, somnolence, paresthesia, hypoaesthesia, blurred vision, severe change in vision, temporary blindness or vision impairment, conjunctivitis, hypotension, pericarditis, pericardial effusion, thromboembolic events, vasculitis, pneumonia, pleural effusion, respiratory fibrosis, pharyngitis, chronic obstructive bronchopneumopathy, stomatitis, loss of appetite, vomiting, diarrhea, pancreatitis, ulceration and gastrointestinal bleeding, melena, enteritis, gingivitis, hematemesis, elevated liver enzymes, hepatitis, chronic fibrosis and cirrhosis, hepatoxicity, decrease in serum albumin, toxic epidermal necrolysis (Lyell’s syndrome), Stevens-Johnson syndrome, alopecia, erythema multiforma, erythematous rash, painful erosion of psoriasis plaques, photosensitivity, urticaria, acne, ecchymoses, changes in skin pigmentation, pruritus, dermatitis, ulceration and necrosis of the skin, joint pain/muscle pain, osteoporosis, stress fractures, renal insuffiency, nephropathy, dysuria, congenital abnormalities, abortion, menstrual disorder, disorders of ovogenesis/spermatogenesis, impotence, infertility, loss of libido, temporary oligospermia, vaginal discharge, gynecomastia, lumps/nodes | Not found on the EMA site. BCFI: can be used for CD. Side-effects obtained from the leaflet |
| 31 | Ledertrexate | Methotrexaat | CD | Not available | Sepsis, lymphoma, tumorlysissyndrome, suppression of hematopoiesis (anemia and thrombocytopenia), aplastic anemia, lymphoproliferative disorders, anaphylactic and anaphylactoid reactions, opportunistic infections, hypogammaglobulinemia, diabetes, mood swings, temporary cognitive dysfunction, hemiparesis, (leuko-) encephalopathy, convulsions, headache, paresis, dysarthria, somnolence, paresthesia, hypoaesthesia, blurred vision, severe change in vision, temporary blindness or vision impairment, conjunctivitis, hypotension, pericarditis, pericardial effusion, thromboembolic events, vasculitis, pneumonia, pleural effusion, respiratory fibrosis, pharyngitis, chronic obstructive bronchopneumopathy, pancreatitis, loss of appetite, vomiting, diarrhea, stomatitis, ulceration and gastrointestinal bleeding, melena, enteritis, gingivitis, hematemesis, elevated liver enzymes, chronic fibrosis and cirrhosis, acute pancreatitis, hepatoxicity, decrease in serum albumin, toxic epidermal necrolysis (Lyell’s syndrome), Stevens-Johnson syndrome, alopecia, erythema multiforma, erythematous rash, painful erosion of psoriasis plaques, photosensitivity, urticaria, acne, ecchymoses, changes in skin pigmentation, pruritus, ulceration and necrosis of the skin, joint pain/muscle pain, osteoporosis, stress fractures, renal insuffiency, nephropathy, dysuria, congenital abnormalities, abortion, menstrual disorder, disorders of ovogenesis/spermatogenesis, impotence, infertility, loss of libido, temporary oligospermia, vaginal discharge, gynecomastia, lumps/nodes | Not found on the EMA site. BCFI: can be used for CD. Side-effects obtained from the leaflet |
| 32 | Metoject | Methotrexaat | CD | Not available | Faryngitis, infections, sepsis, conjunctivitis, lymphoma, leukopenia, anemia, thrombopenia, pancytopenia, agranulocytosis, bone marrow suppression, lymphoproliferative disorders, allergic reactions, eosinophilia, anaphylactic shock, hypogammaglobulinemia, diabetes, depression, mood swings, confusion, headache, fatigue, drowsiness, dizziness, pain, muscle asthenia or paresthesia/hypesthesia, change in sense of taste, convulsions, meningism, acute aseptic meningitis, paralysis, encephalopathy, vision disorders, impaired vision, retinopathy, pericarditis, pericardial effusions, pericardial tamponade, hypotension, thromboembolic phenomena, pneumonia, interstitial alveolitis/pneumonitis, pulmonary fibrosis, pneumocystis jirovecii pneumonia, shortness of breath and asthmatic bronchitis, pleural effusion, stomatitis, dyspepsia, nausea, loss of appetite, abdominal pain, diarrhea, ulcera, enteritis, vomiting, pancreatitis, gingivitis, abnormal liver function tests, cirrhosis, fibrosis, decreased serum albumin, acute hepatitis, liver failure, exanthema, erythema, pruritus, photosensitization, hair loss, increase in rheumatoid nodules, skin ulcers, herpes zoster, vasculitis, herpetiform eruptions of the skin, urticaria, increased pigmentation, acne, petechiae, ecchymosis, allergic vasculitis, toxic epidermal necrolysis (Lyell’s syndrome), Stevens-Johnson syndrome, arthralgia, myalgia, osteoporosis, stress fracture, osteonecrosis, inflammation and ulceration of the urinary bladder, renal dysfunction, impaired micturition, renal failure, oliguria, anuria, electrolyte disturbances, proteinuria, inflammation and ulceration of the vagina, loss of libido, impotence, gynecomastia, oligospermia, menstrual disorder, vaginal discharge, fever, wound healing disorder | Not found on the EMA site. BCFI: can be used for CD. Side-effects obtained from the leaflet |
| 33 | (Neoral)-Sandimmun | Ciclosporine | CD and UC | Not available | Leukopenia, trombocytopenia, anemia, hemolytic uremic syndrome, microangiopathic hemolytic anemia, thrombotic thrombocytopenic purpura, thrombotic microangiopathy, hyperlipidemia, hyperglycemia, anorexia, hyperuricemia, hyperkalemia, hypomagnesemia, tremor, headache, convulsions, paresthesia, encephalopathy, motor polyneuropathy, migraine, hypertension, flushing, nausea, vomiting, abdominal discomfort, abdominal pain, diarrhea,  gingival hyperplasia, peptic ulcer, pancreatitis, abnormal liver function, hepatoxicity, hirsutism, acne, hypertrichosis, allergic rash, myalgia, muscle cramps, muscle weakness, myopathy, pain in lower extremities, impaired kidney function, menstrual disorders, gynecomastia, pyrexia, fatigue, edema, weight gain | Not found on the EMA site. BCFI: can be used for CD and UC. Side-effects obtained from the leaflet |
| 34 | Adoport | Tacrolimus | CD and UC | Not available | Increased susceptibility to infections (Viral, bacterial and fungal), progressive multifocal leuko-encephalopathy (associated with JC-virus), nephropathy associated with BK-virus, activation of latent infections, increased risk for developing malignancies, anemia, leukopenia, thrombocytopenia, leukocytosis, abnormal red blood cell analysis results, coagulopathies, abnormal results of coagulation and bleeding tests, pancytopenia, neutropenia, thrombotic thrombocytopenic purpura, hypoprothrombinemia, thrombotic microangiopathy, anaphylactic and anaphylactoid reactions, hirsutisme, hyperglycemic disorders, diabetes mellitus, hyperkalemia, hypomagnesemia, hypophosphatemia, hypokalemia, hypocalcemia, hyponatremia, fluid overload, hyperuricemia, decreased appetite, metabolic acidosis, hyperlipidemia, hypercholesterolemia, hypertriglyceridemia, other electrolyte imbalances, dehydration, hypoproteinemia, hyperphosphatemia, hypoglycemia, insomnia, symptoms of anxiety, confusion and disorientation, depression, depressed mood, mood disorders and mood swings, nightmares, hallucinations, mental disorders, psychotic disorders, tremor, headache, seizures, disorders of consciousness, paresthesia and dysesthesia, peripheral neuropathies, dizziness, decreased ability to write, nervous system disorders, coma, central nervous system hemorrhage and cerebrovascular accident, paralysis and paresis, encephalopathy, speech and language disorders, amnesia, hypertonia, myasthenia, blurred vision, photophobia, eye disorders, cataract, blindness, tinnitus, hypoacusis, neurosensory hearing loss, deafness, ischemic coronary disease, tachycardia, ventricular arrhythmia and cardiac arrest, heart failure, cardiomyopathy, ventricular hypertrophy, supraventricular arrhythmia, palpitations, pericardial effusion, torsade de pointes, hypertension, hemorrhage, thromboembolic and ischemic events, peripheral arterial disease, hypotensive arterial disease, infarct, deep venous thrombose, shock, dyspnea, parenchymal lung dysfunction, pleural effusion, pharyngitis, cough, nasal congestion, inflammation, respiratory failure, respiratory disorder, asthma, acute respiratory distress syndrome, diarrhea, nausea, inflammatory reactions of the gastrointestinal system, gastrointestinal ulceration and perforation, gastrointestinal bleeding, stomatitis and ulceration, ascites, vomiting, gastrointestinal and abdominal pain, complaints and symptoms of dyspepsia, constipation, flatulence, distended and distended abdomen, thin stools, gastrointestinal complaints and symptoms, paralytic ileus, acute and chronic pancreatitis, gastroesophageal reflux disease, impaired gastric emptying, subileus, pseudocysts in the pancreas, cholestasis, jaundice, hepatocellular damage, hepatitis, cholangitis, , thrombosis of the hepatic artery, vena-occlusive liver disease, liver failure, bile duct stenose, pruritus, rash, alopecia, acne, increased perspiration, dermatitis, increased photosensitivity, toxic epidermal necrolysis (Lyell’s syndrome), Stevens-Johnson syndrome, arthralgia, muscle spasms, limb pain, back pain, joint disorders, decreased mobility, renal function disorders, renal failure, acute renal failure, oliguria, tubular necrosis, toxic nephropathy, urinary tract abnormalities, symptoms of the bladder and urethra, anuria, hemolytic-uremic syndrome, nephropathy, hemorrhagic cystitis, dysmenorrhea and uterine bleeding, asthenic disorder, febrile disorder, edema, pain and discomfort, disturbed perception of body temperature, multi-organ failure, flu-like illness, temperature intolerance, pressing sensation in the chest, feeling nervous, thirst, falling, sensation of a pressing feeling in the chest, ulcers, increase in adipose tissue, feeling unusual, abnormalities in liver enzymes and liver function, increased alkaline phosphatase in the blood, weight gain, elevated amylase, abnormal ECG result, abnormal pulse and heart rate, weight loss, elevated blood lactate dehydrogenase, abnormal echocardiogram, prolonged QT interval on electrocardiogram | Prescribed by clinicians. Not authorized by EMA for IBD + not mentioned on BCFI as treatment for IBD.  Side-effects obtained from the leaflet |
| 35 | Advagraf | Tacrolimus | CD and UC | Advagraf showed effective as comparator medicine. After one year, 14% of the patients receiving Advagraf had experienced organ failure vs 15% with Prograft and 17% with Ciclosporin | Tremor, headache, nausea, diarrhoea, kidney problems, hyperglycemia, diabetes, hyperkalemia, hypertension and insomnia | Prescribed by clinicians. Not authorized by EMA for IBD + not mentioned on BCFI as treatment for IBD.  🡪 Source: EMA for other indication |
| 36 | Dailiport | Tacrolimus | CD and UC | Not available | Hirsutism, anemia, leukopenia, thrombocytopenia, leukocytosis, abnormal red blood cell analysis results, coagulopathies, abnormal results of coagulation and bleeding tests, pancytopenia, neutropenia, thrombotic thrombocytopenic purpura, hypoprothrombinemia, thrombotic microangiopathy, hyperglycemic disorders, diabetes mellitus, hyperkalemia, metabolic acidosis, other electrolyte imbalances, hyponatremia, hypervolemia, hyperuricemia, hypomagnesemia, hypokalemia, hypocalcemia, decreased appetite, hypercholesterolemia, hyperlipidemia, hypertriglyceridemia, hypophosphatemia, dehydration, hypoglycemia, hypoproteinemia, hyperphosphatemia, insomnia, symptoms of anxiety, confusion and disorientation, depression, depressed mood, mood disorders and mood swings, nightmares, hallucinations, mental illness, psychotic disorders, tremor, headache, nervous system disorders, convulsions, impaired consciousness, peripheral neuropathy, dizziness, sensibility and feeling disorders, impaired ability to write, encephalopathy, cerebral hemorrhages and strokes, coma, speech and language disorders, paralysis and paresis, amnesia, hypertonia,myasthenia, eye diseases, blurred vision, photophobia, cataract, blindness, tinnitus, (neurosensory) hearing loss, deafness, ischemic coronary artery disease, tachycardia, heart failure, ventricular arrhythmia and cardiac arrest, supraventricular arrhythmia, cardiomyopathy, ventricular hypertrophy, palpitations, pericardial effusion, Torsades de Pointes, hypertension, thromboembolic and ischemic events, vascular hypotensive disorders, bleeding, peripheral vascular disorders, deep vein thrombosis in the leg, shock, infarction, parenchymal lung abnormalities, dyspnea, pleural effusion, cough, pharyngitis, nasal congestion and inflammation, respiratory disorders, respiratory pathway disorders, asthma, acute respiratory distress syndrome, diarrhea, nausea, gastrointestinal complains, vomiting, gastrointestinal and abdominal pain, inflammatory reactions of the gastrointestinal system, gastrointestinal bleeding, gastrointestinal ulceration and perforation, ascites, stomatitis and ulceration, constipation, signs and symptoms of poor digestion, flatulence, swelling and enlargement, soft stools, acute and chronic pancreatitis, adynamic ileus, gastroesophageal reflux disease, impaired gastric emptying, pseudocysts in the pancreas, incomplete ileus, bile duct disorders, hepatocellular damage and hepatitis, bile obstruction, jaundice, vena-occlusive liver disease, thrombosis of the hepatic artery, liver failure, rash, pruritus, alopecia, acne, increasing sweating, dermatitis, photosensitivity, toxic epidermal necrolysis (Lyell’s syndrome), Stevens-Johnson syndrome, joint pain, back pain, muscle spasms, pain in extremity, joint complaints, decreased mobility, renal function disorders, renal failure, acute renal failure, toxic nephropathy, tubular necrosis, problems with urination, oliguria, bladder and urethra symptoms, hemolytic uremic syndrome, anuria, nephropathy, hemorrhagic cystitis, painful menstruation, uterine bleeding, fever, pain and discomfort, asthenia, edema, disturbed sensation of body temperature, flu-like symptoms, nervous feeling, abnormal sensation, multi-organ failure, sensation of a pressing feeling in the chest, temperature intolerance, falling, ulcers, chest tightness, thirst, increase in adipose tissue, abnormal results liver function test, increased alkaline phosphatase in the blood, weight gain, elevated amylase, abnormal ECG, abnormal pulse and heart rate, weight loss, elevated blood lactate dehydrogenase, abnormal echocardiogram, prolonged QT on electrocardiogram, increased susceptibility to infections (viral, bacterial and fungal), progressive multifocal leuko-encephalopathy (associated with JC-virus), nephropathy associated with BK-virus, activation of latent infections, increased risk for developing malignancies (both benign and malign neoplasm), allergic and anaphylactic reactions | Prescribed by clinicians. Not authorized by EMA for IBD + not mentioned on BCFI as treatment for IBD.  Side-effects obtained from the leaflet |
| 37 | Prograft | Tacrolimus | CD and UC | Not available | Increased susceptibility for infections (viral, bacterial and fungal), progressive multifocal leuko-encephalopathy (associated with JC-virus), nephropathy associated with BK virus, activation of latent infections, increased risk for developing malignancies, anemia, leukopenia, thrombocytopenia, leukocytosis, abnormal red blood cell analysis results, coagulopathies, abnormal results of coagulation and bleeding tests, pancytopenia, neutropenia, thrombotic thrombocytopenic purpura, hypoprothrombinemia, thrombotic microangiopathy, anaphylactic and anaphylactoid reactions, hirsutisme, hyperglycemic disorders, diabetes mellitus, hyperkalemia, hypomagnesemia, hypophosphatemia, hypokalemia, hypocalcemia, hyponatremia, fluid overload, hyperuricemia, decreased appetite, metabolic acidosis, hyperlipidemia, hypercholesterolemia, hypertriglyceridemia, other electrolyte imbalances, dehydration, hypoproteinemia, hyperphosphatemia, hypoglycemia, insomnia, symptoms of anxiety, confusion and disorientation, depression, depressed mood, mood disorders and mood swings, nightmares, hallucinations, mental illness, psychotic disorders, tremor, headache, seizures/convulsions, impaired consciousness, paresthesia and dysesthesia, peripheral neuropathies, dizziness, decreased ability to write, nervous system disorders, coma, central nervous system hemorrhage and cerebrovascular accident/stroke, paralysis and paresis, encephalopathy, speech and language disorders, amnesia, hypertonia, myasthenia, blurred vision, photophobia, eye disorders, cataract, blindness, tinnitus, hypoacusis/hearing loss, neurosensory hearing loss, deafness, ischemic coronary disease, tachycardia, ventricular arrhythmia and cardiac arrest, heart failure, cardiomyopathy, ventricular hypertrophy, supraventricular arrhythmia, palpitations, pericardial effusion, torsade de pointes, hypertension, hemorrhage, thromboembolic and ischemic events, peripheral arterial disease, hypotensive arterial disease, infarct, deep venous thrombose, shock, dyspnea, parenchymal lung dysfunction, pleural effusion, pharyngitis, cough, nasal congestion, inflammation, respiratory failure, respiratory disorder, asthma, acute respiratory distress syndrome, diarrhea, nausea, inflammatory reactions of the gastrointestinal system, gastrointestinal ulceration and perforation, gastrointestinal bleeding, stomatitis and ulceration, ascites, vomiting, gastrointestinal and abdominal pain, complaints and symptoms of dyspepsia, constipation, flatulence, distended abdomen, thin stools, gastrointestinal complaints and symptoms, paralytic ileus, acute and chronic pancreatitis, gastroesophageal reflux disease, impaired gastric emptying, subileus, pseudocysts in the pancreas, cholestasis, jaundice, hepatocellular damage, hepatitis, cholangitis, thrombosis of the hepatic artery, vena-occlusive liver disease, liver failure, bile duct stenose, pruritus, rash, alopecia, acne, increased perspiration, dermatitis, increased photosensitivity, toxic epidermal necrolysis (Lyell’s syndrome), Stevens-Johnson syndrome, arthralgia, muscle spasms, limb pain, back pain, joint disorders, decreased mobility, renal function disorders, renal failure, acute renal failure, oliguria, tubular necrosis, toxic nephropathy, urinary tract abnormalities, bladder and urethra related symptoms, anuria, hemolytic-uremic syndrome, nephropathy, hemorrhagic cystitis, dysmenorrhea and uterine bleeding, asthenic disorder, fever, edema, pain and discomfort, disturbed perception of body temperature, multi-organ failure, flu-like illness, temperature intolerance, pressing sensation in the chest, feeling nervous, feeling unusual, thirsty, chest tightness, ulcers, increase in adipose tissue, falling, abnormalities in liver enzymes and liver function, increased alkaline phosphatase in the blood, weight gain, elevated amylase, abnormal ECG result, abnormal pulse and heart rate, weight loss, elevated blood lactate dehydrogenase, abnormal echocardiogram, prolonged QT interval on electrocardiogram | Prescribed by clinicians. Not authorized by EMA for IBD + not mentioned on BCFI as treatment for IBD.  Side-effects obtained from the leaflet |
| 38 | Depo-medrol | Methylprednisolon | CD and UC | Not available | Opportunistic infections, infections, infections at the site of injection, activation of latent infections, leukocytosis, drug hypersensitivity, anaphylactic reaction, anaphylactoid reaction, Cushing syndrome, hypopituitarism, steroid withdrawal syndrome, metabolic acidosis, epidural lipomatosis, sodium retention, fluid retention, alkalosis hypokalemia, dyslipidemia, glucose tolerance decreased, insulin requirement increased, lipomatosis, increased appetite, affective disorder, psychotic disorder, mental disorder, personality change, confusional state, anxiety, mood swings, abnormal behavior, insomnia, irritability, increased intracranial pressure, convulsion, amnesia, cognitive impairment, dizziness, headache, Chorioretinopathy, blindness, cataract, glaucoma, exophthalmos, blurred vision, vertigo, congestive heart failure, thrombotic events, hypertension, hypotension, pulmonary embolism, hiccups, peptic ulcer, intestinal perforation, gastric bleeding, pancreatitis, peritonitis, ulcerative esophagitis, esophagitis, abdominal distension,  abdominal pain, diarrhea, dyspepsia, nausea, hepatitis, increase in liver enzymes, angioedema, hirsutism, petechiae, ecchymosis, skin atrophy, erythema, hyperhidrosis, skin stretch marks, rash, pruritus, urticaria, acne, skin hyperpigmentation, skin hypopigmentation, muscle weakness, myalgia, myopathy, muscle atrophy, osteoporosis; osteonecrosis, pathological fracture, neuropathic arthropathy, arthralgia, growth retardation, spinal cord compression fracture, tendon rupture, irregular periods, sterile abscess, decreased healing, edema peripheral, fatigue, malaise, injection site reaction, intraocular pressure increased, carbohydrate tolerance decreased, blood potassium decreased, urine calcium increased, alanine aminotransferase increased, aspartate aminotransferase increased, blood alkaline phosphatase increased, blood urea increased, suppression of skin test reactions | Not found on the EMA site. BCFI: can be used for both UC and CD. Side-effects obtained from the leaflet |
| 39 | Medrol | Methylprednisolon | CD and UC | Not available | Opportunistic infections, infections, peritonitis, leukocytosis, drug hypersensitivity, anaphylactic reaction, anaphylactoid reaction, Cushing syndrome, hypopituitarism, steroid withdrawal syndrome, metabolic acidosis, epidural lipomatosis, sodium retention, fluid retention, alkalosis hypokalemia, dyslipidemia, glucose tolerance decreased, insulin requirement increased, lipomatosis, increased appetite, negative nitrogen balance, affective disorder, psychotic disorder, mental disorder, personality change/disorder, confusional state, anxiety, mood swings, abnormal behavior, insomnia, irritability, increased intracranial pressure, convulsion, amnesia, cognitive impairment, dizziness, headache, chorioretinopathy, cataract, glaucoma, exophthalmos, blurred vision, vertigo, congestive heart failure, tachycardia, thrombotic events, hypertension, hypotension,  pulmonary embolism, hiccups, hepatitis, increase in liver enzymes, peptic ulcer, intestinal perforation, gastric bleeding, pancreatitis, ulcerative esophagitis, esophagitis, abdominal distension, abdominal pain, diarrhea, dyspepsia, nausea, vomiting, angioedema, hirsutism, petechiae, ecchymosis, skin atrophy, erythema, hyperhidrosis, skin stretch marks, pruritus, urticaria, acne, muscle weakness, myalgia, myopathy, muscle atrophy, osteoporosis; osteonecrosis, pathological fracture, neuropathic arthropathy, arthralgia, growth retardation,  irregular periods, decreased healing, edema peripheral, fatigue, malaise, intraocular pressure increased, carbohydrate tolerance decreased, blood potassium decreased, increased aspartate aminotransferase, urine calcium increased, alanine aminotransferase increased, blood alkaline phosphatase increased, blood urea increased, suppression of skin test reactions | Not found on the EMA site. BCFI: can be used for both UC and CD. Side-effects obtained from the leaflet |
| 40 | Solu-Medrol | Methylprednisolon | CD and UC | Not available | Opportunistic infections, infections, peritonitis, leukocytosis, drug hypersensitivity, anaphylactic reaction, anaphylactoid reaction, Cushing syndrome, hypopituitarism, steroid withdrawal syndrome, metabolic acidosis, sodium retention, fluid retention, alkalosis hypokalemia, dyslipidemia, glucose tolerance decreased, insulin requirement increased, lipomatosis, increased appetite, affective disorder, psychotic disorder, mental disorder, personality change/disorder, confusional state, anxiety, mood swings, abnormal behavior, insomnia, irritability, epidermal lipomatosis, increased intracranial pressure, convulsion, amnesia, cognitive impairment, dizziness, headache, chorioretinopathy, cataract, glaucoma, exophthalmos, blurred vision, vertigo, congestive heart failure, arrhythmia, tachycardia, bradycardia, cardiac arrest, thrombotic events, hypertension, hypotension,  pulmonary embolism, hiccups, hepatitis, increase in liver enzymes, peptic ulcer, intestinal perforation, gastric bleeding, pancreatitis, ulcerative esophagitis, esophagitis, abdominal distension, abdominal pain, diarrhea, dyspepsia, nausea, vomiting, angioedema, hirsutism, petechiae, ecchymosis, skin atrophy, erythema, hyperhidrosis, skin stretch marks, pruritus, urticaria, acne, hypopigmentation of the skin,  muscle weakness, myalgia, myopathy, muscle atrophy, osteoporosis; osteonecrosis, pathological fracture, neuropathic arthropathy, arthralgia, growth retardation,  irregular periods, decreased healing, edema peripheral, fatigue, malaise, injection site reaction, intraocular pressure increased, carbohydrate tolerance decreased, blood potassium decreased, increased aspartate aminotransferase, urine calcium increased, alanine aminotransferase increased, blood alkaline phosphatase increased, blood urea increased, suppression of skin test reactions | Not found on the EMA site. BCFI: can be used for both UC and CD. Side-effects obtained from the leaflet |
| 41 | Clipper | Beclomethasone | CD and UC | Not available | Anxiety, headache, somnolence, nausea, constipation abdominal pain, muscle cramps, menorrhagia, flu-like symptoms, pyrexia, decreased plasma cortisol levels, oropharyngeal candidiasis, lymphopenia, monocytopenia, granulocytosis, suppression of the adrenal function, moon face, obesity, headache, hypertension, cataract, glaucoma, lipohypertrophy, rosacea, osteoporosis, blurred vision, hiccups, affective disorder, psychotic disorder, behavioral disorder, irritability, anxiety, insomnia, confusion, amnesia | Not found on the EMA site. BCFI: can be used for both UC and CD. Side-effects obtained from the leaflet |
| 42 | Budenofalk | Budesonide | CD and UC | Not available | Cushing syndrome, growth retardation, glaucoma, cataract, blurred vision, dyspepsia, abdominal pain, ulceration, pancreatitis, constipation, increased susceptibility to infections, joint pain, muscle pain, muscle weakness, muscle spasms, osteoporosis, osteonecrosis, headache, depression, irritability, euphoria, psychomotor hyperactivity, anxiety, aggression, exanthema, petechiae, decreased wound healing, dermatitis, ecchymosis, increased risk of thrombosis, vasculitis, fatigue, malaise | Not found on the EMA site. BCFI: can be used for both UC and CD. Side-effects obtained from the leaflet |
| 43 | Budesonide Ferring | Budesonide | CD and UC | Not available | Flu, leukocytosis, insomnia, mood disorders and mood swings, headache, dizziness, nausea, gastrointestinal and abdominal pain, abdominal distension, dry mouth, dyspepsia, flatulence, acne, myalgia, back pain, muscle spasms, fatigue, edema, decreased blood cortisol, anaphylactic reaction, Cushing syndrome, growth retardation with children, hypokalemia, personality change, insomnia, hyperactivity, anxiety, aggression, depression, tremor, cataract, glaucoma, blurred vision, palpitations, dyspepsia, urticaria, exanthema, ecchymosis, muscle cramps, menstrual disorders, psychomotor hyperactivity | Not found on the EMA site. BCFI: can be used for both UC and CD. Side-effects obtained from the leaflet |
| 44 | Entocort | Budesonide | CD and UC | Not available | Anaphylactic reaction, Cushing characterisitcs, growth retardation with children, hypokalemia, behavior change, mood change, depression, insomnia, anxiety, tremor, psychomotor hyperactivity, cataract, glaucoma, blurred vision, palpitations, dyspepsia, urticaria, exanthema, ecchymosis, muscle cramps, menstrual disorders | Not found on the EMA site. BCFI: can be used for both UC and CD. Side-effects obtained from the leaflet |
| 45 | Jorveza | Budesonide | CD and UC | 2 studies with eosinophilic oesophagitis patients found reduced eosinophils levels and symptom improvement compared with Jorvez compared with placebo | Fungal infections in the mouth, pharynx (throat) and oesophagus | Not authorized by EMA for CD and UC. BCFI: can be used for patients with CD and UC  🡪 Source: EMA for other indication |

# 2B – Overview of unfavorable effects of inflammatory bowel disease treatments assessed by the European Medicines Agency as described in the European Assessment Reports (EPARs) or by the Federal Agency for Medicines and Health Products in their leaflets

| Risks/unfavourable effects | Number of medical products/EPARs/leaflets |
| --- | --- |
| **1. Blood and lymphatic system disorders** | |
| Anemia | 14 |
| Leukopenia (including agranulocytosis) | 16 |
| Thrombocytopenia | 15 |
| Leukocytosis (including eosinophilia) | 12 |
| Pancytopenia | 10 |
| Neutropenia | 7 |
| Erythroid hypoplasia | 2 |
| Hypoprothrombinemia | 3 |
| Coagulopathies | 3 |
| Thrombotic thrombocytopenic purpura | 4 |
| Thrombotic microangiopathy | 4 |
| Hemolytic uremic syndrome | 1 |
| Microangiopathic hemolytic anemia | 1 |
| Episodes of bone marrow suppression or suppression of the hematopoiesis | 15 |
| Lymphoproliferative disorders | 6 |
| **2. Endocrine disorders** | |
| Hirsutism | 7 |
| Cushing syndrome or characteristics | 7 |
| Hypopituitarism | 3 |
| Steroid withdrawal syndrome | 3 |
| Suppression of the adrenal function | 1 |
| **3. Nutritional and metabolic disorders** | |
| Diabetes mellitus | 7 |
| Hyperglycemia | 4 |
| Hypoglycemia | 4 |
| Hyperkalemia | 6 |
| Hypokalemia | 8 |
| Hyponatremia | 3 |
| Hypervolemia | 2 |
| Hyperuricemia | 4 |
| Hypomagnesemia | 4 |
| Hypocalcemia | 3 |
| Hypercholesterolemia | 3 |
| Hyperlipidemia | 4 |
| Hypertriglyceridemia | 3 |
| Hypophosphatemia | 3 |
| Hyperphosphatemia | 3 |
| Hypoproteinemia | 3 |
| Negative nitrogen balance | 1 |
| Metabolic acidosis | 6 |
| Other electrolyte imbalances | 4 |
| Increased insulin requirement | 3 |
| Decreased glucose tolerance | 3 |
| Decreased appetite | 3 |
| Increased appetite | 3 |
| Anorexia | 2 |
| Obesity | 1 |
| Dehydration or fluid retention | 6 |
| Fluid overload | 1 |
| Sodium retention | 3 |
| Lipomatosis | 3 |
| Epidural lipomatosis | 3 |
| Dyslipidemia | 3 |
| **4. Psychological disorders** | |
| Insomnia | 11 |
| Symptoms of anxiety | 11 |
| Confusion and/or disorientation | 8 |
| Depression or depressed feelings | 9 |
| Mood disorders or swings | 11 |
| Nightmares | 3 |
| Hallucinations | 3 |
| Mental disorders | 6 |
| Psychotic disorders/reactions | 7 |
| Affective disorder | 4 |
| Personality change/disorder | 4 |
| Abnormal behavior or behavioral disorder (including euphoria and aggression) | 7 |
| Irritability | 5 |
| **5. Nervous System Disorders** | |
| Headache | 39 |
| Migraine | 1 |
| Tremor | 8 |
| Dizziness | 14 |
| Somnolence/drowsiness | 5 |
| Convulsions/seizures | 10 |
| Impaired consciousness | 3 |
| Cognitive impairment/disorder/dysfunction | 5 |
| Nervous system disorders | 3 |
| (Peripheral) neuropathy | 16 |
| Motoric polyneuropathy | 1 |
| Demyelinating disorder | 1 |
| Sensibility and feeling disorders (including hypoaesthesia) | 5 |
| Speech and language disorders (including dysarthria and aphasia) | 5 |
| Impaired ability to write | 3 |
| Encephalopathy | 7 |
| Cerebral hemorrhages and strokes | 3 |
| Coma | 3 |
| Paralysis and/or (hemi)paresis | 6 |
| Paresthesia and/or dysesthesia | 5 |
| Amnesia | 7 |
| Hypertonia | 3 |
| Myasthenia | 3 |
| Increased intracranial pressure | 3 |
| Psychomotor hyperactivity | 2 |
| Meningism | 1 |
| Meningitis | 1 |
| **6. Eye disorders** | |
| Eye or vision disorders | 5 |
| Blurred vision or reduced/impaired vision | 13 |
| Photophobia | 3 |
| Cataract | 10 |
| (Temporary) Blindness | 6 |
| Glaucoma | 7 |
| Retinopathy | 1 |
| Exophthalmos | 3 |
| Chorioretinopathy | 3 |
| Conjunctivitis | 3 |
| **7. Balance organ and ear disorders** | |
| Tinnitus | 4 |
| (Neurosensory) Hearing loss (hypoacusis) | 3 |
| Deafness | 3 |
| Vertigo | 3 |
| Earache | 1 |
| **8. Cardiac disorders** | |
| Tachycardia | 6 |
| Bradycardia | 1 |
| (Congestive) Heart failure | 7 |
| Ventricular arrhythmia and cardiac arrest | 4 |
| Supraventricular Arrhythmia | 4 |
| Ischemic coronary artery disease | 3 |
| Cardiomyopathy | 3 |
| Ventricular hypertrophy | 3 |
| Myocarditits | 4 |
| Palpitations | 5 |
| Pericarditis | 6 |
| Pericardial effusion | 6 |
| Pericardial tamponade | 1 |
| Torsade de Pointes | 3 |
| **9. Blood vessel disorders** | |
| Hypertension | 13 |
| Hypotension | 6 |
| Bleeding | 3 |
| Flushing | 1 |
| Shock | 3 |
| Infarction | 3 |
| Thromboembolic and ischemic events | 9 |
| Deep vein thrombosis in the leg | 3 |
| Increased risk of thrombosis | 1 |
| Vascular hypotensive disorders | 3 |
| Peripheral vascular disorders | 3 |
| (Allergic) Vasculitis | 5 |
| Sepsis | 4 |
| **10. Respiratory, thoracic and mediastinal disorders** | |
| Parenchymal lung abnormalities | 3 |
| Interstitial lung disease | 1 |
| Pneumonia | 5 |
| Pneumonitis | 6 |
| Pleuropericarditis | 1 |
| Pharyngitis | 6 |
| Dyspnea or shortness of breath | 9 |
| Cough | 8 |
| Hiccups | 4 |
| Pleural effusion | 6 |
| Nasal congestion and inflammation | 3 |
| Respiratory failure | 3 |
| Respiratory disorders | 3 |
| Pulmonary embolism | 3 |
| Pulmonary/respiratory fibrosis | 7 |
| Pneumocystis jirovecii pneumonia | 1 |
| Asthma or asthmatic bronchitis | 4 |
| Acute respiratory distress syndrome | 3 |
| Chronic obstructive bronchopneumopathy | 2 |
| (Allergic) alveolitis | 4 |
| Tracheal spasms or bronchospasms | 4 |
| Pulmonary eosinophilia | 4 |
| Pulmonal infiltration | 4 |
| **11. Gastrointestinal Disorders** | |
| Diarrhea | 20 |
| Nausea | 27 |
| Vomiting | 15 |
| Constipation/obstipation | 5 |
| Flatulence | 9 |
| Loss of appetite | 5 |
| Gastrointestinal complaints/symptoms/problems | 5 |
| Gastrointestinal and/or abdominal pain | 22 |
| Gastrointestinal bleeding | 9 |
| (Gastrointestinal) ulceration and/or perforation | 12 |
| Rectal polyp | 1 |
| Pseudocysts in the pancreas | 3 |
| Inflammatory reactions of the gastrointestinal system or enteritis | 6 |
| Swelling and enlargement of the stomach/abdominal distension | 9 |
| Signs and symptoms of poor digestion/dyspepsia | 14 |
| Soft stools | 3 |
| Melena | 2 |
| Hematemesis | 2 |
| Acute and/or chronic pancreatitis | 19 |
| Adynamic/paralytic ileus | 3 |
| Gastroesophageal reflux disease | 3 |
| Impaired gastric emptying | 3 |
| Incomplete ileus | 3 |
| Anal discomfort | 1 |
| Ascites | 3 |
| Stomatitis | 6 |
| Peritonitis | 3 |
| Pancolitis | 3 |
| Diverticulitis | 1 |
| Appendicitis | 1 |
| (Ulcerative) Esophagitis | 3 |
| Gingivitis | 3 |
| Gingival hyperplasia | 1 |
| **12. Liver and bile disorders** | |
| Bile duct disorders | 1 |
| Bile duct stenose | 2 |
| Bile obstruction/cholestasis | 7 |
| Inflammation of the bile ducts/cholangitis | 3 |
| Abnormal liver function | 2 |
| Hepatocellular damage | 5 |
| Hepatitis | 13 |
| Hepatoxicity | 5 |
| Liver necrosis | 1 |
| Liver failure | 6 |
| Jaundice | 4 |
| Cirrhosis | 5 |
| Fibrosis | 4 |
| Vena-occlusive liver disease | 3 |
| Thrombosis of the hepatic artery | 3 |
| **13. Skin and subcutaneous tissue disorders** | |
| Rash (exanthema) | 16 |
| Dermatitis (including erythema) | 12 |
| Pruritus | 11 |
| Alopecia | 15 |
| Excessive hair growth (hypertrichosis) | 1 |
| Acne | 12 |
| Increasing sweating (hyperhidrosis) | 6 |
| Photosensitivity/photosensitization | 11 |
| Toxic epidermal necrolysis (Lyell’s syndrome) | 9 |
| Stevens-Johnson syndrome | 18 |
| Angioedema | 5 |
| Petechiae | 5 |
| Urticaria | 11 |
| Rosacea or purpura | 2 |
| Ecchymosis (bruises) | 9 |
| Cellulitis | 3 |
| Skin atrophy or necrosis | 5 |
| Skin stretch marks or skin striae | 3 |
| Skin hypopigmentation | 4 |
| Skin hyperpigmentation | 4 |
| (Herpetiform) Skin eruption | 3 |
| Erosion of psoriasis plaques | 2 |
| Increase in rheumatoid nodules | 1 |
| Skin ulcers | 3 |
| Herpes zoster | 2 |
| **14. Bone, muscle and connective tissue disorders** | |
| Joint pain/arthralgia | 18 |
| Joint complaints | 2 |
| Joint disorders | 1 |
| Bone pain | 9 |
| Back pain | 5 |
| Muscle weakness | 5 |
| Muscle spasms or cramps | 9 |
| Muscle pain/myalgia | 23 |
| Myopathy | 4 |
| Muscle atrophy | 3 |
| Pain in extremity | 4 |
| Decreased mobility | 3 |
| Osteoporosis | 8 |
| Osteonecrosis | 5 |
| Pathological fracture | 3 |
| Neuropathic arthropathy | 3 |
| Growth retardation (with children) | 6 |
| Spinal cord compression fracture | 1 |
| Tendon rupture | 1 |
| Stress fracture | 3 |
| **15. Renal and urinary disorders** | |
| Renal function disorders/insufficiency | 10 |
| (Acute) Renal/kidney failure | 8 |
| Kidney problems or impaired kidney function | 5 |
| (Toxic) nephropathy | 9 |
| Tubular necrosis | 3 |
| Problems with urination or impaired micturition | 3 |
| Discoloration of the urine | 1 |
| Oliguria | 4 |
| Anuria | 4 |
| Proteinuria | 2 |
| Dysuria | 2 |
| Bladder and urethra symptoms | 3 |
| Inflammation and ulceration of the urinary bladder | 1 |
| Hemolytic uremic syndrome | 3 |
| Urinary tract abnormalities or infections | 2 |
| Acute and/or chronic interstitial nephritis | 4 |
| Hemorrhagic cystitis | 3 |
| **16. Immune system disorders** | |
| Allergic reaction | 6 |
| Anaphylactic reaction/shock | 11 |
| Anaphylactoid reactions | 6 |
| (Drug) hypersensitivity reactions | 10 |
| Hypogammaglobulinemia | 3 |
| Lupus like disorders | 12 |
| **17. Infections and parasitic diseases** | |
| Increased susceptibility to infections/opportunistic infections (viral, bacterial and fungal) | 10 |
| Infections (viral, bacterial and fungal) | 27 |
| Infections at the site of injection | 1 |
| Activation of latent infections | 5 |
| Progressive multifocal leuko-encephalopathy associated with JC-virus | 5 |
| Nephropathy associated with BK-virus | 3 |
| Upper respiratory tract infections (including nasopharyngitis) | 19 |
| **18. Neoplasms** | |
| Increased risk for developing malignancies (both benign and malign neoplasm) | 3 |
| Skin cancers (melanomas and non-melanomas) | 3 |
| Sarcomas (Kaposi and non-Kaposi sarcomas) | 3 |
| Cervical cancer (“in situ”) | 3 |
| Acute myeloid leukemia, lymphoma, myelodysplastic syndrome or other blood cancers | 16 |
| **19. Reproductive system and breast disorders** | |
| Painful menstruation/dysmenorrhea | 3 |
| Irregular menstruation/periods or menstrual disorders including menorrhagia | 10 |
| Gynecomastia | 4 |
| Uterine bleeding | 3 |
| Inflammation and ulceration of the vagina | 1 |
| Vaginal discharge | 3 |
| Ovogenesis/spermatogenesis disorder | 2 |
| (Temporal) Oligospermia | 7 |
| Loss of libido | 3 |
| Impotence | 3 |
| Infertility | 2 |
| **20. Pregnancy, perinatal period and puerpium** | |
| Congenital abnormalities | 2 |
| Abortion | 2 |
| **21. General disorders and administration site disorders** | |
| (Drug) Fever/febrile illness/pyrexia | 12 |
| Pain and discomfort | 10 |
| Asthenia/weakness | 6 |
| (Peripheral) Edema | 8 |
| Fatigue | 9 |
| Malaise | 4 |
| Disturbed sensation of body temperature | 3 |
| Temperature intolerance | 3 |
| Flu-like symptoms | 4 |
| Nervous feeling | 3 |
| Abnormal sensation/feeling | 4 |
| Multi-organ failure | 3 |
| Sensation of a pressing feeling in the chest or chest tightness | 6 |
| Falling | 3 |
| Ulcers | 3 |
| Lumps/nodes | 2 |
| Abscess | 1 |
| Thirsty/dry mouth | 4 |
| Change in sense of taste/taste abnormalities | 2 |
| Increase in adipose tissue | 3 |
| Decreased wound healing | 5 |
| Injection site reaction (including irritation, redness, itching, bleeding, pain or swelling) | 17 |
| **22. Investigations** | |
| Abnormal red blood cell analysis results | 3 |
| Abnormal results of coagulation and bleeding tests | 3 |
| Abnormal results liver function test/parameters | 9 |
| Abnormal results of pancreatic enzymes | 1 |
| Abnormal ECG | 3 |
| Abnormal pulse and heart rate | 3 |
| Abnormal echocardiogram | 3 |
| Prolonged QT interval on electrocardiogram | 2 |
| Increased liver enzymes | 8 |
| Increased alkaline phosphatase in the blood | 6 |
| Increased calcium in the urine | 3 |
| Increased alanine aminotransferase | 3 |
| Increased aspartate aminotransferase | 3 |
| Increased intraocular pressure | 3 |
| Increased blood urea | 3 |
| Elevated amylase | 5 |
| Elevated blood lactate dehydrogenase | 3 |
| Decreased carbohydrate tolerance | 3 |
| Decreased blood potassium | 3 |
| Decreased plasma or blood cortisol levels | 2 |
| Decreased serum albumin | 3 |
| Suppression of skin test reactions | 3 |
| Weight gain | 4 |
| Weight loss | 3 |

# 3 – Overview of endpoints and adverse events reported in inflammatory bowel disease phase 3 clinical trials

EU clinical trial database: Clinicaltrialregister.eu

- Search term: Inflammatory bowel disease OR Crohn's disease OR Ulcerative colitis
- No restrictions on country/age
- Trial status: completed, ongoing, restarted
- Trial phase: phase III
- 2011 onwards

🡪 65 results (last search on 26^st^ of March 2021)

Exluded if:

- Medical condition is not for treatment of IBD
- Non-english title
- Investigating diagnostics (e.g. PET)
- For treatment of MM symptoms and drug-induced side effects
- Pharmacogenomic studies
- Studies assessing bioavailability
- Studies not aiming to measure efficacy (e.g. safety studies)

🡪 58 results

| **I**. Primary and secondary endpoints |
| --- |
| **1. Response to treatment (including changes in symptoms)** |
| Clinical remission |
| Maintenance of clinical remission |
| Mucosal healing |
| Clinical response |
| Improvement in endoscopic appearance of the mucosa |
| Endoscopic and histological remission |
| Remission |
| (Sustained) steroid-free remission |
| Steroid and biologic treatment-free remission defined as total Mayo score ≤1without rectal bleeding |
| (Deep) endoscopic remission |
| Endoscopic subscore |
| Hospitalization |
| Chirurgic procedures |
| Rate of clinical improvement |
| Time to relapse |
| Frequency of relapses |
| Percentage of participants undergoing surgery for ulcerative colitis (Including Colectomy) |
| Percentage of participants hospitalized for ulcerative colitis |
| Steroid-free remission without surgery |
| Fecal calprotectin |
| Hs-CRP levels |
| Flogosis indexes |
| Change in Hb concentration |
| Number of stools per week |
| Number of bloody stools per week |
| Number of days with urgency per week |
| Time to first resolution of clinical symptoms |
| Symptomatic remission |
| Deep remission |
| Change from baseline in ulcerative colitis bowel movement signs and symptoms of abdominal symptoms |
| Rectal bleeding evaluation |
| Time to rectal bleeding score |
| Change from baseline in IBDQ score |
| Change from baseline in rectal bleed and stool frequency subscore |
| Bone mineralization |
| PUCAI remission |
| (Partial) Mayo Clinic Score (pMCS) |
| Incidence of serious infections |
| Change in UC bowel movement signs and symptoms, as assessed by the Ulcerative Colitis-Patient-Reported Outcome Signs and Symptoms (UC-PRO/SS) measure |
| Laboratory assessments |
| Physician's Global Assessment |
| The proportion of subjects who are on concomitant narcotic pain medications for CD |
| The proportion of subjects able to eliminate concomitant narcotic pain medication use for CD |
| The proportion of subjects who are on concomitant narcotic pain medications for any reason |
| The proportion of subjects able to eliminate concomitant narcotic pain medication use for any reason |
| Time to colectomy |
| Rate of colectomy |
| Modulation of at least 10% of those with Bact2 enterotype to a different enterotype |
| **2. Quality of life** |
| Change from baseline in health-related quality of life |
| Change from baseline in patient-reported health-related quality of life as assessed by the Inflammatory Bowel Disease Questionnaire |
| Patient’s Global Satisfaction |
| Patient’s acceptance and preference of trial drug |
| Health related quality of life evaluation using an inflammatory bowel disease questionnaire and a short-form health survey with 36 questions (SF-36) |
| Changes from baseline in WPAI-UC, and EuroQual (EQ-5D) scores |
| The change in the DUCS score |
| To evaluate change in UC daily coping strategies, daily life impact, and emotional, as assessed by the UC-PRO Daily Coping (UC-PRO-DC), UC-PRO Daily Life Impact (UC-PRO-DLI), and UC-PRO Emotional Impact (UC-PRO-EI) measures |
| **3. Other** |
| Safety analyses |
| Incidence and severity of adverse events |
| Incidence of serious adverse events |
| Incidence and severity of infection-related adverse events |
| Incidence of serious infection-related adverse events |
| Incidence and severity of injection-site reactions |
| Incidence of adverse events leading to study drug discontinuation |
| Incidence of laboratory abnormalities |
| Incidence of malignancies |
| Incidence and severity of hypersensitivity reaction events |
| **II. Adverse events** |
| **1. Respiratory, thoracic and mediastinal disorders** |
| Dyspnoea |
| Pneumothorax spontaneous |
| Cough |
| Productive cough |
| Epistaxis |
| Oropharyngeal pain |
| Rhinitis allergic |
| Rhinorrhea |
| Wheezing |
| Emphysema |
| Nasal polyps |
| Nasal septum deviation |
| Pulmonary embolism |
| Sinus polyp |
| Pulmonary sarcoidosis |
| Pneumothorax |
| Vasomotor rhinitis |
| Hiccups |
| Pulmonary embolism |
| **2. Vascular disorders** |
| Hypovolaemic shock |
| Thrombophlebitis (superficial) |
| Embolism venous |
| Hypertension |
| Deep vein thrombosis |
| **3. Neoplasms benign, malignant and unspecified** |
| Adenocarcinoma of colon |
| Bowen's disease |
| Invasive ductal breast carcinoma |
| Squamous cell carcinoma of skin |
| Bladder cancer |
| Fibromatosis |
| Malignant melanoma |
| Non-small cell lung cancer |
| Oesophageal adenocarcinoma |
| Uterine leiomyoma |
| Rectal adenocarcinoma |
| Malignant melanoma of eyelid |
| Prostate cancer |
| Testicular seminoma |
| Anaplastic oligodendroglioma |
| Melanocytic naevus |
| Fibroadenoma of breast |
| Cervix neoplasm |
| Neuroendocrine tumour |
| **4. Nervous system disorders** |
| Brain stem haemorrhage |
| Cerebrovascular accident |
| Dysgraphia |
| Seizure |
| Nerve root compression |
| Headache |
| Loss of consciousness |
| Tremor |
| Generalised tonic-clonic seizure |
| Haemorrhagic stroke |
| Sciatica |
| Altered state of consciousness |
| Chronic inflammatory demyelinating polyradiculoneuropathy |
| Hypoaesthesia |
| Ischaemic stroke |
| Mononeuropathy |
| Cerebral haemorrhage |
| Dizziness |
| Intracranial venous sinus thrombosis |
| Syncope |
| Dysgeusia |
| Migraine |
| **5. General disorders and administration site conditions** |
| Therapeutic response decreased |
| Fatigue |
| Inflammation |
| Peripheral swelling |
| Pyrexia |
| Chest pain |
| Malaise |
| Gait disturbance |
| Drug resistance |
| Pyrexia |
| Pseudopolyp |
| Accidental death |
| Asthenia |
| Sudden cardiac death |
| Drug withdrawal syndrome |
| Tablet physical issue |
| Irritability |
| Local swelling |
| Oedema peripheral |
| Haemoglobin decreased |
| **6. Investigations** |
| C-reactive protein increased |
| Hepatic enzyme increased |
| Monocyte count decreased |
| Neutrophil count decreased |
| White blood cell count decreased |
| Blood creatine phosphokinase increased |
| Weight decreased |
| Faecal calprotectin increased |
| Lipase increased |
| Vitamin D decreased |
| Haemoglobin decreased |
| Body temperature increased |
| Weight increased |
| Blood fibrinogen increased |
| Eosinophil count increased |
| Alanine aminotransferase abnormal |
| Aspartate aminotransferase abnormal |
| Blood alkaline phosphatase abnormal |
| Gamma-glutamyltransferase abnormal |
| **7. Cardiac disorders** |
| Angina pectoris |
| Myocardial ischaemia |
| Pericarditis |
| Myocardial infarction |
| Cardiac arrest |
| Cardiac failure congestive |
| Coronary artery disease |
| Tachycardia |
| Silent myocardial infarction |
| Bundle branch block left |
| Atriventricular block first degree |
| **8. Blood and lymphatic system disorders** |
| Anaemia |
| Neutropenia |
| Thrombocytosis |
| Iron deficiency anaemia |
| Lymphadenopathy |
| Pancytopenia |
| Thrombocytopenia |
| Leukopenia |
| **9. Gastro intestinal disorders** |
| Colitis |
| Inflammatory bowel disease |
| Diarrhoea |
| Small intestinal obstruction |
| Abdominal pain |
| Ileus |
| Inguinal hernia |
| Peritoneal haemorrhage |
| Proctitis |
| Umbilical hernia |
| Incarcerated umbilical hernia |
| Dyspepsia |
| Enteritis |
| Pancreatitis |
| Constipation |
| Gastrooesophageal reflux disease |
| Nausea |
| Vomiting |
| Gastrointestinal haemorrhage |
| Colitis ulcerative |
| Anogenital dysplasia |
| Duodenal ulcer perforation |
| Haemorrhoids |
| Large intestinal stenosis |
| Mallory-weiss syndrome |
| Rectal haemorrhage |
| Upper gastrointestinal haemorrhage |
| Acute abdomen |
| Large intestine perforation |
| Abdominal pain |
| Anal fistula |
| Proctalgia |
| Megacolon |
| Colon dysplasia |
| Abdominal adhesions |
| Anal fissure |
| Crohn's disease |
| Enterovesical fistula |
| Haematochezia |
| Ileal stenosis |
| Intestinal stenosis |
| Melaena |
| Pancreatitis acute |
| Defaecation urgency |
| Flatulence |
| Frequent bowel movements |
| Painful defaecation |
| Cheilosis |
| Mouth ulceration |
| Toothache |
| Abdominal distension |
| Abdominal mass |
| Hyperchlorhydria |
| **10. Skin and subcutaneous tissue disorders** |
| Dermatitis |
| Psoriasis |
| Erythema nodosum |
| Hangnail |
| Rash |
| Dermatitis acneiform |
| Rash maculo-papular |
| Excessive skin |
| Linear IGA disease |
| Night sweats |
| Pemphigoid |
| Subcorneal pustular dermatosis |
| Acne |
| Dermatosis |
| Pruritus generalised |
| Acute febrile neutrophilic dermatosis |
| Pyoderma gangrenosum |
| Erythema |
| Eczema |
| Hyperhidrosis |
| Pruritus |
| **11. Musculoskeletal and connective tissue disorders** |
| Intervertebral disc protrusion |
| Muscular weakness |
| Pain in extremity |
| Arthralgia |
| Musculosketal pain |
| Osteoarthritis |
| Spondylolisthesis |
| Back pain |
| Osteitis |
| Costochondritis |
| Bone pain |
| Myalgia |
| Neck pain |
| Torticollis |
| Muscle tightness |
| **12. Metabolism and nutrition disorders** |
| Hyponatraemia |
| Hypercholesterolaemia |
| Dehydration |
| Diabetes mellitus |
| Hypovolaemia |
| Hypokalemia |
| Type 2 diabetes mellitus |
| Malnutrition |
| Decreased appetite |
| Increased appetite |
| Dyslipidaemia |
| Electrolyte imbalance |
| Hypoglycaemia |
| Hypoalbuminaemia |
| **13. Renal and urinary disorders** |
| Acute kidney injury |
| Ureterolithiasis |
| Glycosuria |
| Haematuria |
| Nephrotic syndrome |
| Renal failure |
| Urinary retention |
| Stress urinary incontinence |
| **14. Immune system disorders** |
| Drug hypersensitivity |
| **15. Psychiatric disorders** |
| (Major) depression |
| Binge drinking |
| Bipolar disorder |
| Insomnia |
| Alcoholism |
| Fear |
| Agression |
| Anxiety |
| Restlessness |
| **16. Eye disorders** |
| Blindness |
| Noninfective conjunctivitis |
| Eyelid ptosis |
| Optic atrophy |
| Chorioretinopathy |
| Visual impairment |
| Blepharospasm |
| Conjunctivitis |
| Scleritis |
| **17.** **Pregnancy, puerperium and perinatal conditions** |
| Abortion spontaneous |
| Unintended pregnancy |
| Foetal death |
| Foetal distress syndrome |
| **18.** **Surgical and medical procedures** |
| Wound drainage |
| Cartilage graft |
| Abortion induced |
| Tooth extraction |
| **19. Hepatobiliary disorders** |
| Cholecystitis |
| Bile duct stone |
| Hepatitis |
| Portosplenomesenteric venous thrombosis |
| Cholestatis |
| Cholelithiasis |
| Hepatic vein thrombosis |
| **20. Product issues** |
| Device dislocation |
| **21. Endocrine disorders** |
| Hyperthyroidism |
| **22.** **Reproductive system and breast disorders** |
| Prostatomegaly |
| Uterine scar |
| Perineal disorder |
| Prostatitis |
| Dysmenorrhoea |
| Breast discomfort |
| Endometriosis |
| Galactorrhoea |
| **23.** **Injury, poisoning and procedural complications** |
| Traumatic haemothorax |
| Ankle fracture |
| Stab wound |
| Post procedural complication |
| Thoracic vertebral fracture |
| Hand fracture |
| Wrist fracture |
| Joint injury |
| Lower limb fracture |
| Lumbar vertebral fracture |
| Spinal compression fracture |
| Humerus fracture |
| Meniscus injury |
| Patella fracture |
| Post procedural haemorrhage |
| Procedural intestinal perforation |
| Procedural pain |
| Radius fracture |
| Rib fracture |
| Arthropod bite |
| Joint dislocation |
| Upper limb fracture |
| Craniocerebral injury |
| Clavicle fracture |
| Scapula fracture |
| Ligament rupture |
| Road traffic accident |
| Facial bones fracture |
| Jaw fracture |
| Pneumothorax traumatic |
| Injury corneal |
| Retinal injury |
| **24.** **Infections and infestations** |
| Appendicitis |
| Anal abscess |
| Clostridium difficile colitis |
| Clostridium difficile infection |
| Cytomegalovirus infection |
| Liver abscess |
| Varicella |
| Wound infection |
| Pneumonia |
| Gastroenteritis viral |
| Nasopharyngitis |
| Upper respiratory tract infection |
| Enteritis infectious |
| Gastroenteritis |
| Meningitis aseptic |
| Pharyngitis |
| Urinary tract infection |
| Bronchitis |
| Influenza |
| Respiratory tract infection viral |
| Streptococcal infection |
| Bacterial diarrhoea |
| Tooth abscess |
| Viral infection |
| Vulvovaginal mycotic infection |
| Diverticulitis |
| Peritonsillar abscess |
| Subcutaneous abscess |
| Herpes zoster |
| Cytomegalovirus colitis |
| Erysipelas |
| External ear cellulitis |
| Eye infection toxoplasmal |
| Gastrointestinal infection |
| Pyelonephritis acute |
| Infection |
| Otitis externa |
| Pelvic inflammatory disease |
| Perineal abscess |
| Sepsis |
| Ternitis |
| Tuberculosis |
| Osteomyelitis |
| Peritonitis |
| Tonsillitis |
| Sinusitis |
| Abscess limb |
| Campylobacter gastroenteritis |
| Pneumonia bacterial |
| Cellulitis |
| Pyelonephritis |
| Ear infection |
| Otitis media |
| Rectal abscess |
| Wound sepsis |
| Pulpitis dental |
| Abdominal abscess |
| Rhinitis |
| Epstein-barr virus infection |
| Gastroenteritis |
| Gastroenteritis norovirus |
| Herpes simplex oesophagitis |
| Staphylococcal sepsis |
| Herpes simplex |
| Respiratory tract infection |
| Viral upper respiratory tract infection |
| Oral herpes |

1. *Following preference methods were considered:*

   *Preference exploration methods:* ***individual techniques*** *(interviews, complaint procedures),* ***group techniques*** *(Delphi method, focus group, public meeting, nominal group technique, citizens’ juries) and* ***concept mapping***

   *Preference elicitation methods:* ***discrete-choice based related techniques*** *(discrete choice experiment/conjoint analysis, best-worst scaling type 3, self-explicated conjoint, measure of value),* ***threshold related techniques*** *(standard gamble, time trade-off, person trade-off, starting known efficacy, test trade-off, threshold technique, contingent valuation),* ***rating related techniques*** *(constant sum scaling, repertory grid method, analytic hierarchy process, swing weighting, visual analogue scale, allocation of points, outcome prioritization tool) and* ***ranking related techniques*** *(qualitative discrimination process, q-methodology, control preferences scale, best-worst scaling type 1,2)* [↑](#footnote-ref-1)
