# Supplementary material for: Patient preferences for inflammatory bowel disease treatments: protocol development of a global preference survey using a discrete choice experiment
Source: Front Med (Lausanne). 2024 Aug 14;11:1418874. doi: 10.3389/fmed.2024.1418874 (PMC11349669; doi:10.3389/fmed.2024.1418874)
Supplement: Supplementary file 2 [file Table_2.DOCX]

***Supplementary Material 2***

**Patient Preferences for Inflammatory Bowel Disease Treatments: Protocol Development of a Global Preference Survey using a Discrete Choice Experiment**

**Attribute grading sheet**

Instructions

1. Please read the instructions below (on this page) and treatment characteristics (following pages) carefully. The treatment characteristics can be both positive and negative characteristics related to chronic inflammatory bowel disease, or the treatments used for it. These characteristics may include the following:

- Symptoms of inflammatory bowel disease
- Side-efffects of treatments
- Positive effects of treatments

These characteristics may be associated with treatments currently prescribed to patients or with treatments currently in development.

The chance these characteristics may occur in a specific patient may be very different for each of the characteristics; some are side effects of specific treatments and may occur very rarely while others more frequently. The chance these effects occur in a specific patient may depend on different factors (such as the current treatment, disease history, patient characteristics…).

1. If you find that one or more treatment characteristic(s) that you entered in the previous section are missing, please add this / these characteristic(s) to the end of the list under "Missing treatment characteristics". Please also include a description for that characteristic.

Do not hesitate to ask questions to Elise Schoefs (elise.schoefs@student.kuleuven.be or mobile: +32 476 08 14 36) if you do not understand something. Your opinion on these characteristics will be asked during the discussion, so it is important that you understand what they mean. Your answers to this ranking exercise can be further completed during the discussion if necessary.

| Characteristic | Explanation | Grading |
| --- | --- | --- |
| Characteristics related to the treatment efficacy | | |
| Short-term clinical response | The treatment reduces symptoms of the disease in the short-term |  |
| Long-term clinical remission | The treatment ensures that the disease is suppressed, disappears, and stays away for a long time |  |
| Prevention of flare-ups | The treatment prevents disease flare-ups whereby the complaints get worse, or whereby new complaints arise |  |
| Macroscopic healing of the intestinal mucosa | The treatment ensures an absence of fragilty, blood, damage, and sores in the intestinal mucosa |  |
| Microscopic healing of the intestinal mucosa | The treatment ensures an absence of abnormalities when the intestinal tissue (biopsies) is examined under a microscope |  |
| Transmural healing of the gut | The treatment ensures the absence of abnormalities in radiological examinations of the bowels (e.g., CT scan, MR scan, ultrasound scan) |  |
| Use of cortisone preparations | The treatment ensures that cortisone preparations (steroids) do not have to be used |  |
| Use of painkilling medication | The treatment ensures that no painkilling medication is needed |  |
| Avoiding hospitalisation | The treatment avoids hospitalisation |  |
| Avoiding surgery | The treatment ensures that no operation is necessary |  |
| Improved quality of life | The treatment provides a better quality of life |  |
| Improved work rate | The treatment enhances participation in professional activities |  |
| Characteristics related to the administration of the treatment | | |
| Mode of administration | The way the treatment is administered to the patient (e.g., by mouth, through the anus, by vein, in the muscle, in the skin ...) |  |
| Frequency of treatment | How often the patient is treated (e.g., several times a day, daily, weekly, fortnightly, four weekly, eight weekly, only in case of complaints, ...) |  |
| Frequency of follow-up | The number of times that the patient must consult a doctor to monitor the effect of the treatment (e.g., every 3 months, every 4 months, every 6 months, annually, only in the event of complaints, etc.) |  |
| Location of treatment | The place where the treatment is administered to the patient (e.g., home, hospital, ...) |  |
| Indication of IBD on the package leaflet | Some drugs are prescribed for IBD even though IBD is not mentioned in the package leaflet as a condition (indication) for which the drug was authorised (unregistered use). This means that the drug has not been tested in IBD patients during clinical trials and has not been registered by the European Medicines Agency as a drug that can be used to treat IBD. Doctors can choose to prescribe this medicine anyway, if literature and expertise show that this medicine is also effective for IBD. |  |
| Characteristics related to symptoms of the disease or complications and side effects of the treatment | | |
| Occurrence of abdominal pain and cramps | - Pain and cramps in the abdominal region - This may occur in up to 70% of chronic IBD patients with active disease and in up to 20% of chronic IBD patients in remission |  |
| Occurrence of blood in the stool | - Blood in the stool - This may occur in up to 37% of IBD patients with active disease and up to 10% of chronic IBD patients in remission |  |
| Occurrence of sudden need to relieve oneself | - Sudden compelling and necessary need to relieve - This may occur in up to 80% of IBD patients with active disease and in up to 50% of chronic IBD patients in remission |  |
| Occurrence of incontinence | - Stool cannot be held - This may occur in up to 75% of IBD patients with active disease and in up to 10% of chronic IBD patients in remission |  |
| Occurrence of indigestion | - Painful, difficult, or disturbed digestion of food and drinks through the gastrointestinal tract - This may occur in up to 45% of IBD patients with active disease and up to 10% of chronic IBD patients in remission |  |
| Occurrence of diarrhoea | - Frequent and watery stools - This may occur in up to 80% of IBD patients with active disease and in up to 40% of chronic IBD patients in remission |  |
| Occurrence of vomiting | - Vomiting of food or drink through the mouth or nose - This may occur in up to 12% of IBD patients with active disease and in up to 3% of chronic IBD patients in remission |  |
| Occurrence of nausea | - Sensation of nausea or discomfort in the stomach, possibly accompanied by an urge to vomit. - This may occur in up to 39% of IBD patients with active disease and in up to 17% of chronic IBD patients in remission |  |
| Occurrence of abnormal liver function | - Abnormal liver function. This is often diagnosed by an elevated liver function test - It can occur in up to 21% of IBD patients |  |
| Occurrence of abnormal renal function | - Abnormal kidney function - This may occur in up to 28% of IBD patients |  |
| Development or worsening of diabetes | - The development or worsening of diabetes - No data were found on how often this may occur |  |
| Changes in body weight | - Decrease or increase in body weight - This may occur in up to 85% of IBD patients |  |
| Loss of appetite | - Little or no need to take food - This may occur in up to 40% of IBD patients with active disease and in up to 20% of chronic IBD patients in remission |  |
| Occurrence of fatigue | - An overwhelming feeling of continuous fatigue, lack of energy, or a feeling of exhaustion that does not diminish after rest or sleep. - This can occur in up to 75% of chronic IBD patients with active disease and in up to 20% of chronic IBD patients in remission |  |
| Occurrence of lethargy | - Without energy or strength - No data were found on how often this may occur |  |
| Occurrence of fever | - A temperature above 37.8 degrees Celsius - No data were found on how often this may occur |  |
| Occurrence of headache | - Pain in and around the head - This may occur in up to 69% of chronic IBD patients |  |
| Occurrence of dizziness | - A feeling of instability or lightheadedness. - This can occur in up to 25% of chronic IBD patients with active disease and in up to 13% of chronic IBD patients in remission |  |
| Occurrence of neuropathy | - The malfunctioning of one or more nerves. This can cause, for example, tingling and sensations of pain in the hands and feet. - This can occur in up to 20% of chronic IBD patients |  |
| Appearance of skin rash | - A general condition where changes to the skin occur e.g., reddish discolouration of the skin, development of dry skin, eczema, acne, ... - This can occur in up to 15% of chronic IBD patients. |  |
| Occurrence of hypersensitivity to UV rays | - The development of abnormally increased reactivity of the skin or eyes to sunlight - No data have been found on how often this may occur |  |
| Occurence of hair loss | - The abnormal loss of hair - This may occur in up to 33% of chronic IBD patients |  |
| Occurrence of bone or back pain | - Pain or tenderness in the bone and/or the back - This may occur in up to 25% of chronic IBD patients |  |
| Occurrence of joint pain | - One or more body joints are swollen and/or painful - This may occur in up to 30% of chronic IBD patients |  |
| Occurrence of muscle pain | - Sensation of pain in a muscle - No data were found on how often this may occur |  |
| Development of osteoporosis | - The development of osteoporosis - This may occur in up to 50% of chronic IBD patients with active disease |  |
| Reduction of eyesight | - Impaired vision, in which vision becomes blurred and images are sometimes distorted - This is an effect of an extraintestinal manifestation in the eyes. All extraintestinal manifestations in the eyes together occur in up to 16% of chronic IBD patients |  |
| Occurrence of hypertension | - A high blood pressure isdefined by a blood pressure value higher than 140 millimetres of mercury (mm Hg) for the upper number (this is the positive pressure) and/or 90 mm Hg for the lower number (this is the negative pressure). - This may occur in up to 13% of chronic IBD patients |  |
| Occurrence of anaemia | - Too low red blood cell count and/or too low haemoglobin content - This may occur in up to 74% of chronic IBD patients |  |
| Occurrence of insomnia | - A sleep disorder that makes it difficult to fall asleep, difficult for you to stay asleep, or that causes you to wake up too early and not be able to get back to sleep. - Insomnia is a sleep disorder. All sleep disorders together occur in up to 68% of chronic IBD patients |  |
| Occurrence of depressive mood | - A mood or state of mind that causes feelings of sadness and disinterest. - This may occur in up to 25% of chronic IBD patients |  |
| Occurrence of anxiety | - Feelings of panic, anxiety, and nervousness - This may occur in up to 22% of chronic IBD patients |  |
| Occurrence of serious infections | - The entry and growth of microbes in the body due to a weakening of the immune system that can cause serious problems and require hospitalisation - This may occur in up to 6% of chronic IBD patients |  |
| Occurrence of infusion reactions | - A type of hypersensitivity reaction that occurs during or shortly after administration of a medicine. Specific reactions are itching, redness of the skin, fever, a rise in temperature, sweating and chills. - This may occur in up to 23% of chronic IBD patients |  |
| Development of cancer | - The development of a new cancer, e.g., colorectal cancer, Non-Hodgkin's lymphoma, or skin cancer - Colorectal cancer may occur in up to 2% of chronic IBD patients after 2 years, 8% after 20 years, and 18% after 30 years - No data have been found on how common other cancers may be |  |
| Development of associated inflammatory diseases | - The development or worsening of spondylarthritis, rheumatoid arthritis, psoriasis, psoriatiform arthropathy, ... - This may occur in up to 11% of chronic IBD patients |  |
| Construction of a stoma | - The construction of an artificial outlet for stools, in which the small intestine is surgically brought out and sutured to the skin - This may be necessary in up to 10% of chronic IBD patients |  |
| Construction of a pouch | - The construction of a pouch, reservoir or (collecting) bag by means of surgery. The last part of the small intestine is connected to the anus, so that nothing can be seen from the outside. The faeces pass out of the body in the normal way. - This may be necessary in up to 35% of chronic IBD patients |  |
| Missing treatment characteristics | Explanation | Grading |
|  |  |  |
|  |  |  |
|  |  |  |
|  |  |  |
|  |  |  |
