# Supplementary material for: Patient preferences for inflammatory bowel disease treatments: protocol development of a global preference survey using a discrete choice experiment
Source: Front Med (Lausanne). 2024 Aug 14;11:1418874. doi: 10.3389/fmed.2024.1418874 (PMC11349669; doi:10.3389/fmed.2024.1418874)
Supplement: Supplementary file 3 [file Table_3.DOCX]

***Supplementary Material 3***

**Patient Preferences for Inflammatory Bowel Disease Treatments: Protocol Development of a Global Preference Survey using a Discrete Choice Experiment**

# Participants’ grading of treatment characteristics in order of importance from highest to lowest importance

Participants were asked to grade the characteristics as a preparation for the discussion according to how important they found them, on a scale from 1 – 10, with 10 being the most important.

| **Rank** | **Characteristic** | **Mean score** | **Standard deviation** | **Maximal score** | **Minimal score** |
| --- | --- | --- | --- | --- | --- |
| 1 | Prevent surgery | 9,55 | 0,82 | 10 | 8 |
| 2 | Long-term clinical remission | 9,45 | 1,21 | 10 | 6 |
| 3 | Improved quality of life | 9,36 | 1,03 | 10 | 7 |
| 4 | Improved labor rate | 9,27 | 1,10 | 10 | 7 |
| 5 | Occurrence of urgency | 9,27 | 1,01 | 10 | 7 |
| 6 | Occurrence of diarrhea | 9,18 | 1,08 | 10 | 7 |
| 7 | Occurrence of severe infections | 9,18 | 1,25 | 10 | 6 |
| 8 | Prevent hospitalization | 9,09 | 1,45 | 10 | 6 |
| 9 | Prevent flare-ups | 9,00 | 1,79 | 10 | 4 |
| 10 | Occurrence of joint pain | 9,00 | 1,26 | 10 | 6 |
| 11 | Use of corticosteroids | 9,00 | 2,10 | 10 | 3 |
| 12 | Occurrence of incontinence | 8,82 | 1,54 | 10 | 5 |
| 13 | Occurrence of hair loss | 8,73 | 2,37 | 10 | 2 |
| 14 | Macroscopic healing of the intestinal mucosa | 8,64 | 2,16 | 10 | 4 |
| 15 | Occurence of reduced vision | 8,64 | 2,69 | 10 | 1 |
| 16 | Occurrence of fatigue | 8,55 | 2,16 | 10 | 3 |
| 17 | Occurrence of lethargy | 8,45 | 2,70 | 10 | 1 |
| 18 | Short-term clinical response | 8,36 | 2,06 | 10 | 5 |
| 19 | Occurrence of abnormal kidney function | 8,36 | 2,38 | 10 | 4 |
| 20 | Occurrence of bone and back pain | 8,36 | 2,62 | 10 | 1 |
| 21 | Occurrence of depressive mood | 8,36 | 2,91 | 10 | 1 |
| 22 | Development of cancer | 8,36 | 3,64 | 10 | 1 |
| 23 | Construction of a pouch | 8,36 | 3,64 | 10 | 1 |
| 24 | Development or worsening of diabetes | 8,27 | 3,61 | 10 | 1 |
| 25 | Occurrence of fear | 8,27 | 2,33 | 10 | 3 |
| 26 | Microscopic healing of the intestinal mucosa | 8,09 | 2,30 | 10 | 4 |
| 27 | Transmural healing of the gut | 8,09 | 2,30 | 10 | 4 |
| 28 | Occurrence of fever | 8,09 | 2,81 | 10 | 1 |
| 29 | Development of osteoporosis | 8,09 | 3,56 | 10 | 1 |
| 30 | Construction of a stoma | 8,09 | 3,62 | 10 | 1 |
| 31 | Changes in body weight | 8,00 | 2,00 | 10 | 3 |
| 32 | Occurrence of anemia | 8,00 | 3,10 | 10 | 1 |
| 33 | Occurrence of insomnia | 8,00 | 2,72 | 10 | 1 |
| 34 | Occurrence of nausea | 7,91 | 2,63 | 10 | 1 |
| 35 | Occurrence of abdominal pain and cramps | 7,82 | 2,44 | 10 | 2 |
| 36 | Occurrence of dizziness | 7,82 | 3,12 | 10 | 1 |
| 37 | Occurrence of hypertension | 7,82 | 3,03 | 10 | 1 |
| 38 | Occurrence of abnormal liver function | 7,73 | 3,52 | 10 | 1 |
| 39 | Occurrence of headache | 7,73 | 2,87 | 10 | 1 |
| 40 | Development of associated inflammatory conditions | 7,73 | 3,55 | 10 | 1 |
| 41 | Occurrence of bloody stools | 7,64 | 2,38 | 10 | 2 |
| 42 | Occurrence of neuropathy | 7,64 | 3,47 | 10 | 1 |
| 43 | Occurrence of skin rash | 7,55 | 3,27 | 10 | 1 |
| 44 | Occurrence of vomiting | 7,45 | 3,39 | 10 | 1 |
| 45 | Use of analgesic medication | 7,36 | 2,42 | 10 | 2 |
| 46 | Occurrence of indigestion | 7,36 | 2,94 | 10 | 2 |
| 47 | Occurrence of hypersensitivity to UV rays | 7,27 | 2,97 | 10 | 1 |
| 48 | Occurrence of muscle strain | 7,27 | 2,83 | 10 | 1 |
| 49 | Frequency of follow-up | 7,09 | 1,45 | 9 | 4 |
| 50 | Frequency of treatment | 6,91 | 1,64 | 9 | 4 |
| 51 | Place of treatment | 6,55 | 2,21 | 9 | 2 |
| 52 | Indication of IBD on the package leaflet | 6,55 | 2,30 | 10 | 3 |
| 53 | Method of administration | 6,27 | 3,07 | 10 | 1 |
| 54 | Loss of appetite | 6,27 | 3,23 | 10 | 1 |
| 55 | Occurrence of infusion reactions | 6,27 | 3,41 | 10 | 1 |
