# Supplementary material for: Patient preferences for inflammatory bowel disease treatments: protocol development of a global preference survey using a discrete choice experiment
Source: Front Med (Lausanne). 2024 Aug 14;11:1418874. doi: 10.3389/fmed.2024.1418874 (PMC11349669; doi:10.3389/fmed.2024.1418874)

***Supplementary Material 4***

**Patient Preferences for Inflammatory Bowel Disease Treatments: Protocol Development of a Global Preference Survey using a Discrete Choice Experiment**

# Final patient preference survey


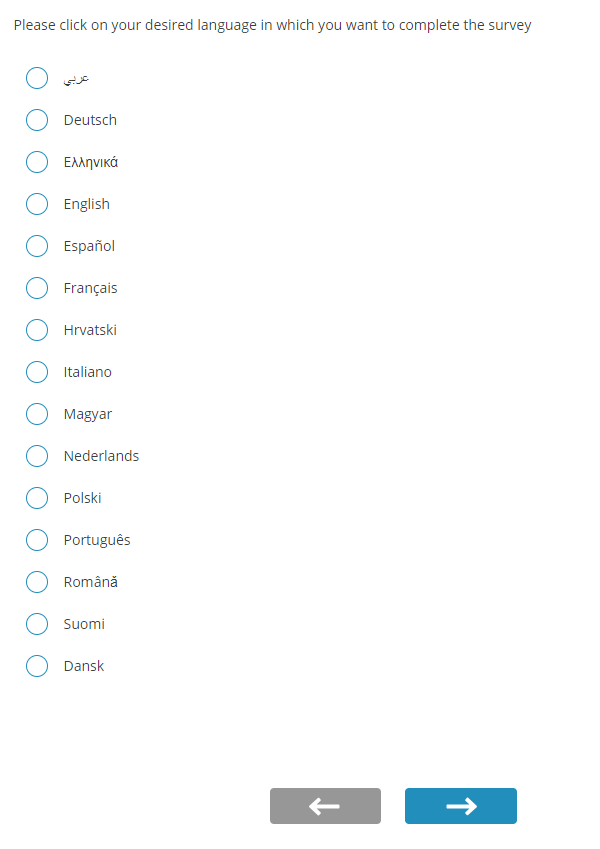


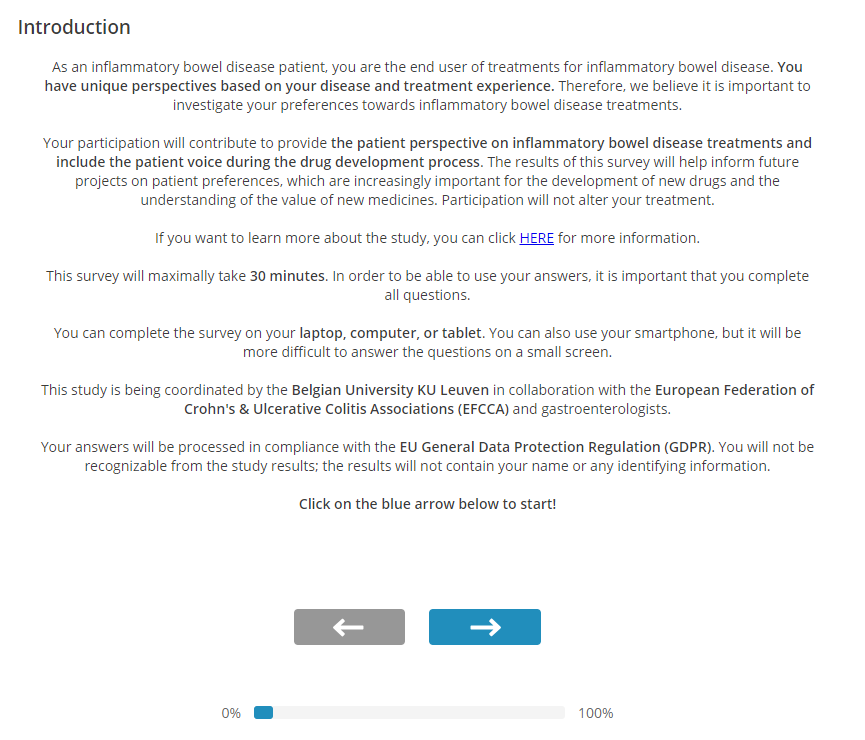


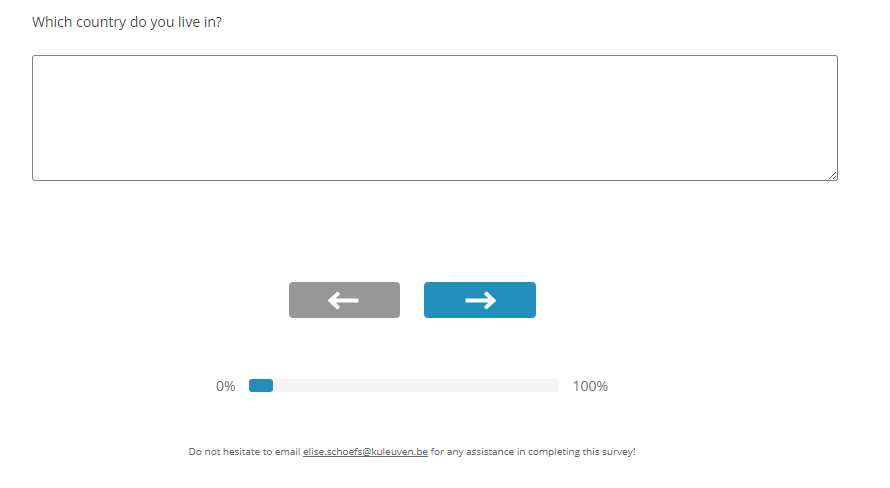


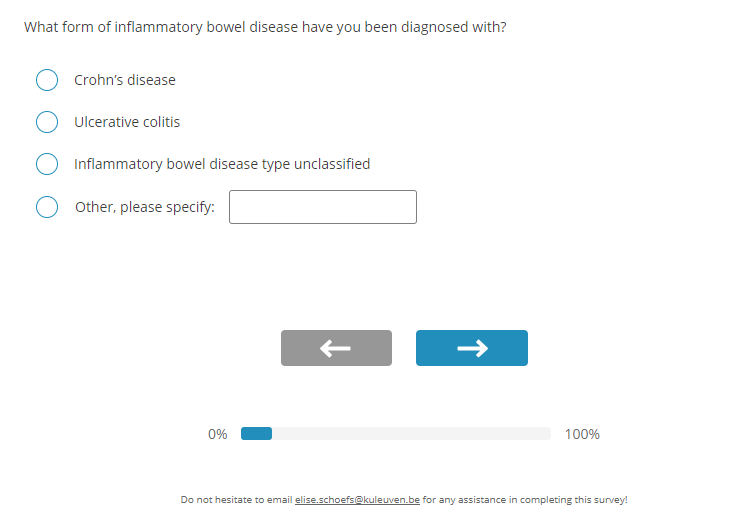


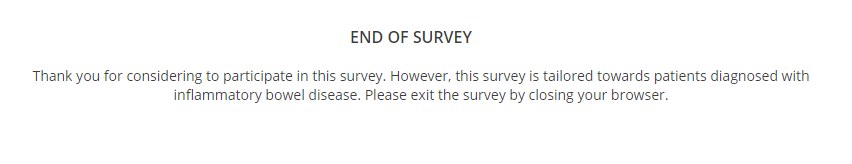


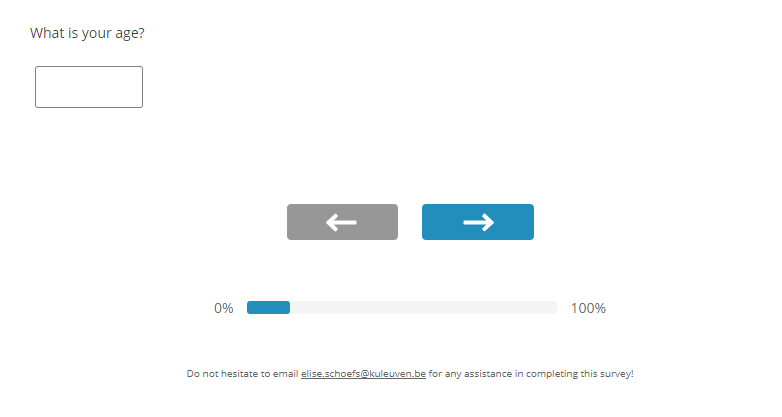


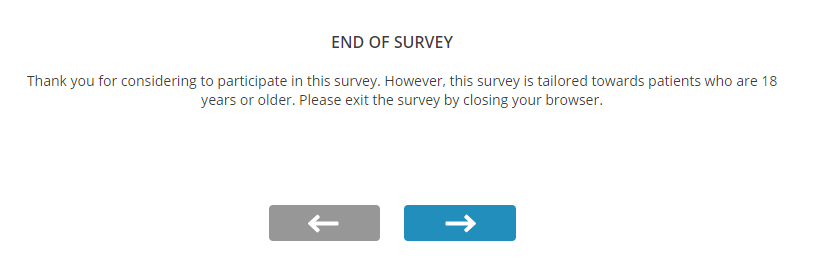


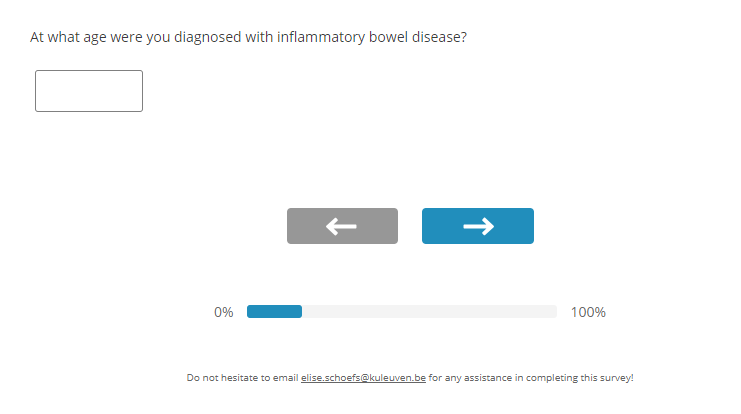


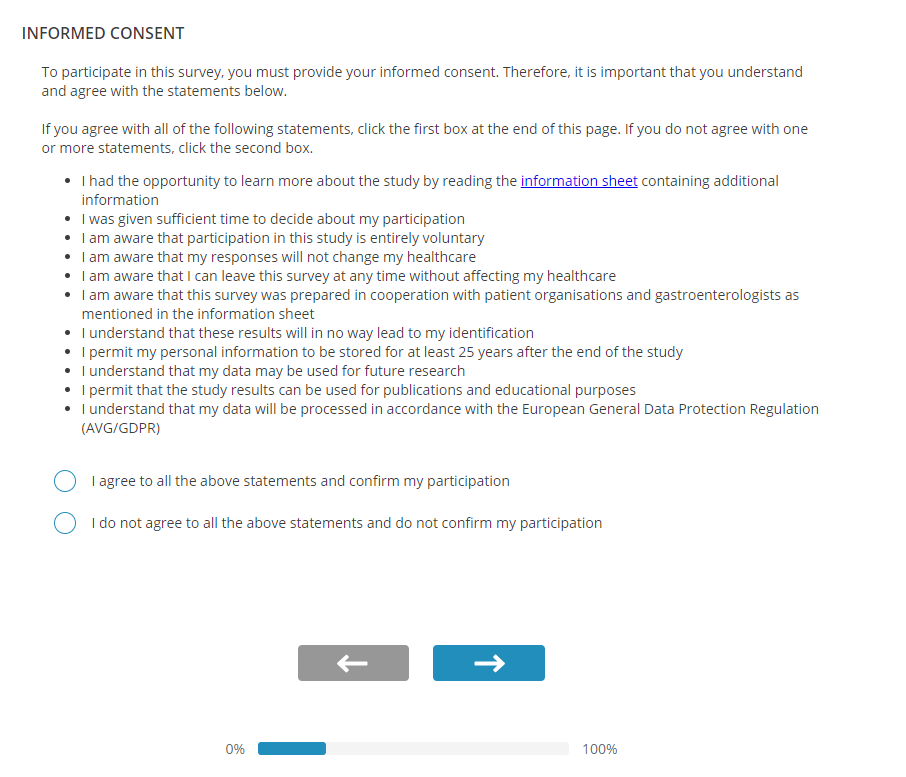


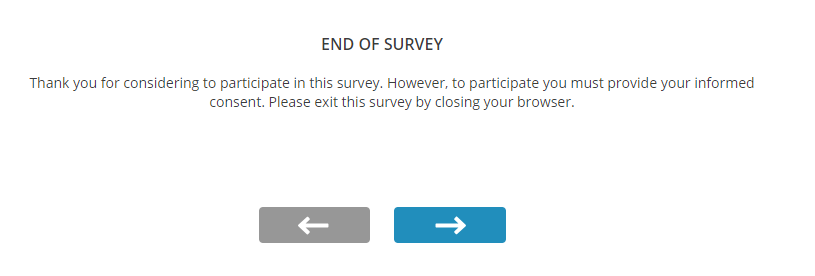


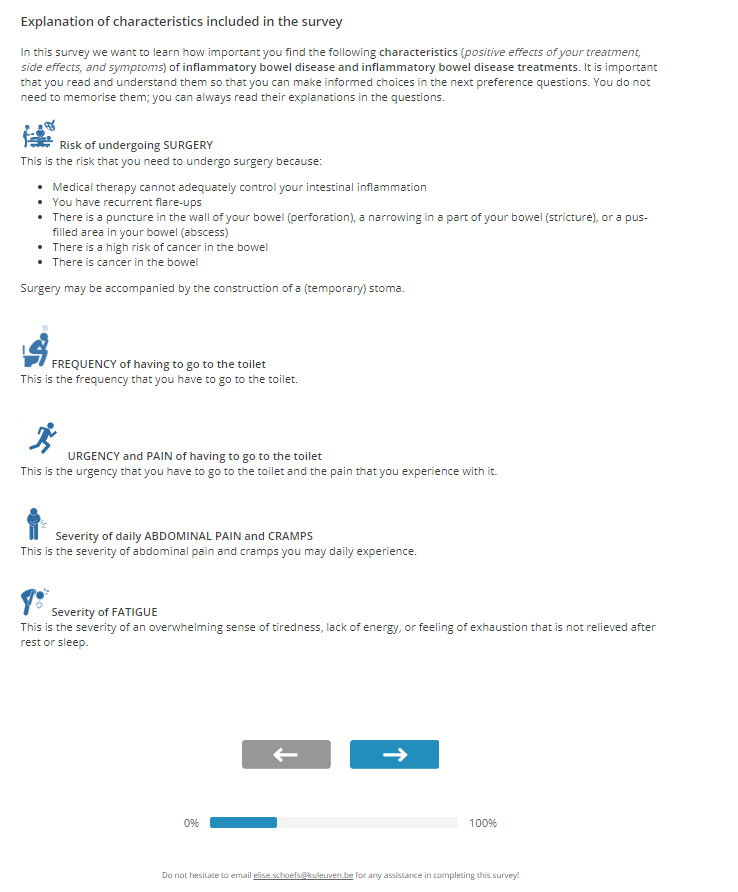


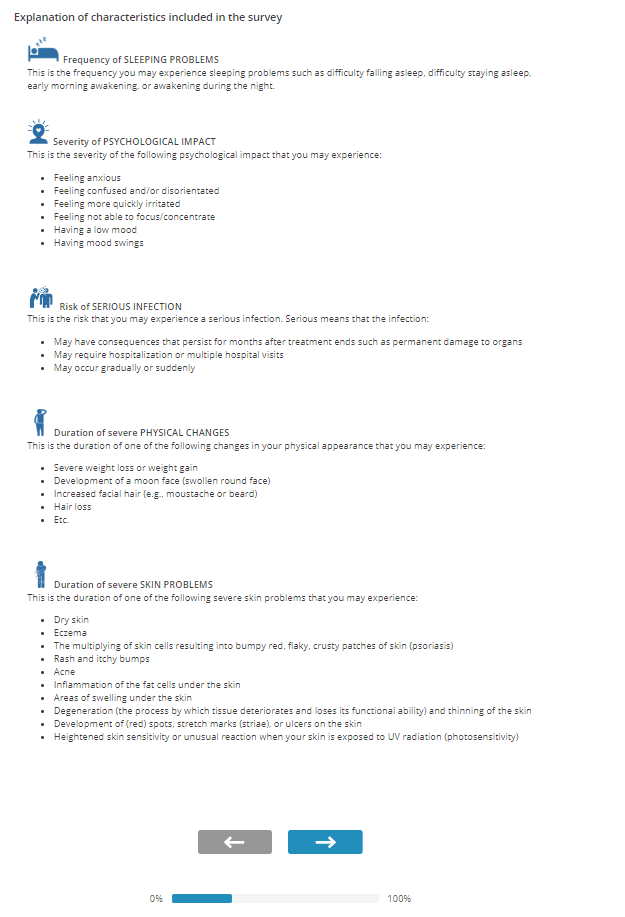


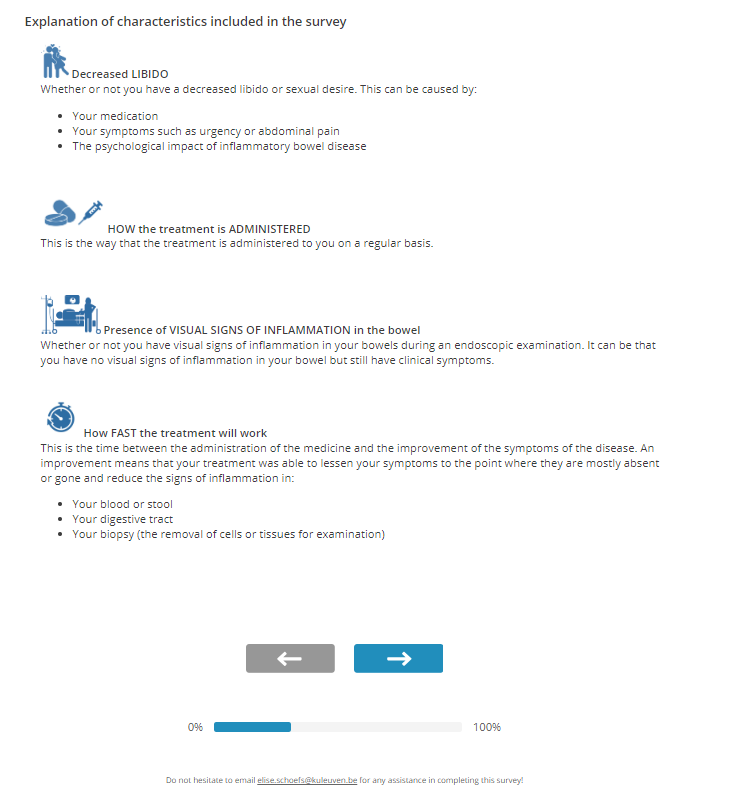


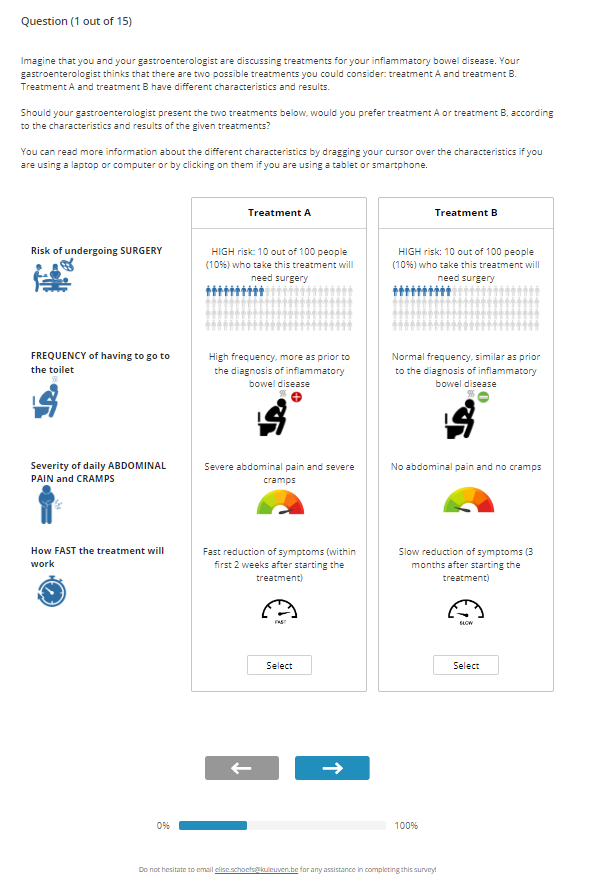


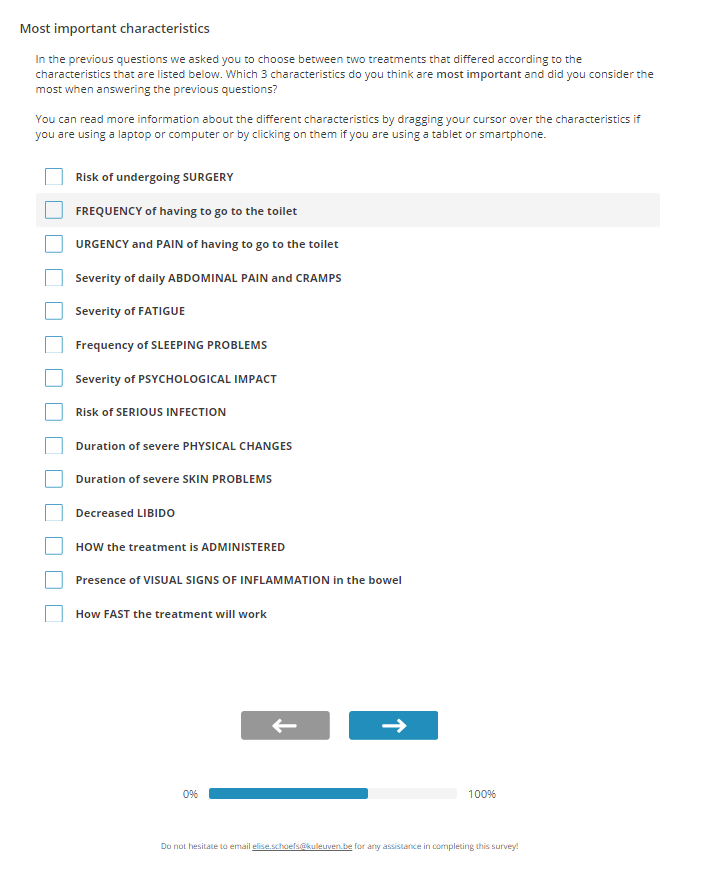


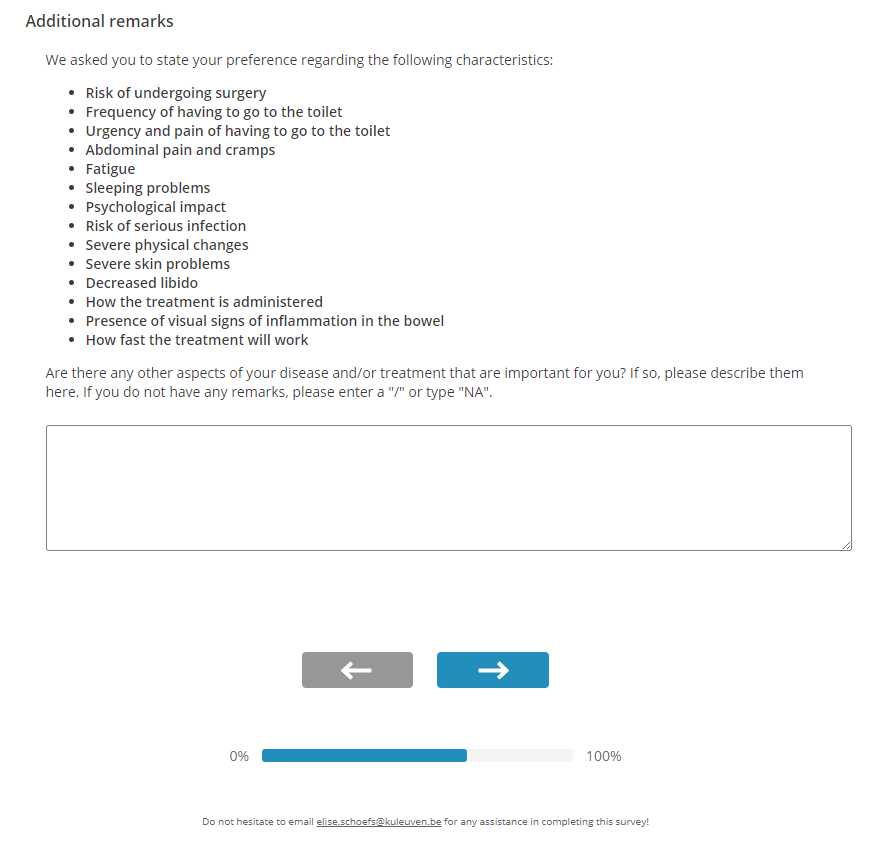


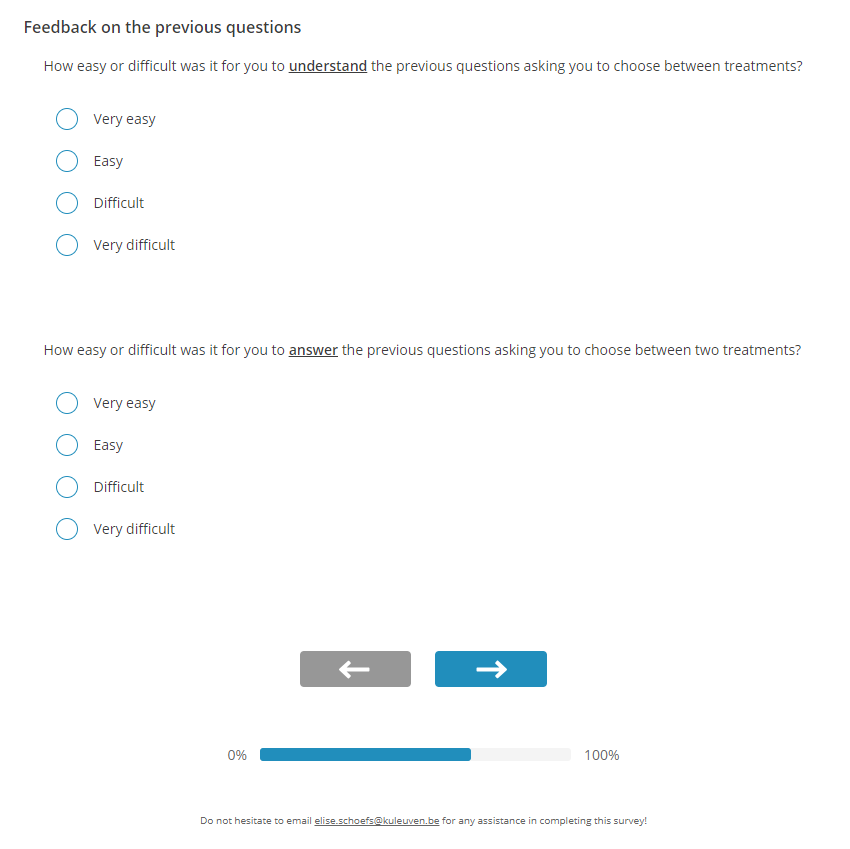


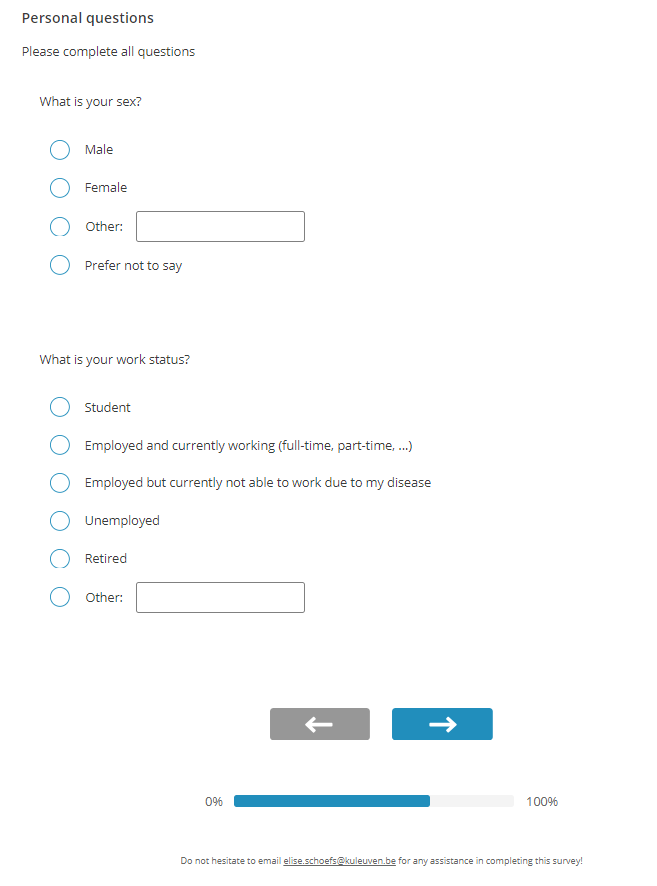


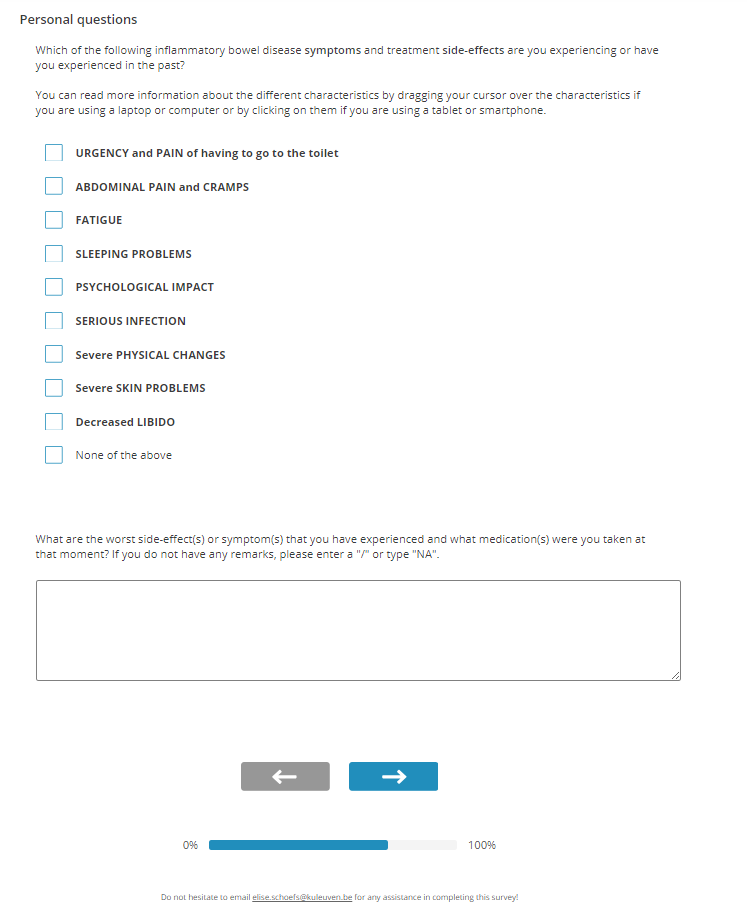


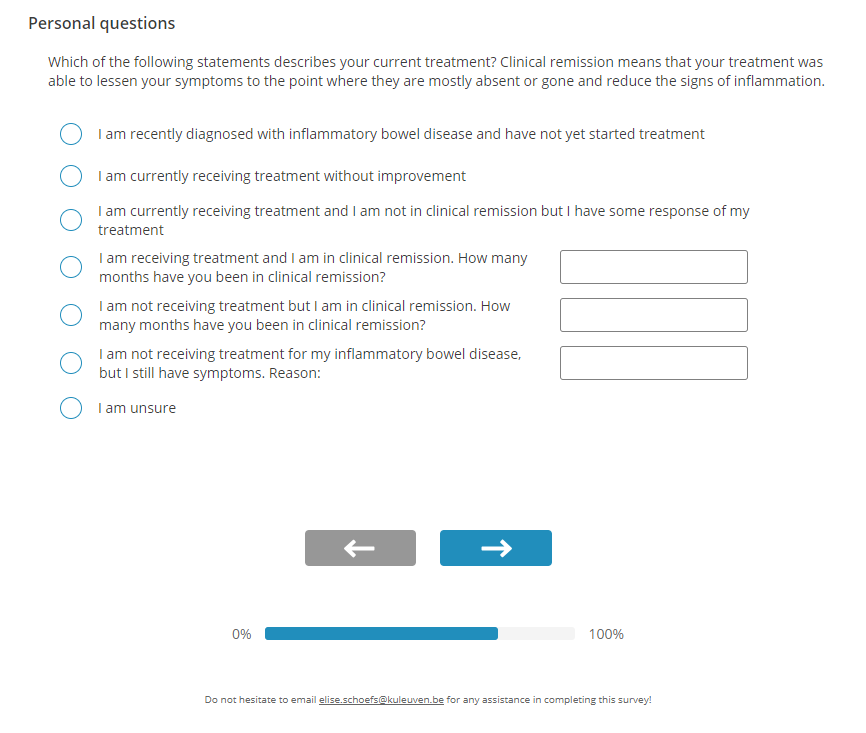


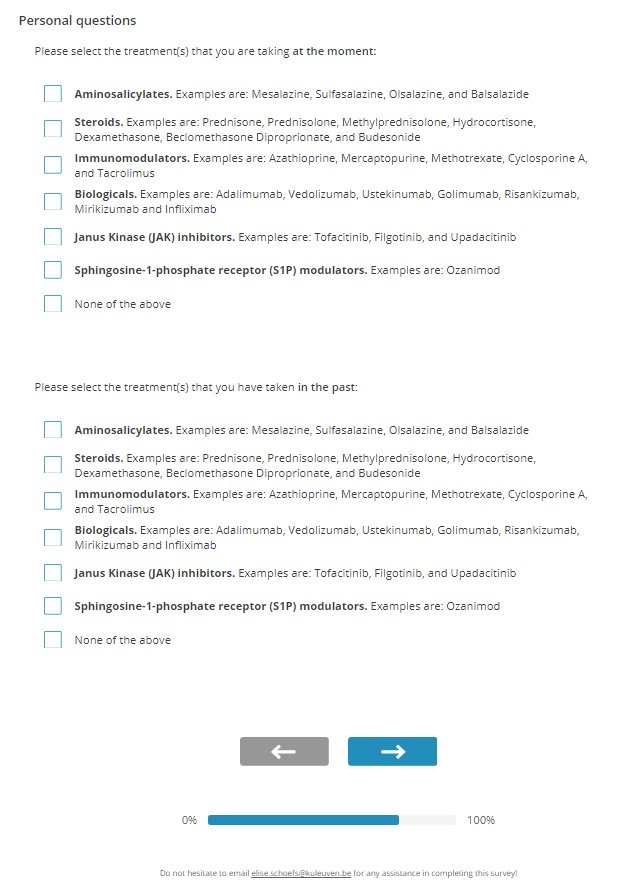


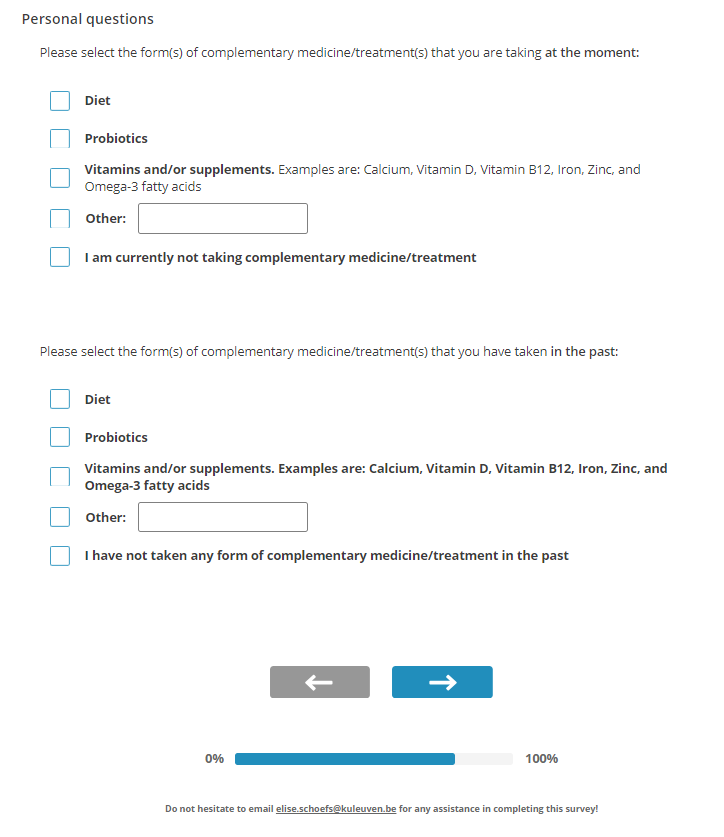


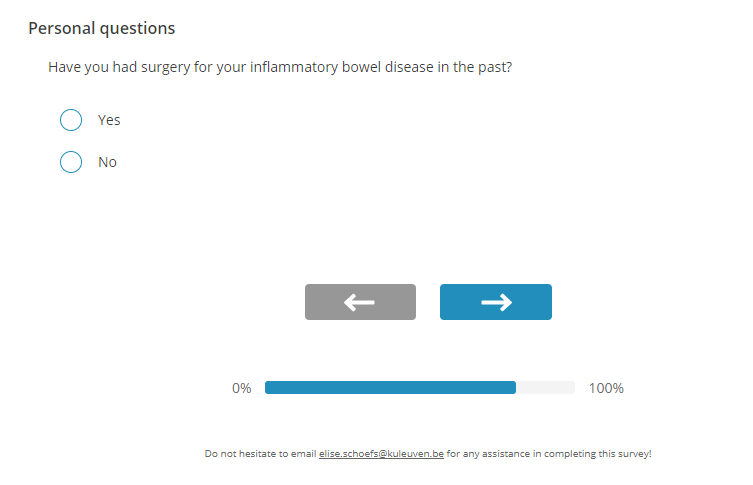


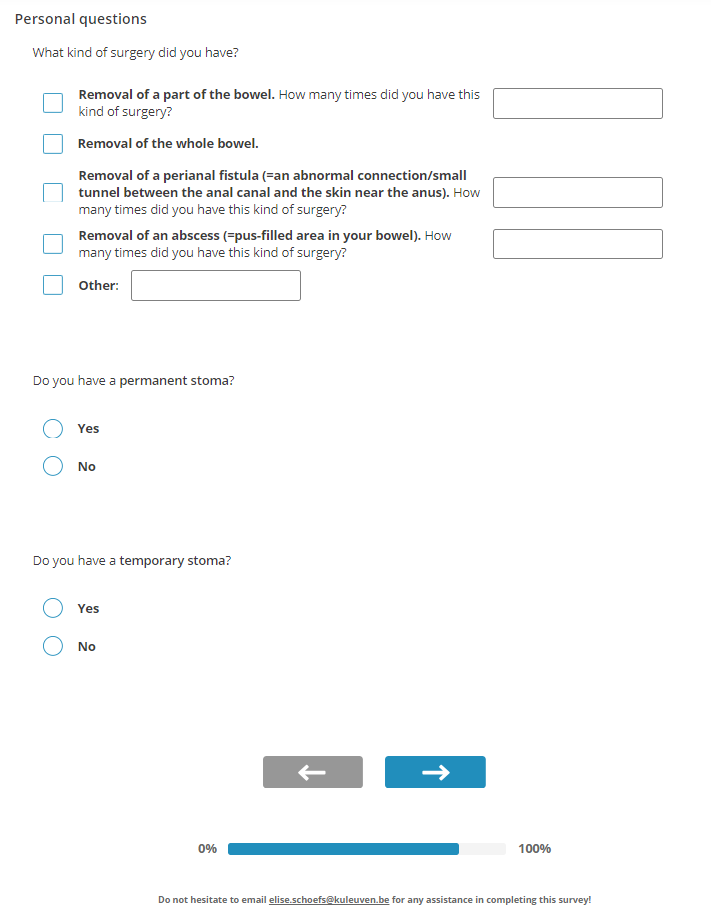


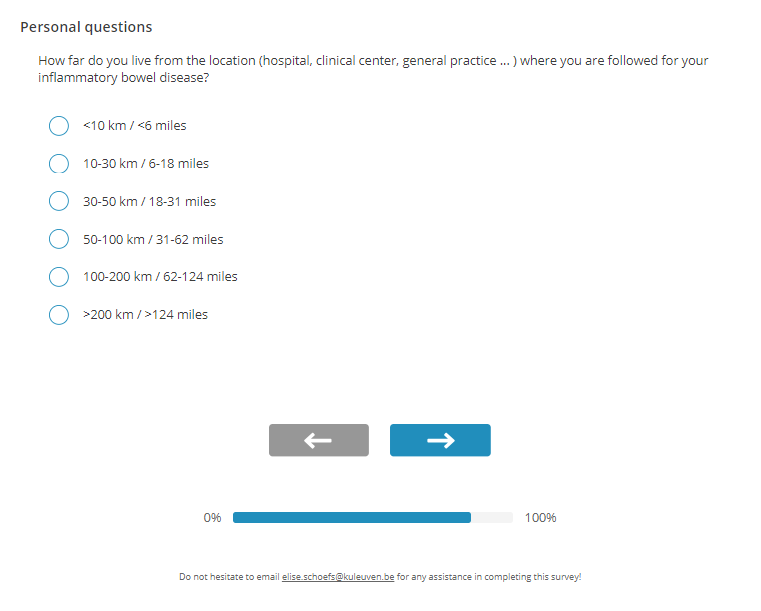


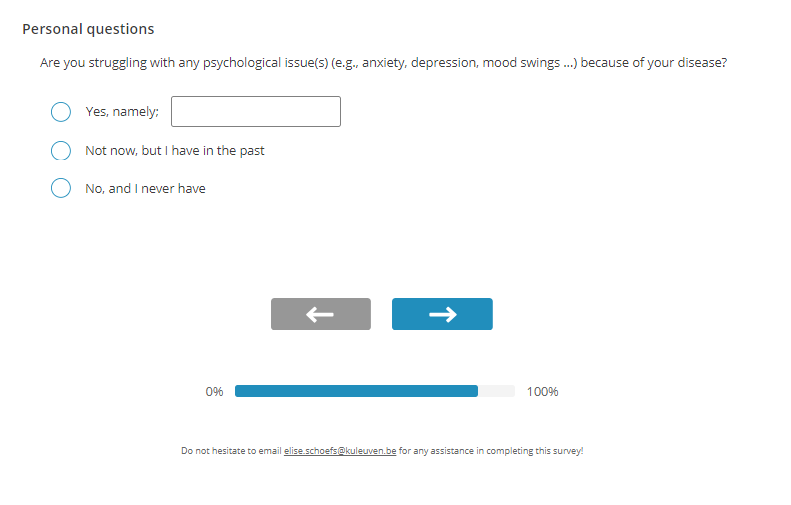


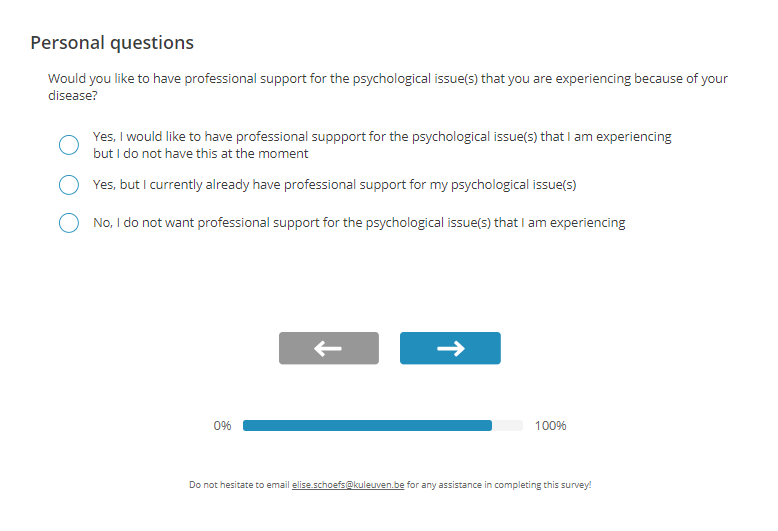


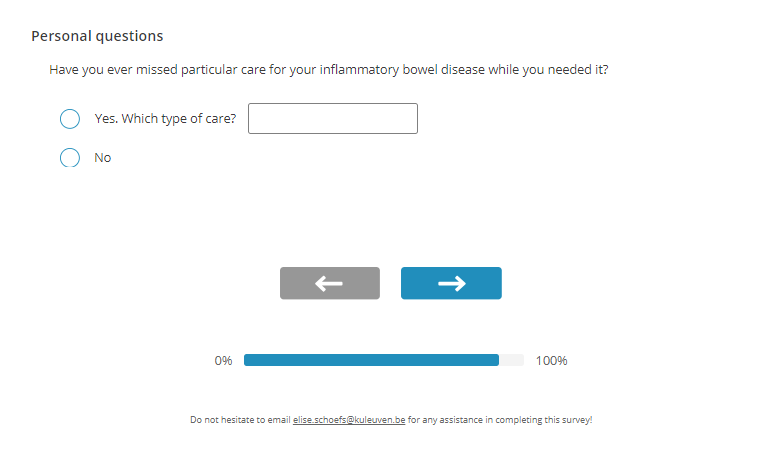


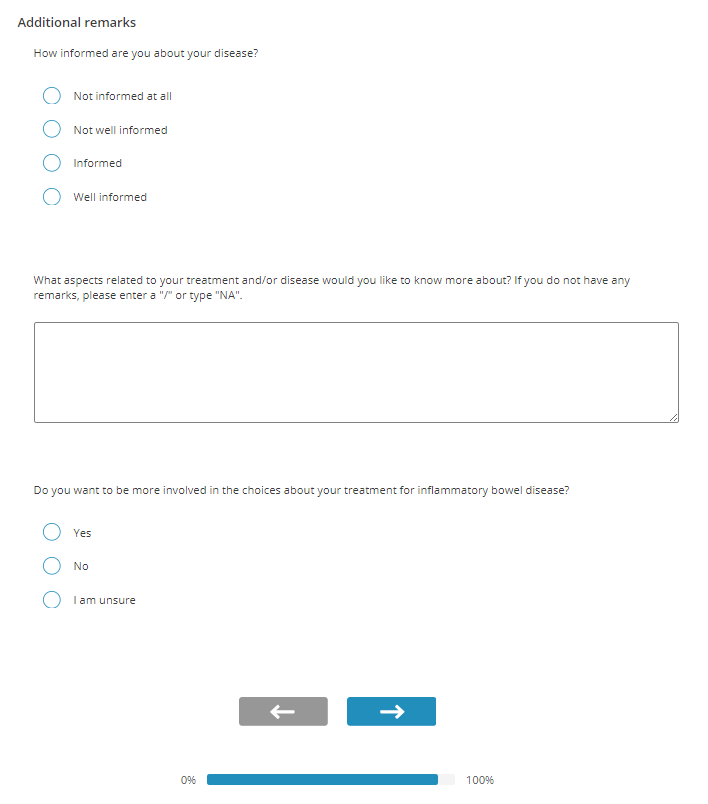


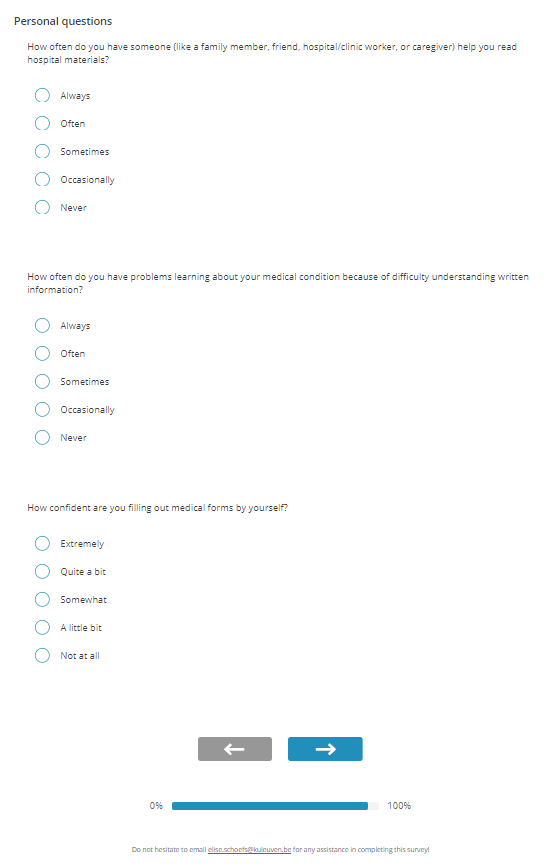


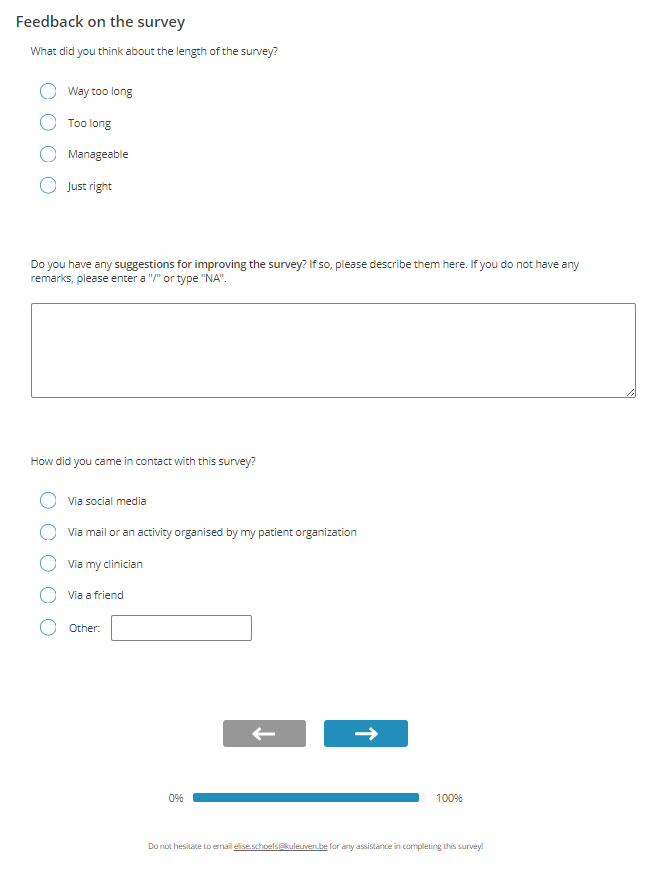


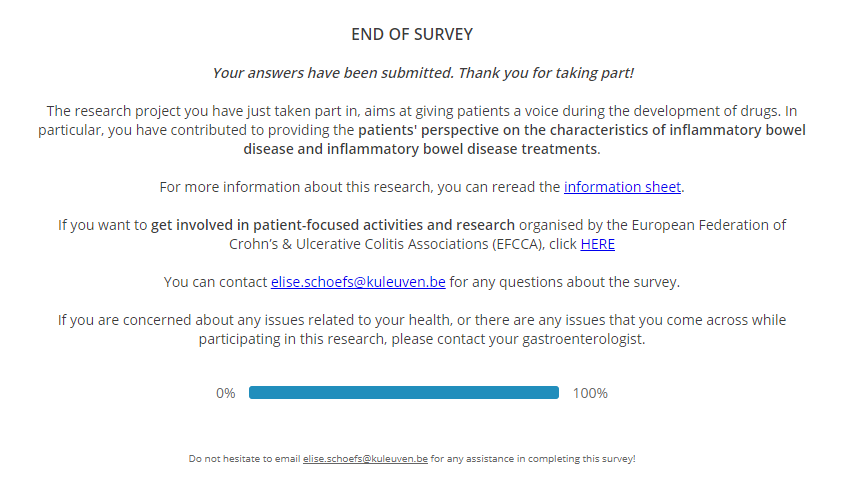

Supplement: Supplementary file 4 [file Table_4.DOCX]
